# Supplementary material for: Associations between metal constituents of ambient particulate matter and mortality in England: an ecological study
Source: BMJ Open. 2019 Dec 3;9(12):e030140. doi: 10.1136/bmjopen-2019-030140 (PMC6924721; doi:10.1136/bmjopen-2019-030140)

Supplementary material to:

Associations between metal constituents of ambient particulate matter and mortality in England; an ecological study

Table S1: Descriptive statistics of health outcomes, modelled particulate metal concentrations, deprivation score, and ethnicity covariates for the 1533 wards in the study area in 2008-11.

	10th centile	mean	median	90th centile	LOOCV* R^2 (for LUR**)
**Health outcomes**	Rates of health outcome (number of cases per hundred thousand people)				
Cardiovascular mortality	117.50	215.97	203.20	327.87	
Respiratory mortality	42.85	96.34	87.85	160.41	
Lung cancer incidence	25.06	48.44	45.75	75.86	
**Modelled metal concentrations using LUR**	Metals in ng/m^3				
Cu PM_{10}	7.0	13.3	13.1	19.8	0.95
Fe PM_{10}	223.2	378.9	357.0	596.7	0.95
Zn PM_{10}	113.5	135.2	139.5	153.0	0.77
Cu $PM_{2.5}$	2.6	4.3	4.6	5.7	0.79
Fe $PM_{2.5}$	51.6	86.8	82.8	129.0	0.92
**Area-level confounders**					
Deprivation (modified IMD)	3.45	7.08	6.47	11.78	
% of Asian	2	13	9	33	
% of White	38	72	77	95	
Tobacco expenditure (pounds/week/inhabitant)	3.40	4.61	4.48	6.03	

*LOOCV Leave one out cross validation

** LUR Land Use Regression

Table S2: Poisson regression confounder effects from the two models (i) using metals from PM_{10} and (ii) metals from $PM_{2.5}$ for all the health outcomes. Mean, lower and upper bound of the 95% credible interval (CI) of the inter-decile relative risk (RR).

Outcomes	Model	Confounders	RR	CI 95%
Cardiovascular mortality	All Metals in PM_{10} VIF[2]=9.14	IMD[1]	1.098	(1.02,1.182)
		% Asian	0.982	(0.921,1.046)
		% White	0.817	(0.729,0.915)
		Tobacco expenditure	1.197	(1.133,1.265)
	All Metals in $PM_{2.5}$ VIF[2]=9.04	IMD[1]	1.095	(1.02,1.177)
		% Asian	0.987	(0.926,1.052)
		% White	0.824	(0.737,0.922)
		Tobacco expenditure	1.192	(1.135,1.253)
		IMD[1]	1.188	(1.073,1.315)

Respiratory mortality	All Metals in PM_{10} VIF[2]=8.93	% Asian	0.887	(0.813,0.967)
		% White	0.822	(0.704,0.959)
		Tobacco expenditure	1.301	(1.206,1.403)
	All Metals in $PM_{2.5}$ VIF[2]=8.81	IMD[1]	1.183	(1.07,1.306)
		% Asian	0.892	(0.817,0.973)
		% White	0.846	(0.725,0.986)
		Tobacco expenditure	1.301	(1.214,1.393)
Lung cancer incidence	All Metals in PM_{10} VIF =8.60	IMD[1]	1.390	(1.261,1.532)
		% Asian	0.851	(0.790,0.916)
		% White	0.932	(0.818,1.062)
		Tobacco expenditure	1.472	(1.366,1.586)
	All Metals in $PM_{2.5}$ VIF[2]=7.72	IMD[1]	1.404	(1.276,1.544)
		% Asian	0.846	(0.786,0.910)
		% White	0.955	(0.839,1.086)
		Tobacco expenditure	1.468	(1.373,1.569)

[1]IMD Indices of multiple deprivation
[2]VIF Variance Inflation Factor

Table S3: Pearson Correlation(r) between the adjusted annual mean concentrations of PM-metals and the adjusted annual mean PM concentrations ($PM_{2.5}$ and PM_{10}).

N=1533		$PM_{2.5}$ CU	$PM_{2.5}$ FE	PM_{10} CU	PM_{10} FE	PM_{10} ZN	$PM_{2.5}$	PM_{10}
**$PM_{2.5}$**	Correlation	0.86	0.89	0.89	0.89	0.73	1.00	0.92
**PM_{10}**	Correlation	0.82	0.87	0.86	0.88	0.74	0.92	1.00

All the correlation significant at p< 0.001

Figure S1. A graphical presentation of the confounding and causal mechanism, linking exposure and adverse health outcomes.

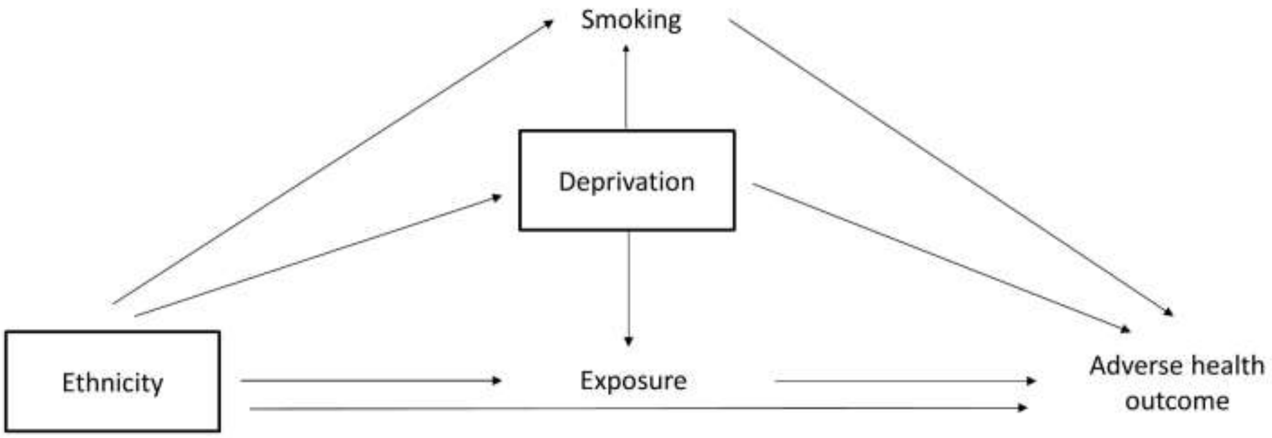

Figure S2. Maps of the confounders in quintiles: proportion of Asian people, proportion of white people, modified index of multiple deprivations, and tobacco spends. Contains National Statistics data © Crown copyright and database right 2018; Contains OS data © Crown copyright and database right 2018. All rights reserved.

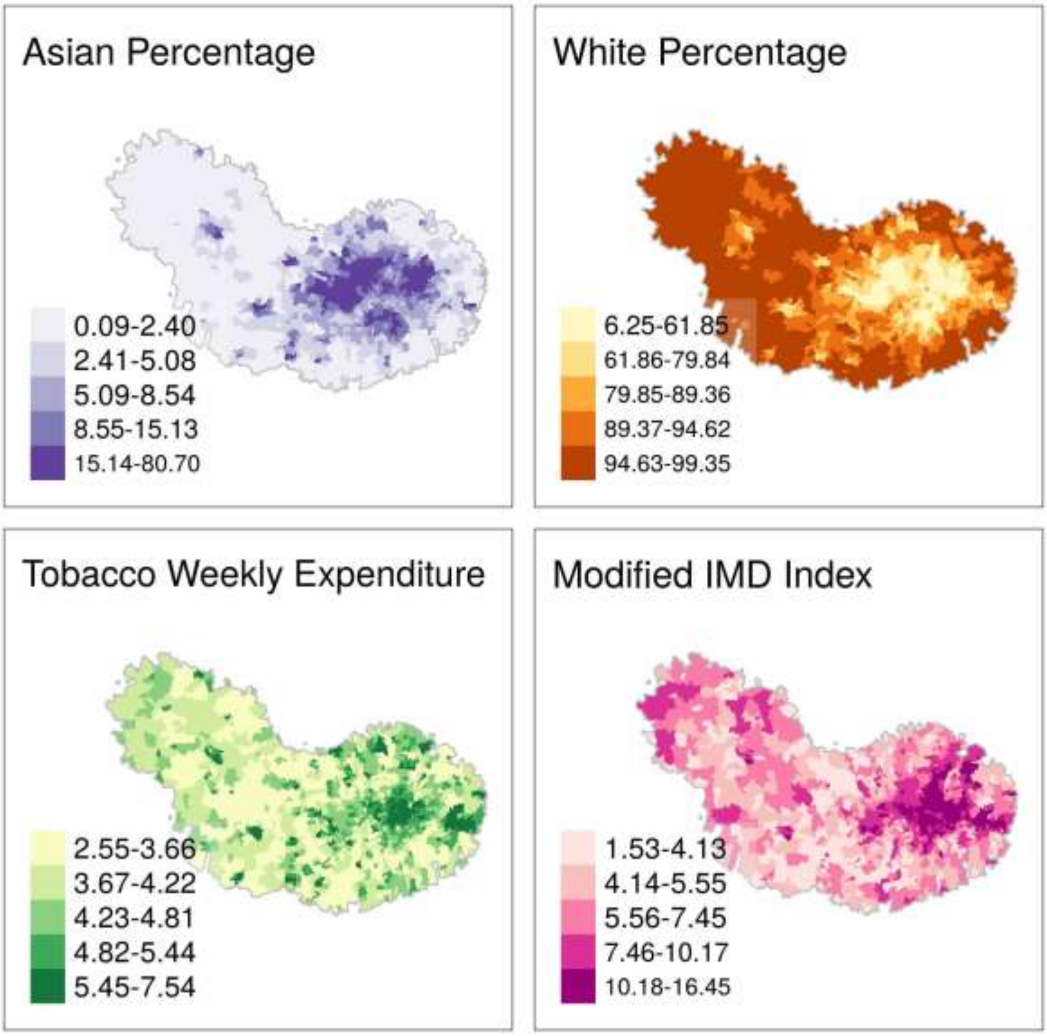

Supplement: Supplementary data [file bmjopen-2019-030140supp001.pdf]
