# Supplementary material for: Associations between metal constituents of ambient particulate matter and mortality in England: an ecological study
Source: BMJ Open. 2019 Dec 3;9(12):e030140. doi: 10.1136/bmjopen-2019-030140 (PMC6924721; doi:10.1136/bmjopen-2019-030140)
Supplement: Author's manuscript [file bmjopen-2019-030140.draft_revisions.pdf]

BMJ Open

BMJ Open is committed to open peer review. As part of this commitment we make the peer review history of every article we publish publicly available.

When an article is published we post the peer reviewers' comments and the authors' responses online. We also post the versions of the paper that were used during peer review. These are the versions that the peer review comments apply to.

The versions of the paper that follow are the versions that were submitted during the peer review process. They are not the versions of record or the final published versions. They should not be cited or distributed as the published version of this manuscript.

BMJ Open is an open access journal and the full, final, typeset and author-corrected version of record of the manuscript is available on our site with no access controls, subscription charges or pay-per-view fees (http://bmjopen.bmj.com).

If you have any questions on BMJ Open's open peer review process please email info.bmjopen@bmj.com

BMJ Open

# Associations between metal constituents of ambient particulate matter and mortality in England; a small area study

| | |
|---|---|
| Journal: | *BMJ Open* |
| Manuscript ID | bmjopen-2019-030140 |
| Article Type: | Research |
| Date Submitted by the Author: | 28-Feb-2019 |
| Complete List of Authors: | Lavigne, Aurore; Université de Lille 3 UFR MIME, UFR MIME, Domaine universitaire du Pont de Bois<br>Freni Sterrantino , Anna ; Imperial College London, Epidemiology and Biostatistics<br>Liverani, Silvia; Queen Mary University of London, School of Mathematical Sciences<br>Blangiardo, Marta; Imperial College London, MRC-PHE Centre for Environment and Health, Department of Epidemiology and Biostatistics, School of Public Health<br>de Hoogh, Kees; Swiss Tropical and Public Health Institute; University of Basel<br>Molitor, John ; Oregon State University CAPS, School of Biological and Population health sciences Biostatistics<br>Hansell, Anna ; University of Leicester, |
| Keywords: | Particulate matter elements, multipollutant effect, correlation, Metals, Environmental Epidemiology, EPIDEMIOLOGY |
| | |

SCHOLARONE™
Manuscripts

**Research Article**

# Associations between metal constituents of ambient particulate matter and mortality in England; a small area study

Aurore Lavigne*, Université Lille 3, UFR MIME, Domaine Universitaire du Pont de Bois, Villeneuve d'ascq Cedex, France

Anna Freni-Sterrantino*, Small Area Health Statistics Unit, Imperial College London, United Kingdom

Silvia Liverani, School of Mathematical Science, Queen Mary University of London, United Kingdom

Marta Blangiardo, Department of Epidemiology and Biostatistics, Imperial College London, United Kingdom

Kees de Hoogh, Swiss Tropical and Public Health Institute, Basel, Switzerland

John Molitor, School of Biological and Population health sciences Biostatistics, Oregon State University, Corvallis, OR, USA

Anna L. Hansell, Centre for Environmental Health and Sustainability, George Davies Centre, Dept of Health Sciences, University of Leicester, United Kingdom

Small Area Health Statistics Unit, Imperial College London, United Kingdom
**Corresponding author: Anna L. Hansell,**

Centre for Environmental Health and Sustainability, George Davies Centre, Dept of Health Sciences,
University of Leicester, United Kingdom 15 Lancaster Rd, Leicester LE1 7HA
Email: ah618@leicester.ac.uk
* joint first authors

The authors report no conflict of interest

**Availability of computing code and data:** The mortality cancer and population data used in this article were supplied by the Office for National Statistics (ONS), derived from the national mortality, cancer and birth registrations and the Census. SAHSU does not have permission to supply data to third parties, but the health and population data can be obtained from ONS on application. Air pollution estimates by ward for 2008-11 for the study area and code used can be obtained on request from the authors.
No identifiable information will be shared with any other organization. The scripts used can be provided by request from the authors.

**Acknowledgements**

For peer review only

We would like to acknowledge and thank Prof. John Gulliver, Dr Gary Fuller, Dr David Morley and Prof. Nicky Best for their useful comments.
CACI tobacco expenditure data is © Copyright 1996-2014 CACI Limited.

**Ethics and Information Governance statement**

SAHSU holds approvals from the National Research Ethics Service - reference 12/LO/0566 and 12/LO/0567 - and from the Health Research Authority Confidentially Advisory Group (HRA-CAG) for Section 251 support (HRA - 14/CAG/1039) for use of the health data used in this research.

The research was funded/part funded by the National Institute for Health Research Health Protection Research Unit (NIHR HPRU) in Health Impact of Environmental Hazards at King's College London in partnership with Public Health England (PHE) and Imperial College London. The views expressed are those of the author(s) and not necessarily those of the NHS, the NIHR, the Department of Health & Social Care or Public Health England".

**Funding**

The research project was funded through Medical Research Council (grant G09018401) and the Small Area Health Statistics Unit. The work of the UK Small Area Health Statistics Unit is funded by Public Health England as part of the MRC-PHE Centre for Environment and Health, funded also by the UK Medical Research Council.
The air pollution exposure assessments used in the research leading to these results was funded by the European Community's Seventh Framework Program (FP7/2007-2011) projects ESCAPE (grant agreement 211250) and TRANSPHORM (ENV.2009.1.2.2.1).

**Author's Contribution**

AL and AFS drafted the paper and ran the statistical analyses. All the authors provided intellectual input, interpreted the results, and helped to revise the manuscript. All authors approved the final version of the manuscript and agreed to be accountable for all the aspects of the work in ensuring that questions related to the accuracy or integrity of any part of the work are appropriately investigated and resolved. AH is the guarantor of this paper.

**Word Count: 2198**

**Figures: 2**

**Tables: 3**

For peer review only

For peer review only

**Abstract**

**Objectives** To investigate long-term associations between metal components of particulate matter and mortality and lung cancer incidence

**Design** Small area (ecological) study

**Setting** Population living in all wards (~9000 individuals per ward) in the London and Oxford area of England, comprising 13.6 million individuals

**Exposure and Outcome measures** We used land use regression (LUR) models originally used in the Transport related Air Pollution and Health impacts – Integrated Methodologies for Assessing Particulate Matter (TRANSPHORM) study to estimate exposure to copper, iron and zinc in ambient air particulate matter. We examined associations of metal exposure with Office for National Statistics mortality data for cardiovascular and respiratory disease and with lung cancer incidence in 2008-11

**Results** There were 108,478 CVD deaths, 48,483 respiratory deaths and 24,849 incident cases of lung cancer in the study period and area. Using Poisson regression models adjusted for area-level deprivation, tobacco sales and ethnicity, we found associations between cardiovascular mortality and $PM_{2.5}$ copper as interdecile range (IDR) Relative risk (RR) 1.005 (95%CI 1.001, 1.009) and between respiratory mortality and $PM_{10}$ zinc RR 1.136 (95%CI 1.010, 1.277). We did not find relevant associations for lung cancer incidence. Metal elements were highly correlated.

**Conclusion** Our analysis showed small but not fully consistent adverse associations between mortality and particulate metal exposures likely derived from non-tailpipe road traffic emissions (brake and tyre-wear), which have previously been associated with increases in inflammatory markers in the blood.

**Keywords:** Particulate matter elements, multipollutant effect, correlation, Metals, Environmental Epidemiology

For peer review only

**Strengths and limitations of this study**

- One of the largest studies to explore exposure to metal components of ambient air in relation to mortality and lung cancer incidence, with 13.6 million population
- A large number of cases: 108,478 CVD deaths, 48,483 respiratory deaths and 24,849 incident cases of lung cancer in the study period and area, providing good statistical power to examine small excess risks
- Established exposure models, developed and evaluated with measurements from a standardised monitoring campaign
- An ecological study using registry data, without access to individual-level confounders other than age and sex
- Metals were very highly correlated so multi-pollutant models could not be used

For peer review only

## Introduction

Long-term exposure to fine particulate matter (PM) with aerodynamic diameter less than 10µm ($PM_{10}$)[1-3] and 2.5µm ($PM_{2.5}$)[4] is associated with increased mortality levels from cardiovascular disease[1 5]. Some studies also show links between traffic-related air pollution and lung cancer or respiratory mortality[6]. It has been suggested that metal components of particulate matter may in part be responsible for toxic effects of air pollution on the cardiovascular and respiratory system[7].

In the Transport related Air Pollution and Health impacts – Integrated Methodologies for Assessing Particulate Matter (TRANSPHORM) study, copper zinc and iron content of particulate matter were found to be associated with increases in inflammatory markers in the blood[8], which might be expected to be associated with increased risks of cardiovascular and other diseases. However, a separate TRANSPHORM study[9] analysis of 19 cohorts with 9,545 CVD deaths, did not find any associations with metal (or other) particulate components. Here we use the same datasets examine associations with mortality using a much larger dataset to estimate particulate metal exposures for a population of 13.6 million living in and near London, England, with 108,478 CVD deaths and additionally 48,483 respiratory deaths and 24,849 incident cases of lung cancer.

For peer review only

## Methods

Our study region covered a 10,782 km$^2$ area around London and Oxford (Figure 1) in 1533 wards, an English Census area classification (primary unit of the English electoral geography) with a mean surface area ~7.0 km$^2$ and average 8,892 inhabitants in our study period.

### Exposure data

In the region of London and Oxford particulate matter was monitored during the years 2010-2011 as part of the European Study of Cohorts and Air Pollution Effects (ESCAPE) project[10 11]. Filters from the ESCAPE project were analyzed for elemental composition and de Hoogh et al. (2013)[12] developed land use regression (LUR) models for a number of the elemental components including metals as part of the TRANSPHORM project. In brief, twenty sites were monitored for three 2-week periods[10]and $PM_{2.5}$ and $PM_{10}$ were separately collected using Harvard impactors. Their elemental composition was analyzed using energy dispersive X ray fluorescence. The association of PM elemental components with land use covariates relative to traffic, population, industry or nature was evaluated with LUR models. Then, local estimates at the postcode level were predicted and aggregated at the Super Output Area (SOA) level, with a population-weighted mean, for all SOAs in the study.
In the analyses, we used copper (Cu), iron (Fe) and zinc (Zn) in the $PM_{10}$ fraction and copper and iron in the $PM_{2.5}$ fraction, all linked to non-tailpipe emissions. LUR models for this selection of elements showed a good leave-one-out validation, explaining more than 77% ($R^2$) of the observed variability.

### Confounder data – deprivation, ethnicity and smoking data

To adjust for possible confounders in this study, we included area-level ethnicity from Census 2011 and accounted for percent of White and Asian people per ward as covariates in the models. We also used the 2007 Index of Multiple Deprivation (IMD) as a relative measure of area-level deprivation (publicly available from the Department for Communities and Local Government data.gov.uk). This combines seven domains; 'income', 'employment', 'education', 'barriers to housing and services', 'crime', 'health' and 'living environment'. The latter is divided into two subdomains: 'indoor' measuring the quality of housing and 'outdoor' linked to air quality and road traffic accidents [13]. We excluded from the study the 'health' and 'outdoor living environment' domains[14], since we examined associations between health outcomes and air

pollution measures. The remaining domains were linearly combined to generate a 'modified IMD' relative score used in the analysis. High values of the modified IMD indicate higher deprivation. As a proxy for smoking, we used ward level tobacco expenditure (pounds/week/inhabitant) data obtained from CACI (CACI tobacco expenditure data is © Copyright 1996-2014 CACI Limited).

**Health data**

Mortality counts for cardiovascular (CDC10 I00-I99) and respiratory (CDC10 J00-J99) disease and lung cancer incidence counts (C33 and C34 ICD10 codes) were extracted for 2008-2011 from Office National Statistics data held by the Small Area Health Statistics Unit (SAHSU), which provide 100% coverage of deaths. The counts were then adjusted by sex and 5-year age band.

**Statistical analysis**

The effect of PM exposure to copper, iron and zinc on health outcomes were analyzed with Poisson regression (a generalized linear model) of count data at small area (ward) level, implemented in a Bayesian framework with spatial residuals as follows:

Let $Y_i$ denote the number of cases recorded in the spatial unit $i$ and $E_i$ the expected count taking into account the age and sex structure of the population at risk (internal standardization). Then, using Poisson regression, $Y_i$ is assumed to follow a Poisson distribution with mean equal to $E_i RR_i$ such that

$$\log(RR_i) = \mu + \sum_{j=1}^{p_1} \alpha_j Confound_{ij} + \sum_{k=1}^{p_2} \beta_k PM_{ik} + U_i.$$

Here, $Confound_{ij}$ denotes the value of the confounder $j\,(1, \ldots, p_1)$ for area $i(1, \ldots, n)$, similarly $PM_{ik}$ stands for the PM $k(1, \ldots, p_2)$exposures, $U_i$ is a spatial random effect, modelled with an intrinsic conditional autoregressive model [15], accounting for the spatial dependence of residuals. For each health outcome, the analysis was performed separately for elemental constituents of in $PM_{10}$ and $PM_{2.5}$. This model is inferred using the Bayesian approach in R-package INLA[16]. We used the non-informative priors proposed as default in R-INLA and standardized confounders.

Regression parameters are expressed per Interdecile range (IDR) relative risk, i.e. the increase of the relative risk when the level of covariates increases from the 10$^{th}$ to 90$^{th}$ centile; the posterior mean and 95% credible bounds are given.

**Results**

There were 108,478 cardiovascular and 48,483 respiratory deaths and 24,849 incident lung cancer cases in the study area for 2008-11 (Table 1). Maps of the spatial distribution of the covariates and elemental concentrations show that highest values were in Greater London Area, with iron and zinc also high in areas with motorways (Figure 2). The percentage population ethnicity for wards had a median of 77% white and 9% Asian ethnicity. Most of the areas with low percentage of White population was concentrated in Greater London, which also had higher percentage of Asian (supplementary material figure S1).

**Table 1**: Descriptive statistics of health outcomes, modelled particulate metal concentrations, deprivation score, and ethnicity covariates for the 1533 wards in the study area in 2008-11.



For peer review only - http://bmjopen.bmj.com/site/about/guidelines.xhtml

| | 10th centile | mean | median | 90th centile | LOOCV $R^2$ (for LUR) |
|---|---|---|---|---|---|
| **Health outcomes** | Rates of health outcome (number of cases per hundred thousand people) | | | | |
| Cardiovascular mortality | 117.50 | 215.97 | 203.20 | 327.87 | |
| Respiratory mortality | 42.85 | 96.34 | 87.85 | 160.41 | |
| Lung cancer incidence | 25.06 | 48.44 | 45.75 | 75.86 | |
| **Modelled metal concentrations using LUR** | Metals in ng/$m^3$ | | | | |
| Cu $PM_{10}$ | 7.0 | 13.3 | 13.1 | 19.8 | 0.95 |
| Fe $PM_{10}$ | 223.2 | 378.9 | 357.0 | 596.7 | 0.95 |
| Zn $PM_{10}$ | 113.5 | 135.2 | 139.5 | 153.0 | 0.77 |
| Cu PM2.5 | 2.6 | 4.3 | 4.6 | 5.7 | 0.79 |
| Fe PM2.5 | 51.6 | 86.8 | 82.8 | 129.0 | 0.92 |
| **Area-level confounders** | | | | | |
| Deprivation (modified IMD) | 3.45 | 7.08 | 6.47 | 11.78 | |
| % of Asian | 2 | 13 | 9 | 33 | |
| % of White | 38 | 72 | 77 | 95 | |
| Tobacco expenditure (pounds/week/inhabitant) | 3.40 | 4.61 | 4.48 | 6.03 | |

The individual effect of each elemental constitute of particulate matter evaluated with the Poisson regression is displayed in Table 2. Statistically significant associations with PM metal concentrations were identified for cardiovascular and respiratory mortality but not lung cancer incidence. For cardiovascular mortality, copper in the $PM_{2.5}$ fraction was associated with a small increased risk RR 1.005 (95%CI 1.001, 1.009) per interdecile range (IDR) but iron had an apparent protective association (RR 0.042 95%CI 0.002, 0.995) albeit with extremely high uncertainty. For respiratory mortality, the copper in the $PM_{10}$ fraction had a very small protective association (RR 0.988 95%CI 0.978, 0.998), but $PM_{10}$ zinc was associated with an increased mortality risk (RR 1.136 95%CI 1.010, 1.277).



For peer review only - http://bmjopen.bmj.com/site/about/guidelines.xhtml

**Table 2**: Individual effects of metals, estimated with Poisson regression, on cardiovascular mortality, respiratory mortality and lung cancer incidence adjusted for tobacco weekly expenditure, IMD and percentage of Asian and White population. Mean and lower and upper bounds of the credible intervals of the inter-decile relative risk (RR).

| | Metal | RR | 95% credible intervals |
|---|---|---|---|
| **Cardiovascular mortality** | Cu $PM_{10}$ | 0.994 | (0.987,1.001) |
| | Fe $PM_{10}$ | 0.319 | (0.037,2.779) |
| | Zn $PM_{10}$ | 1.073 | (0.985,1.169) |
| | Cu PM2.5 | 1.005 | (1.001,1.009) |
| | Fe PM2.5 | 0.042 | (0.002,0.995) |
| **Respiratory mortality** | Cu $PM_{10}$ | 0.988 | (0.978,0.998) |
| | Fe $PM_{10}$ | 0.649 | (0.033,12.767) |
| | Zn $PM_{10}$ | 1.136 | (1.010,1.277) |
| | Cu PM2.5 | 1.003 | (0.998,1.009) |
| | Fe PM2.5 | 0.980 | (0.013,72.673) |
| **Lung cancer incidence** | Cu $PM_{10}$ | 1.003 | (0.993,1.012) |
| | Fe $PM_{10}$ | 0.079 | (0.003,1.938) |
| | Zn $PM_{10}$ | 1.003 | (0.884,1.138) |
| | Cu PM2.5 | 0.997 | (0.991,1.003) |
| | Fe PM2.5 | 15.757 | (0.247,1004.125) |

For peer review only



For peer review only - http://bmjopen.bmj.com/site/about/guidelines.xhtml

The elements were highly correlated: 0.88 for $PM_{2.5}$ elements and 0.88-0.92 for $PM_{10}$ elements (Table 3). For $PM_{10}$ the correlation between copper and zinc was 0.85, and for $PM_{2.5}$ the correlation between copper and iron was 0.88. Thus, it is not possible to definitively attribute an association with one metal element given the inter-dependence.

**Table 3**: Correlation between the particle metals (PM).

| | $PM_{10}$ Copper | $PM_{10}$ Iron | $PM_{10}$ Zinc | $PM_{2.5}$ Iron | $PM_{2.5}$ Copper |
|---|---|---|---|---|---|
| **$PM_{10}$ Copper** | 1 | | | | |
| **$PM_{10}$ Iron** | 0.85 | 1 | | | |
| **$PM_{10}$ Zinc** | 0.85 | 0.92 | 1 | | |
| **$PM_{2.5}$ Iron** | 0.82 | 0.91 | 0.93 | 1 | |
| **$PM_{2.5}$ Copper** | 0.75 | 0.89 | 0.90 | 0.88 | 1 |

Area-level deprivation and weekly tobacco spend had a clear adverse association with cardiovascular mortality, respiratory mortality and lung cancer incidence (supplementary material Table S1). On the contrary, the proportions of White and Asian people in wards was associated with lower risks for the three diseases, suggesting a weak influence of the ethnic composition of the population on mortality/incidence rate.

## Discussion

This ecological study at small area level examined associations between modelled particulate metal (copper, iron and zinc) concentrations in relation to cardiovascular and respiratory mortality and lung cancer incidence in and around Greater London covering 13.6 million population with approximately 110,000 cardiorespiratory deaths and 25,000 new lung cancer cases. While the results did not find evidence of association between ambient particulate metal concentrations and lung cancer incidence, Poisson regression suggested copper in the $PM_{2.5}$ fraction was associated with increased cardiovascular mortality risk and $PM_{10}$ zinc with respiratory mortality risk. However, results for metal constituents were not fully consistent within our study.

Advantages of our study include the use of extremely large datasets with population coverage giving good statistical power to detect even very small associations. Another advantage was the use of standardized exposure models developed from standardized monitoring campaigns to estimate spatial variability in long-term exposures. While exposure data were derived from LUR models that showed good predictability, they may still misclassify true exposure. A limitation in our exposure assessment is the limited number of monitoring sites, 20, which potentially can lead to overfitting of the developed LUR models[17]. Providing that densities of measurement sites and estimation sites (wards) are similar, Szpiro and Paciorek (2013)[18] show that in case of over-smoothing of the exposure, the association between outcomes and exposure may be underestimated. In our case, over-smoothing likely occurs and this issue may partially explain our difficulty to show evidence of associations between health outcomes and exposures to particulate



For peer review only - http://bmjopen.bmj.com/site/about/guidelines.xhtml

elements. As most other ambient air pollution studies, we use outdoor concentration of pollutants at residence, without taking into account indoor levels, travel exposure or places of work. The correlation between indoor and outdoor concentration is high for fine particulate ($PM_{2.5}$)[19], suggesting that ignoring the indoor concentration is a small issue. However, in the London region, the difference of exposure at home and workplace may be different, since a part of the population living in suburban areas work in the city center, where exposures are higher. We used associations between mortality 2008-11 and particulate metals for 2010-11, which should also be representative of the preceding two years and should capture deaths related to short- and intermediate-long term influences. However, we used an ecological study design with limited ability to control for confounders at the individual level.

There are a limited number of other health studies looking at copper, zinc and iron metal components of particulates. Three studies looking at long-term effects using similarly derived estimates from the TRANSPHORM project as used here but much smaller numbers of health events than this study, found significant associations with inflammatory markers in blood but not health events. Hampel et al. [8] found statistically significant associations between $PM_{2.5}$ copper and $PM_{10}$ iron with high-sensitivity C-reactive protein and $PM_{2.5}$ zinc with fibrinogen in five European cohorts with available biomarkers (>17,000 measurements). Wolf et al [20] found elevated but non-significant associations with copper, zinc and iron constituents of particulates with incident coronary events in 11 cohorts (5,157 events), while Wang et al. [9] did not find long-term associations with cardiovascular mortality (9545 deaths) in 19 European cohorts where exposure results from a single year were applied over 2-20 years follow-up, in some cases retrospectively. A further study, the California teachers study [21] found associations between PM2.5 copper estimated in 2001-7 and contemporaneous ischemic heart disease deaths (1085 events) and elevated but non-significant associations with PM2.5 iron and other metals.

Short-term associations of metal components of particulates with mortality were examined in a systematic review of time series studies of fine-particle components and health published up to 2013 [22]. Zinc, indicative of road dust and possibly a result of tyre wear, was associated with daily mortality in eight of eleven studies included in the review. The subsequently published MED-PARTICLES time-series analysis in five European cities Basagaña et al. [17] found significant short-term associations with $PM_{10}$ copper iron and zinc and $PM_{2.5}$ iron with cardiovascular hospitalizations and $PM_{10}$ and $PM_{2.5}$ zinc for respiratory disease hospitalizations, but no significant associations were seen for mortality.

The reason that results for metal constituents of particulates are not completely consistent across studies, may be that metal concentrations serve as a proxy for oxidative potential[23]. Within the study area and in the analysis, the TRANSPHORM metal particulate measurements used to derive the land use regression models were highly correlated with oxidative potential of the particulates as measured using ascorbate (Pearson r = 0.93 for copper, 0.95 for iron, 0.67 for zinc)[23]. The high correlations between metal constituents of particulates raise the possibility that observed associations for one metal actually relate to another element that was better estimated. The high correlations also preclude conducting multi-pollutant analyses using Poisson regression.



For peer review only - http://bmjopen.bmj.com/site/about/guidelines.xhtml

## Conclusion

We found associations suggestive of small increased risk of cardiovascular and respiratory mortality but not lung cancer incidence in Greater London and surroundings in relation to metal concentrations of ambient particulate matter, which are likely derived from non-tailpipe road traffic emissions (brake and tyre-wear). Findings are consistent with a previous study finding associations of particulate metals with inflammatory markers, but further work is needed to better define exposures to airborne metal elements and non-tailpipe emissions.

## References

1. Pope CA, Burnett RT, Thurston GD, et al. Cardiovascular Mortality and Long-Term Exposure to Particulate Air Pollution. *Epidemiological Evidence of General Pathophysiological Pathways of Disease* 2004;109(1):71-77. doi: 10.1161/01.Cir.0000108927.80044.7f
2. Puett RC, Hart JE, Suh H, et al. Particulate Matter Exposures, Mortality, and Cardiovascular Disease in the Health Professionals Follow-up Study. *Environmental Health Perspectives* 2011;119(8):1130-35. doi: 10.1289/ehp.1002921
3. Zhang LW, Chen X, Xue XD, et al. Long-term exposure to high particulate matter pollution and cardiovascular mortality: a 12-year cohort study in four cities in northern China. *Environ Int* 2014;62:41-7. doi: 10.1016/j.envint.2013.09.012 [published Online First: 2013/10/29]
4. Brook RD, Rajagopalan S, Pope CA, et al. Particulate Matter Air Pollution and Cardiovascular Disease. *An Update to the Scientific Statement From the American Heart Association* 2010;121(21):2331-78. doi: 10.1161/CIR.0b013e3181dbece1
5. Crouse DL, Peters PA, van Donkelaar A, et al. Risk of nonaccidental and cardiovascular mortality in relation to long-term exposure to low concentrations of fine particulate matter: a Canadian national-level cohort study. *Environ Health Perspect* 2012;120(5):708-14. doi: 10.1289/ehp.1104049 [published Online First: 2012/02/09]
6. Beelen R, Hoek G, van den Brandt PA, et al. Long-Term Effects of Traffic-Related Air Pollution on Mortality in a Dutch Cohort (NLCS-AIR Study). *Environmental Health Perspectives* 2008;116(2):196-202. doi: 10.1289/ehp.10767
7. Li H, Qian X, Wang Qg. Heavy Metals in Atmospheric Particulate Matter: A Comprehensive Understanding Is Needed for Monitoring and Risk Mitigation. *Environmental Science & Technology* 2013;47(23):13210-11. doi: 10.1021/es404751a
8. Hampel R, Peters A, Beelen R, et al. Long-term effects of elemental composition of particulate matter on inflammatory blood markers in European cohorts. *Environment International* 2015;82:76-84. doi: https://doi.org/10.1016/j.envint.2015.05.008
9. Wang M, Beelen R, Stafoggia M, et al. Long-term exposure to elemental constituents of particulate matter and cardiovascular mortality in 19 European cohorts: Results from the ESCAPE and TRANSPHORM projects. *Environment International* 2014;66:97-106. doi: https://doi.org/10.1016/j.envint.2014.01.026
10. Eeftens M, Tsai M-Y, Ampe C, et al. Spatial variation of PM2.5, PM10, PM2.5 absorbance and PMcoarse concentrations between and within 20 European study areas and the relationship with NO2 -



For peer review only

Results of the ESCAPE project. *Atmospheric Environment* 2012;62:303-17. doi: 10.1016/j.atmosenv.2012.08.038

11. Tsai M-Y, Hoek G, Eeftens M, et al. Spatial variation of PM elemental composition between and within 20 European study areas — Results of the ESCAPE project. *Environment International* 2015;84:181-92. doi: https://doi.org/10.1016/j.envint.2015.04.015
12. de Hoogh K, Wang M, Adam M, et al. Development of Land Use Regression Models for Particle Composition in Twenty Study Areas in Europe. *Environmental Science & Technology* 2013;47(11):5778-86. doi: 10.1021/es400156t
13. deprivation Eio. https://www.gov.uk/government/statistics/english-indices-of-deprivation-2010, 2010.
14. Adams J, White M. Removing the health domain from the Index of Multiple Deprivation 2004—effect on measured inequalities in census measure of health. *Journal of Public Health* 2006;28(4):379-83. doi: 10.1093/pubmed/fdl061
15. Besag J, York J, Mollié A. Bayesian image restoration, with two applications in spatial statistics. *Annals of the Institute of Statistical Mathematics* 1991;43(1):1-20. doi: 10.1007/bf00116466
16. Martins TG, Simpson D, Lindgren F, et al. Bayesian computing with INLA:New features. *Computational Statistics & Data Analysis* 2013;67:68-83. doi: 10.1016/j.csda.2013.04.014
17. Basagaña X, Rivera M, Aguilera I, et al. Effect of the number of measurement sites on land use regression models in estimating local air pollution. *Atmospheric Environment* 2012;54:634-42. doi: https://doi.org/10.1016/j.atmosenv.2012.01.064
18. Szpiro AA, Paciorek CJ. Measurement error in two-stage analyses, with application to air pollution epidemiology. *Environmetrics* 2013;24(8):501-17. doi: 10.1002/env.2233
19. Brunekreef B, Holgate ST. Air pollution and health. *The Lancet* 2002;360(9341):1233-42. doi: https://doi.org/10.1016/S0140-6736(02)11274-8
20. Wolf K, Stafoggia M, Cesaroni G, et al. Long-term Exposure to Particulate Matter Constituents and the Incidence of Coronary Events in 11 European Cohorts. *Epidemiology* 2015;26(4):565-74. doi: 10.1097/ede.0000000000000300
21. Ostro B, Hu J, Goldberg D, et al. Associations of Mortality with Long-Term Exposures to Fine and Ultrafine Particles, Species and Sources: Results from the California Teachers Study Cohort. *Environmental Health Perspectives* 2015;123(6):549-56. doi: 10.1289/ehp.1408565
22. Atkinson RW, Analitis A, Samoli E, et al. Short-term exposure to traffic-related air pollution and daily mortality in London, UK. *Journal Of Exposure Science And Environmental Epidemiology* 2015;26:125. doi: 10.1038/jes.2015.65

https://www.nature.com/articles/jes201565#supplementary-information

23. Gulliver J, Morley D, Dunster C, et al. Land use regression models for the oxidative potential of fine particles (PM2.5) in five European areas. *Environmental Research* 2018;160:247-55. doi: https://doi.org/10.1016/j.envres.2017.10.002



For peer review only - http://bmjopen.bmj.com/site/about/guidelines.xhtml

Figures

Figure 1. Study Area compromising London and Oxford areas, with major roads and motorways. In the inset the area localization with regard to England map.

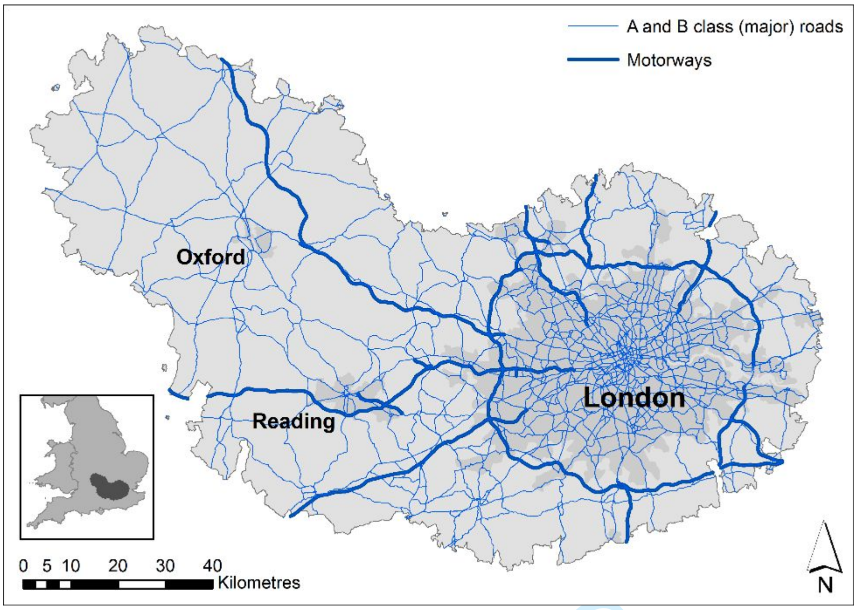


For peer review only

Figure 2. Maps of the metal exposures population weighted by ward.

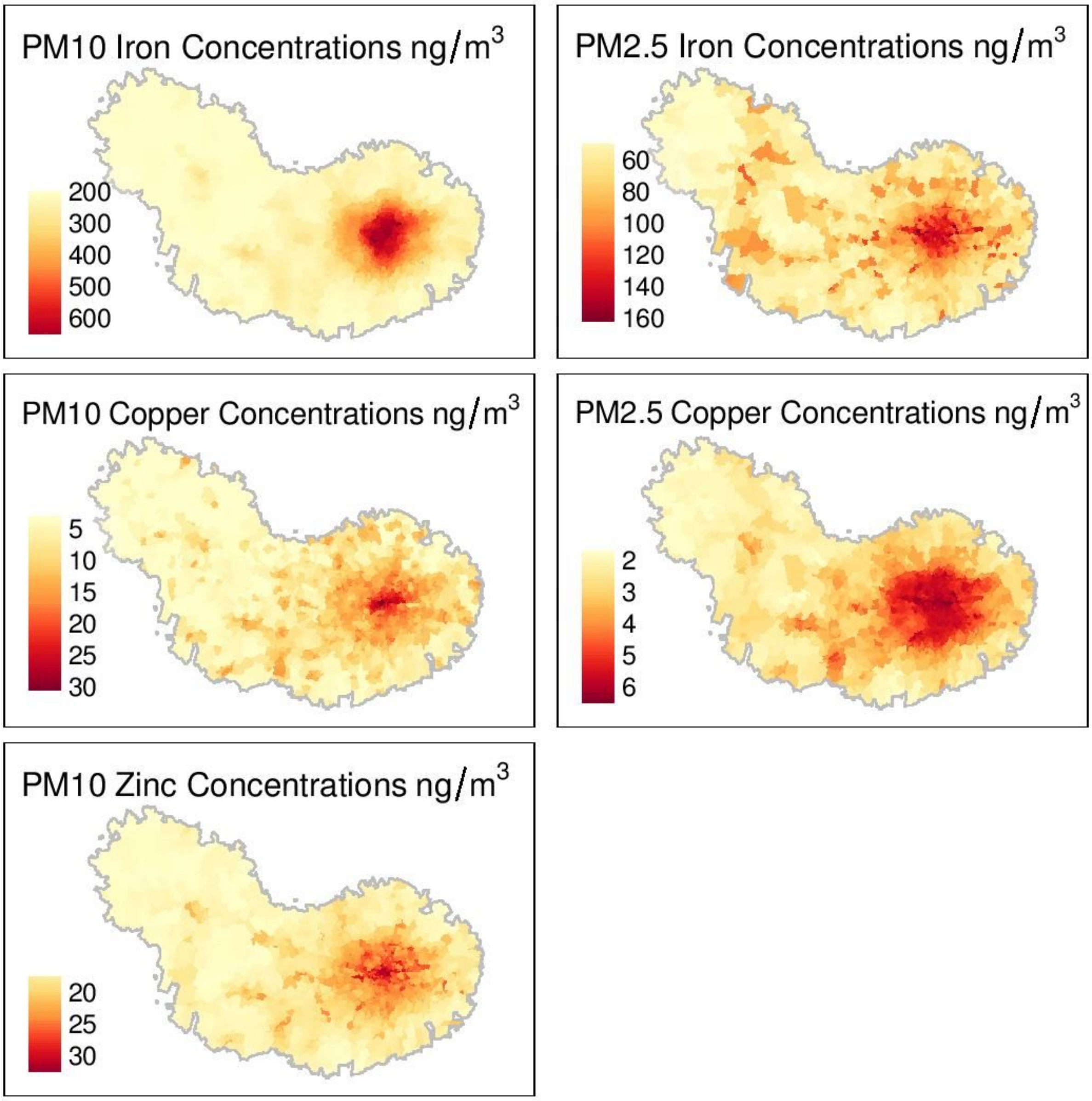

Supplementary material to:

**Associations between metal constituents of ambient particulate matter and mortality in England; a small area study**

**Table S1:** Poisson regression confounder effects from the two models (i) using metals from $PM_{10}$ and (ii) metals from $PM_{2.5}$ for all the health outcomes. Mean, lower and upper bound of the 95% credible interval of the inter-decile relative risk (RR).

| Outcomes | Model | Confounders | RR | CI 95% |
|---|---|---|---|---|
| Cardiovascular mortality | All Metals in $PM_{10}$ | IMD | 1.098 | (1.02,1.182) |
| | | % Asian | 0.982 | (0.921,1.046) |
| | | % White | 0.817 | (0.729,0.915) |
| | | Tobacco expenditure | 1.197 | (1.133,1.265) |
| | All Metals in $PM_{2.5}$ | IMD | 1.095 | (1.02,1.177) |
| | | % Asian | 0.987 | (0.926,1.052) |
| | | % White | 0.824 | (0.737,0.922) |
| | | Tobacco expenditure | 1.192 | (1.135,1.253) |
| Respiratory mortality | All Metals in $PM_{10}$ | IMD | 1.188 | (1.073,1.315) |
| | | % Asian | 0.887 | (0.813,0.967) |
| | | % White | 0.822 | (0.704,0.959) |
| | | Tobacco expenditure | 1.301 | (1.206,1.403) |
| | All Metals in $PM_{2.5}$ | IMD | 1.183 | (1.07,1.306) |
| | | % Asian | 0.892 | (0.817,0.973) |
| | | % White | 0.846 | (0.725,0.986) |
| | | Tobacco expenditure | 1.301 | (1.214,1.393) |
| Lung cancer incidence | All Metals in $PM_{10}$ | IMD | 1.432 | (1.284,1.596) |
| | | % Asian | 0.824 | (0.756,0.898) |
| | | % White | 0.804 | (0.691,0.936) |
| | | Tobacco expenditure | 1.401 | (1.29,1.522) |
| | All Metals in $PM_{2.5}$ | IMD | 1.465 | (1.316,1.63) |
| | | % Asian | 0.815 | (0.748,0.888) |
| | | % White | 0.817 | (0.702,0.949) |
| | | Tobacco expenditure | 1.357 | (1.259,1.462) |

For peer review only

**Figure S1.** Maps of the confounders in quintiles: proportion of Asian people, proportion of white people, modified index of multiple deprivations, and tobacco spends.

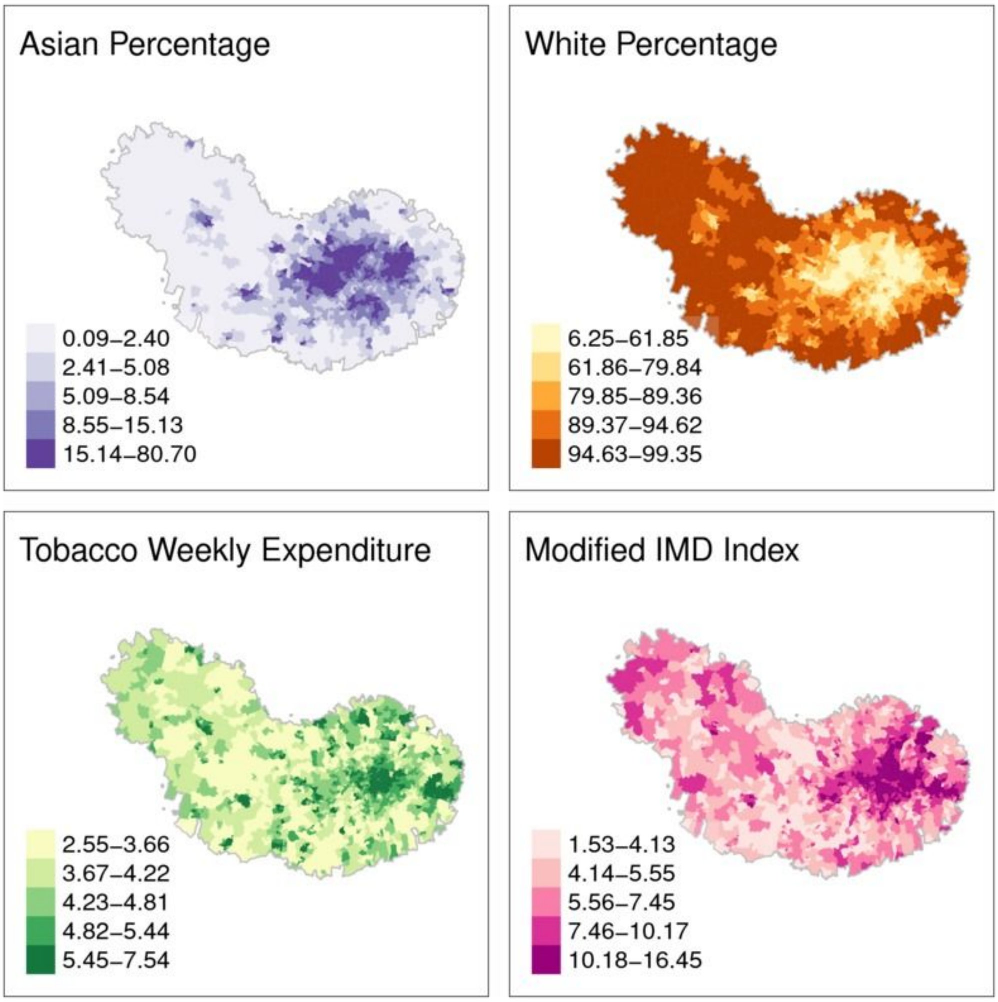

**STROBE 2007 (v4) Statement—Checklist of items that should be included in reports of *cross-sectional studies***

| **Section/Topic** | **Item #** | **Recommendation** | **Reported on page #** |
|---|---|---|---|
| **Title and abstract** | 1 | (*a*) Indicate the study's design with a commonly used term in the title or the abstract | 1 |
| | | (*b*) Provide in the abstract an informative and balanced summary of what was done and what was found | 3 |
| **Introduction** | | | |
| Background/rationale | 2 | Explain the scientific background and rationale for the investigation being reported | 5 |
| Objectives | 3 | State specific objectives, including any prespecified hypotheses | 5 |
| **Methods** | | | |
| Study design | 4 | Present key elements of study design early in the paper | 5 |
| Setting | 5 | Describe the setting, locations, and relevant dates, including periods of recruitment, exposure, follow-up, and data collection | 5-6 |
| Participants | 6 | (*a*) Give the eligibility criteria, and the sources and methods of selection of participants | 5-6, Figure 1 |
| Variables | 7 | Clearly define all outcomes, exposures, predictors, potential confounders, and effect modifiers. Give diagnostic criteria, if applicable | 5-6, Figure 2 and supplementary Figure S1 |
| Data sources/ measurement | 8* | For each variable of interest, give sources of data and details of methods of assessment (measurement). Describe comparability of assessment methods if there is more than one group | 5-6 |

For peer review only

| | | | |
|---|---|---|---|
| Bias | 9 | Describe any efforts to address potential sources of bias | 5, 7 |
| Study size | 10 | Explain how the study size was arrived at | Figure 1 |
| Quantitative variables | 11 | Explain how quantitative variables were handled in the analyses. If applicable, describe which groupings were chosen and why | 5-6 |
| Statistical methods | 12 | (*a*) Describe all statistical methods, including those used to control for confounding | 6 |
| | | (*b*) Describe any methods used to examine subgroups and interactions | |
| | | (*c*) Explain how missing data were addressed | No missing data! |
| | | (*d*) If applicable, describe analytical methods taking account of sampling strategy | NA – used all deaths, all population in area |
| | | (*e*) Describe any sensitivity analyses | None conducted |
| **Results** | | | |
| Participants | 13* | (a) Report numbers of individuals at each stage of study—eg numbers potentially eligible, examined for eligibility, confirmed eligible, included in the study, completing follow-up, and analysed | Table 1 and table 2 |
| | | (b) Give reasons for non-participation at each stage | NA |
| | | (c) Consider use of a flow diagram | NA |
| Descriptive data | 14* | (a) Give characteristics of study participants (eg demographic, clinical, social) and information on exposures and potential confounders | Supplementary Table S1 and Figure S1, 6 |
| | | (b) Indicate number of participants with missing data for each variable of interest | NA |
| Outcome data | 15* | Report numbers of outcome events or summary measures | 6 |

For peer review only

| | | | |
|---|---|---|---|
| Main results | 16 | (*a*) Give unadjusted estimates and, if applicable, confounder-adjusted estimates and their precision (eg, 95% confidence interval). Make clear which confounders were adjusted for and why they were included | 9-10, Table 1 and 3, Supplementary table S1 |
| | | (*b*) Report category boundaries when continuous variables were categorized | NA (no categorization) |
| | | (*c*) If relevant, consider translating estimates of relative risk into absolute risk for a meaningful time period | NA |
| Other analyses | 17 | Report other analyses done—eg analyses of subgroups and interactions, and sensitivity analyses | NA |
| **Discussion** | | | |
| Key results | 18 | Summarise key results with reference to study objectives | 7 |
| Limitations | 19 | Discuss limitations of the study, taking into account sources of potential bias or imprecision. Discuss both direction and magnitude of any potential bias | 8 |
| Interpretation | 20 | Give a cautious overall interpretation of results considering objectives, limitations, multiplicity of analyses, results from similar studies, and other relevant evidence | 8 |
| Generalisability | 21 | Discuss the generalisability (external validity) of the study results | 8 |
| **Other information** | | | |
| Funding | 22 | Give the source of funding and the role of the funders for the present study and, if applicable, for the original study on which the present article is based | 2 |

*Give information separately for cases and controls in case-control studies and, if applicable, for exposed and unexposed groups in cohort and cross-sectional studies.

For peer review only

**Note:** An Explanation and Elaboration article discusses each checklist item and gives methodological background and published examples of transparent reporting. The STROBE checklist is best used in conjunction with this article (freely available on the Web sites of PLoS Medicine at http://www.plosmedicine.org/, Annals of Internal Medicine at http://www.annals.org/, and Epidemiology at http://www.epidem.com/). Information on the STROBE Initiative is available at www.strobe-statement.org.

For peer review only

BMJ Open

# Associations between metal constituents of ambient particulate matter and mortality in England; an ecological study


| | |
|---|---|
| Journal: | *BMJ Open* |
| Manuscript ID | bmjopen-2019-030140.R1 |
| Article Type: | Original research |
| Date Submitted by the Author: | 18-Jul-2019 |


| | |
|---|---|
| Complete List of Authors: | Lavigne, Aurore; Université de Lille 3 UFR MIME, UFR MIME, Domaine universitaire du Pont de Bois<br>Freni Sterrantino , Anna ; Imperial College London, Epidemiology and Biostatistics<br>Liverani, Silvia; Queen Mary University of London, School of Mathematical Sciences<br>Blangiardo, Marta; Imperial College London, MRC-PHE Centre for Environment and Health, Department of Epidemiology and Biostatistics, School of Public Health<br>de Hoogh, Kees; Swiss Tropical and Public Health Institute; University of Basel<br>Molitor, John ; Oregon State University CAPS, School of Biological and Population Health Sciences, College of Public Health and Human Sciences<br>Hansell, Anna ; University of Leicester, |


| | |
|---|---|
| <b>Primary Subject Heading</b>: | Epidemiology |
| Secondary Subject Heading: | Health policy |
| Keywords: | Particulate matter elements, multipollutant effect, correlation, Metals, Environmental Epidemiology, EPIDEMIOLOGY |
| | |


SCHOLARONE™
Manuscripts

**Research Artic**

# Associations between metal constituents of ambient particulate matter and mortality in England; an ecological study

Aurore Lavigne*, Université Lille 3, UFR MIME, Domaine Universitaire du Pont de Bois, Villeneuve d'ascq Cedex, France

Anna Freni-Sterrantino*, Small Area Health Statistics Unit, Imperial College London, United Kingdom

Silvia Liverani, School of Mathematical Science, Queen Mary University of London, United Kingdom

Marta Blangiardo, Department of Epidemiology and Biostatistics, Imperial College London, United Kingdom

Kees de Hoogh, Swiss Tropical and Public Health Institute, Basel, Switzerland

John Molitor, School of Biological and Population Health Sciences, College of Public Health and Human Sciences, Oregon State University, USA

Anna L. Hansell, Centre for Environmental Health and Sustainability, George Davies Centre, Dept of Health Sciences, University of Leicester, United Kingdom

Small Area Health Statistics Unit, Imperial College London, United Kingdom

**Corresponding author: Anna L. Hansell,**

Centre for Environmental Health and Sustainability, George Davies Centre, Dept of Health Sciences, University of Leicester, United Kingdom 15 Lancaster Rd, Leicester LE1 7HA
Email: ah618@leicester.ac.uk
* joint first authors

The authors report no conflict of interest

**Availability of computing code and data:** The mortality cancer and population data used in this article were supplied by the Office for National Statistics (ONS), derived from the national mortality, cancer and birth registrations and the Census. SAHSU does not have permission to supply data to third parties, but the health and population data can be obtained from ONS on application. Air pollution estimates by ward for 2008-11 for the study area and code used can be obtained on request from the authors.
No identifiable information will be shared with any other organization. The scripts used can be provided by request from the authors.

**Acknowledgements**

We would like to acknowledge and thank Prof. John Gulliver, Dr Gary Fuller, Dr David Morley and Prof. Nicky Best for their useful comments.
CACI tobacco expenditure data is © Copyright 1996-2014 CACI Limited.

**Ethics and Information Governance statement**

SAHSU holds approvals from the National Research Ethics Service - reference 12/LO/0566 and 12/LO/0567 - and from the Health Research Authority Confidentially Advisory Group (HRA-CAG) for Section 251 support (HRA - 14/CAG/1039) for use of the health data used in this research.



For peer review only - http://bmjopen.bmj.com/site/about/guidelines.xhtml

The research was funded/part funded by the National Institute for Health Research Health Protection Research Unit (NIHR HPRU) in Health Impact of Environmental Hazards at King's College London in partnership with Public Health England (PHE) and Imperial College London. The views expressed are those of the author(s) and not necessarily those of the NHS, the NIHR, the Department of Health & Social Care or Public Health England".

**Funding**

The research project was funded through Medical Research Council (grant G09018401) and the Small Area Health Statistics Unit. The work of the UK Small Area Health Statistics Unit is funded by Public Health England as part of the MRC-PHE Centre for Environment and Health, funded also by the UK Medical Research Council.
The air pollution exposure assessments used in the research leading to these results was funded by the European Community's Seventh Framework Program (FP7/2007-2011) projects ESCAPE (grant agreement 211250) and TRANSPHORM (ENV.2009.1.2.2.1).

**Author's Contribution**

AL and AFS drafted the paper and ran the statistical analyses. KH provided exposure data. SL, JM and MB advised on the statistical methods. AH designed he study. All the authors provided intellectual input, interpreted the results, and helped to revise the manuscript. All authors approved the final version of the manuscript and agreed to be accountable for all the aspects of the work in ensuring that questions related to the accuracy or integrity of any part of the work are appropriately investigated and resolved. AH is the guarantor of this paper.

**Word Count: 2198**

**Figures: 2**

**Tables: 3**

For peer review only

**Abstract**

**Objectives** To investigate long-term associations between metal components of particulate matter and mortality and lung cancer incidence

**Design** Small area (ecological) study

**Setting** Population living in all wards (~9000 individuals per ward) in the London and Oxford area of England, comprising 13.6 million individuals

**Exposure and Outcome measures** We used land use regression (LUR) models originally used in the Transport related Air Pollution and Health impacts – Integrated Methodologies for Assessing Particulate Matter (TRANSPHORM) study to estimate exposure to copper, iron and zinc in ambient air particulate matter. We examined associations of metal exposure with Office for National Statistics mortality data from cardiovascular (CVD) and respiratory causes and with lung cancer incidence in 2008-11.

**Results** There were 108,478 CVD deaths, 48,483 respiratory deaths and 24,849 incident cases of lung cancer in the study period and area. Using Poisson regression models adjusted for area-level deprivation, tobacco sales and ethnicity, we found associations between cardiovascular mortality and $PM_{2.5}$ copper with interdecile range (IDR-2.6-5.7 ng/m$^3$) and IDR Relative risk (RR) 1.005 (95%CI 1.001, 1.009) and between respiratory mortality and $PM_{10}$ zinc (IDR 1135-153 ng/m$^3$) and IDR RR 1.136 (95%CI 1.010, 1.277). We did not find relevant associations for lung cancer incidence. Metal elements were highly correlated.

**Conclusion** Our analysis showed small but not fully consistent adverse associations between mortality and particulate metal exposures likely derived from non-tailpipe road traffic emissions (brake and tyre-wear), which have previously been associated with increases in inflammatory markers in the blood.


**Keywords:** Particulate matter elements, multipollutant effect, correlation, Metals, Environmental Epidemiology

For peer review only



For peer review only - http://bmjopen.bmj.com/site/about/guidelines.xhtml

**Strengths and limitations of this study**

- One of the largest studies to explore exposure to metal components of ambient air in relation to mortality and lung cancer incidence, with 13.6 million population
- A large number of cases: 108,478 CVD deaths, 48,483 respiratory deaths and 24,849 incident cases of lung cancer in the study period and area, providing good statistical power to examine small excess risks
- Established exposure models, developed and evaluated with measurements from a standardised monitoring campaign
- An ecological study using registry data, without access to individual-level confounders other than age and sex
- Metals were very highly correlated so multi-pollutant models could not be used

For peer review only

## Introduction

Chronic exposure to toxic substances in fine particulate matter (PM) with aerodynamic diameter less than 10µm ($PM_{10}$)[1-3] and 2.5µm ($PM_{2.5}$)[4] is associated with increased mortality levels from cardiovascular disease[1 5]. Some studies also show links between this long term exposure to traffic-related air pollution and lung cancer or respiratory mortality[6]. It has been suggested that metal components of particulate matter may in part be responsible for toxic effects of air pollution on the cardiovascular and respiratory system[7].

In the Transport related Air Pollution and Health impacts – Integrated Methodologies for Assessing Particulate Matter (TRANSPHORM) study, copper zinc and iron content of particulate matter were found to be associated -positively and significantly- with increases in inflammatory markers in the blood[8], which might be expected to be associated with increased risks of cardiovascular and other diseases. However, a separate TRANSPHORM study[9] analysis of 19 cohorts with 9,545 CVD deaths, did not find any statistically significant associations with metal (or other) particulate components. Here we use the same datasets to examine associations with mortality using a much larger dataset than TRANSPHORM study[9], to estimate particulate metal exposures for a population of 13.6 million living in and near London, England, with 108,478 CVD deaths and additionally 48,483 respiratory deaths and 24,849 incident cases of lung cancer.

## Methods

Our study region covered a 10,782 km$^2$ area around London and Oxford (Figure 1) in 1533 wards, an English Census area classification (primary unit of the English electoral geography) with a mean surface area ~7.0 km$^2$ and average 8,892 inhabitants per ward, in our study period.

### Exposure data

In the region of London and Oxford particulate matter was monitored during the years 2010-2011 as part of the European Study of Cohorts and Air Pollution Effects (ESCAPE) project[10 11]. Filters from the ESCAPE project were analyzed for elemental composition and de Hoogh et al. (2013)[12] developed land use regression (LUR) models for a number of the elemental components including metals as part of the TRANSPHORM project. In brief, twenty sites were monitored for three 2-week periods[10] and $PM_{2.5}$ and $PM_{10}$ were separately collected using Harvard impactors. Their elemental composition was analyzed using energy dispersive X ray fluorescence. The association of PM elemental components with land use covariates relative to traffic, population, industry or nature was evaluated with LUR models. Then, local estimates at the postcode level were predicted and aggregated at the Super Output Area (SOA) level, with a population-weighted mean, for all SOAs in the study. Exposure was assigned for each case or incidence at post-code level.
In the analyses, we used copper (Cu), iron (Fe) and zinc (Zn) in the $PM_{10}$ fraction and copper and iron in the $PM_{2.5}$ fraction, all linked to non-tailpipe emissions[12]. LUR models for this selection of elements showed a good leave-one-out validation, explaining more than 77% ($R^2$) of the observed variability.

### Confounder data – deprivation, ethnicity and smoking data

To adjust for possible confounders in this study, we included area-level ethnicity from Census 2011 and accounted for percent of White and Asian people per ward as covariates in the models. We also used the 2007 Index of Multiple Deprivation (IMD) as a relative measure of area-level deprivation (publicly available from the Department for Communities and Local Government data.gov.uk). This combines seven domains; 'income', 'employment', 'education', 'barriers to housing and services', 'crime', 'health' and 'living environment'. The latter is divided into two subdomains: 'indoor' measuring the quality of housing and 'outdoor' linked to air quality and road traffic accidents [13]. We excluded from the study the 'health' and



For peer review only - http://bmjopen.bmj.com/site/about/guidelines.xhtml

'outdoor living environment' domains[14], since we examined associations between health outcomes and air pollution measures. The remaining domains were linearly combined to generate a 'modified IMD' relative score used in the analysis. High values of the modified IMD indicate higher deprivation. As a proxy for smoking, we used ward level tobacco expenditure (pounds/week/inhabitant) data obtained from CACI (CACI tobacco expenditure data is © Copyright 1996-2014 CACI Limited).

**Health data**

Mortality counts for cardiovascular (CDC10 I00-I99) and respiratory (CDC10 J00-J99) disease and lung cancer incidence counts (C33 and C34 ICD10 codes) were extracted for 2008-2011 from Office National Statistics data held by the Small Area Health Statistics Unit (SAHSU), which provide 100% coverage of deaths. The counts were then adjusted by sex and 5-year age band.

**Patient and Public Involvement.**

Patients were not involved in the development of the research question or the design and conducting of the study.

**Statistical analysis**

The effect of PM exposure to copper, iron and zinc on health outcomes were analyzed with Poisson regression (a generalized linear model) of count data at small area (ward) level, implemented in a Bayesian framework with spatial residuals, see supplementary figure 1 for a graphical representation of the possible causal mechanism.

Let $Y_i$ denote the number of cases recorded in the spatial unit $i$ and $E_i$ the expected count taking into account the age and sex structure of the population at risk (internal standardization). Then, using Poisson regression, $Y_i$ is assumed to follow a Poisson distribution with mean equal to $E_i RR_i$ such that

$$\log(RR_i) = \mu + \sum_{j=1}^{p_1} \alpha_j Confound_{ij} + \beta PM_{ik} + U_i.$$

Here, $\mu$ is the model intercept, $Confound_{ij}$ denotes the value of the confounder $j\,(1, \ldots, p_1)$ for area $i(1, \ldots, n)$, similarly $PM_{ik}$ stands for the PM $k(1, \ldots, p_2)$exposures , $U_i$ is a spatial random effect, modelled with an intrinsic conditional autoregressive model [15], accounting for the spatial dependence of residuals. The coefficients $\alpha_j$ and $\beta$ indicate the linear effect of the confounders and PM-metals on the log relative risk.

For each health outcome, the analysis was performed separately for elemental constituents of $PM_{10}$ and $PM_{2.5}$. A second model was fitted, for each PM metal constituents and as measure of multicollinearity the and variation inflation factor (VIF) is provided.

Both models are inferred using the Bayesian approach in R-package INLA[16]. We used the non-informative priors proposed as default in R-INLA and standardized confounders.

Regression parameters are expressed per Interdecile range (IDR) relative risk, i.e. the increase of the relative risk when the level of covariates increases from the 10th to 90th centile; the posterior mean and 95% credible bounds are given.



For peer review only - http://bmjopen.bmj.com/site/about/guidelines.xhtml

**Results**

There were 108,478 cardiovascular and 48,483 respiratory deaths and 24,849 incident lung cancer cases in the study area for 2008-11 (Table 1). Maps of the spatial distribution of the covariates and elemental concentrations show that highest values were in Greater London Area, with iron and zinc also high in areas with motorways (Figure 2). The percentage population ethnicity for wards had a median of 77% white and 9% Asian ethnicity. Most of the areas with low percentage of White population was concentrated in Greater London, which also had higher percentage of Asian (supplementary material figure S1).

For peer review only



For peer review only - http://bmjopen.bmj.com/site/about/guidelines.xhtml

**Table 1**: Descriptive statistics of health outcomes, modelled particulate metal concentrations, deprivation score, and ethnicity covariates for the 1533 wards in the study area in 2008-11.

| | | Cu[1] $PM_{10}$ | | | | | | | | | | | |
|---|---|---|---|---|---|---|---|---|---|---|---|---|---|
| | | 10th centile (n=153) | | | | 10th-90th centile (n=1225) | | | | 90th centile (n=154) | | | |
| | | 10th centile | mean | median | 90th Centile | 10th centile | mean | median | 90th Centile | 10th centile | mean | median | 90th Centile |
| **Health outcomes** | | Standard Mortality/Incidence Ratio (ratio across whole study area =1.00) | | | | | | | | | | | |
| Cardiovascular mortality | | 0.6 | 0.87 | 0.85 | 1.19 | 0.71 | 1.01 | 0.99 | 1.32 | 0.62 | 1.02 | 1.04 | 1.34 |
| Respiratory mortality | | 0.36 | 0.81 | 0.75 | 1.28 | 0.62 | 1.02 | 0.98 | 1.45 | 0.49 | 0.98 | 1.01 | 1.4 |
| Lung cancer incidence | | 0.36 | 0.80 | 0.73 | 1.31 | 0.54 | 0.99 | 0.94 | 1.50 | 0.69 | 1.28 | 1.28 | 1.84 |
| **Modelled metal concentrations using LUR** | | Metals in ng/$m^3$ | | | | | | | | | | | |
| | **LOOCV * $R^2$ (for LUR**)** | | | | | | | | | | | | |
| Fe $PM_{10}$ | 0.95 | 200.94 | 212.81 | 208.38 | 227.67 | 220.04 | 315.33 | 267.54 | 483.68 | 543.93 | 604.61 | 614.69 | 647.44 |
| Zn $PM_{10}$ | 0.77 | 15.29 | 16.03 | 15.94 | 17 | 16.55 | 19.87 | 19.27 | 24.04 | 23.77 | 26.84 | 26.44 | 30.52 |
| Cu $PM_{2.5}$ | 0.79 | 1.83 | 2.3 | 2.18 | 2.91 | 2.5 | 3.84 | 3.65 | 5.41 | 5.32 | 5.71 | 5.72 | 6.1 |
| Fe $PM_{2.5}$ | 0.92 | 43.66 | 58.2 | 52.29 | 83.46 | 50.63 | 74.89 | 69.33 | 108.17 | 113.25 | 129.66 | 130.24 | 144.51 |
| **Area-level confounders** | | | | | | | | | | | | | |



For peer review only - http://bmjopen.bmj.com/site/about/guidelines.xhtml

| | | | | | | | | | | | | |
|---|---|---|---|---|---|---|---|---|---|---|---|---|
| IMD (modified Index of multiple deprivation) | 3.68 | 5.71 | 5.48 | 7.91 | 3.23 | 47.34 | 1.88 | 3.45 | 9.91 | 11.74 | 11.73 | 13.66 |
| % of White | 94.04 | 96.64 | 96.96 | 98.53 | 6.67 | 78.09 | 11.34 | 4.57 | 38.41 | 58.06 | 59.86 | 76.41 |
| % of Asian | 0.55 | 1.4 | 1.16 | 2.61 | 6.18 | 85.5 | 6.89 | 4.47 | 6.44 | 16.42 | 11.4 | 35.58 |
| Tobacco expenditure (pounds/week/inhabitant) | 3.12 | 3.72 | 3.67 | 4.35 | 10.8 | 95.56 | 25.14 | 5.91 | 5.01 | 5.84 | 5.9 | 6.59 |

*Leave one out cross-validation (LOOCV)
**Land Use Regression (LUR)
[1] Cu $PM_{10}$ LOOCV $R^2$ =0.95



For peer review only - http://bmjopen.bmj.com/site/about/guidelines.xhtml

The individual linear effect of each elemental constitute of particulate matter evaluated with the Poisson regression adjusted for confounders is displayed in Table 2 and Table S1 in Supplementary Material. Statistically significant associations with PM metal concentrations were identified for cardiovascular and respiratory mortality but not lung cancer incidence. For cardiovascular mortality, copper in the $PM_{2.5}$ fraction was associated with a small increased risk RR 1.005 (95%CI 1.001, 1.009) per interdecile range (IDR) but iron had an apparent protective association (RR 0.042 95%CI 0.002, 0.995) albeit with extremely high uncertainty. For respiratory mortality, the copper in the $PM_{10}$ fraction had a very small protective association (RR 0.988 95%CI 0.978, 0.998), but $PM_{10}$ zinc was associated with an increased mortality risk (RR 1.136 95%CI 1.010, 1.277).

**Table 2**: Individual effects of metals, estimated with Poisson regression, on cardiovascular mortality, respiratory mortality and lung cancer incidence adjusted for tobacco weekly expenditure, IMD (index of multiple deprivation) and percentage of Asian and White population. Mean and lower and upper bounds of the credible intervals of the inter-decile relative risk (RR).

| | **Metal** | **RR** | **95% credible intervals** |
|---|---|---|---|
| **Cardiovascular mortality** | Cu $PM_{10}$ | 0.994 | (0.987,1.001) |
| | Fe $PM_{10}$ | 0.319 | (0.037,2.779) |
| | Zn $PM_{10}$ | 1.073 | (0.985,1.169) |
| | Cu $PM_{2.5}$ | 1.005 | (1.001,1.009) |
| | Fe $PM_{2.5}$ | 0.042 | (0.002,0.995) |
| **Respiratory mortality** | Cu $PM_{10}$ | 0.988 | (0.978,0.998) |
| | Fe $PM_{10}$ | 0.649 | (0.033,12.767) |
| | Zn $PM_{10}$ | 1.136 | (1.010,1.277) |
| | Cu $PM_{2.5}$ | 1.003 | (0.998,1.009) |
| | Fe $PM_{2.5}$ | 0.980 | (0.013,72.673) |
| **Lung cancer incidence** | Cu $PM_{10}$ | 0.998 | (0.912,1.091) |
| | Fe $PM_{10}$ | 0.973 | (0.830,1.142) |
| | Zn $PM_{10}$ | 0.995 | (0.910,1.089) |
| | Cu $PM_{2.5}$ | 1.092 | (0.943,1.225) |
| | Fe $PM_{2.5}$ | 0.969 | (0.889,1.057) |

For peer review only



For peer review only - http://bmjopen.bmj.com/site/about/guidelines.xhtml

The elements were highly correlated: 0.88 for $PM_{2.5}$ elements and 0.82-0.92 for $PM_{10}$ elements (Table 3). For $PM_{10}$ the Pearson correlation between copper and zinc was 0.85, and for $PM_{2.5}$ the correlation between copper and iron was 0.88. The metal constituents showed high correlation with $PM_{2.5}$ and $PM_{10}$ mass concentrations for $PM_{2.5}$ and metals in $PM_{2.5}$ was 0.86-0.89 and 0.73-0.89 for $PM_{10}$ metals; for $PM_{10}$ and $PM_{10}$ metals 0.74-0.88 and 0.86-0.89 for metals in $PM_{2.5}$ (see supplementary table S2).
Thus, it is not possible to definitively attribute an association with one metal element given the inter-dependence.

**Table 3**: Pearson correlation between the particle metals (PM).

| | **$PM_{10}$ Copper** | **$PM_{10}$ Iron** | **$PM_{10}$ Zinc** | **$PM_{2.5}$ Iron** | **$PM_{2.5}$ Copper** |
|---|---|---|---|---|---|
| **$PM_{10}$ Copper** | 1 | | | | |
| **$PM_{10}$ Iron** | 0.85 | 1 | | | |
| **$PM_{10}$ Zinc** | 0.85 | 0.92 | 1 | | |
| **$PM_{2.5}$ Iron** | 0.82 | 0.91 | 0.93 | 1 | |
| **$PM_{2.5}$ Copper** | 0.75 | 0.89 | 0.90 | 0.88 | 1 |

In the model fit, for each group of metals by PM, we have found that area-level deprivation (IMD) and weekly tobacco spend had a clear adverse association with cardiovascular mortality, respiratory mortality and lung cancer incidence (supplementary material Table S2), with moderate high value of VIF. On the contrary, the proportions of White and Asian people in wards was associated with lower risks for the three diseases, suggesting a weak influence of the ethnic composition of the population on mortality/incidence rate.

## Discussion

This ecological study at small area level examined associations between modelled particulate metal (copper, iron and zinc) concentrations in relation to cardiovascular and respiratory mortality and lung cancer incidence in and around Greater London covering 13.6 million population with approximately 110,000 cardiorespiratory deaths and 25,000 new lung cancer cases. While the results did not find evidence of positive association between ambient particulate metal concentrations and lung cancer incidence, Poisson regression suggested copper in the $PM_{2.5}$ fraction was statistically significant associated with increased cardiovascular mortality risk and $PM_{10}$ zinc with respiratory mortality risk. Results for metal constituents were not fully consistent within our study for the same element in $PM_{2.5}$ and $PM_{10}$ size fractions. Metal exposures were highly correlated so it is difficult to definitively attribute an association with one metal element.

Advantages of our study include the use of extremely large datasets with population coverage giving good statistical power to detect even very small associations. Another advantage was the use of standardized exposure models developed from standardized monitoring campaigns to estimate spatial variability in long-term exposures. While exposure data were derived from LUR models that showed good predictability, they may still misclassify true exposure as (i) prediction is good but not perfect (ii) using a model of exposure at



For peer review only - http://bmjopen.bmj.com/site/about/guidelines.xhtml

residence as a proxy for personal exposure. A limitation in our exposure assessment is the limited number of monitoring sites, 20, which potentially can lead to overfitting of the developed LUR models[17]. Providing that densities of measurement sites and estimation sites (wards) are similar, Szpiro and Paciorek (2013)[18] show that in case of over-smoothing of the exposure, the association between outcomes and exposure may be underestimated. In our case, over-smoothing likely occurs and this issue may partially explain our difficulty to show evidence of adverse associations between health outcomes and exposures to particulate elements. As most other ambient air pollution studies, we use outdoor concentration of pollutants at residence, without taking into account indoor levels, travel exposure or places of work. The correlation between indoor and outdoor concentration is high for fine particulate ($PM_{2.5}$)[19], suggesting that ignoring the indoor concentration is a small issue. However, in the London region, the difference of exposure at home and workplace may be different, since a part of the population living in suburban areas work in the city center, where exposures are higher. We used associations between mortality 2008-11 and particulate metals for 2010-11, which should also be representative of the preceding two years and should capture deaths related to short- and intermediate-long term influences. However, we used an ecological study design with limited ability to control for confounders at the individual level.

There are a limited number of other health studies looking at copper, zinc and iron metal components of particulates. Three studies looking at long-term effects using similarly derived estimates from the TRANSPHORM project as used here but much smaller numbers of health events than this study, found significant associations with inflammatory markers in blood but not health events. Hampel et al. [8] found positive statistically significant associations between $PM_{2.5}$ copper and $PM_{10}$ iron with high-sensitivity C-reactive protein and $PM_{2.5}$ zinc with fibrinogen in five European cohorts with available biomarkers (>17,000 measurements). Wolf et al [20] found elevated but non-significant positive associations with copper, zinc and iron constituents of particulates with incident coronary events in 11 cohorts (5,157 events), while Wang et al. [9] did not find long-term positive associations with cardiovascular mortality (9545 deaths) in 19 European cohorts where exposure results from a single year were applied over 2-20 years follow-up, in some cases retrospectively. A further study, the California teachers study [21] found positive and significant associations between $PM_{2.5}$ copper estimated in 2001-7 and contemporaneous ischemic heart disease deaths (1085 events) and elevated but non-significant associations with $PM_{2.5}$ iron and other metals.
We did not find associations with lung cancer incidence. While toxicological studies suggest that metals in airborne particulates are genotoxic[22], the reason we did not find an association even in our large sample size may be because our exposure measures relate to a similar time frame as the health outcome. Studies finding associations of particulates with lung cancer have typically considered 10 or more years follow-up[23].

Short-term associations of metal components of particulates with mortality were examined in a systematic review of time series studies of fine-particle components and health published up to 2013 [24]. Zinc, indicative of road dust and possibly a result of tyre wear, was associated with daily mortality in eight of eleven studies included in the review. The subsequently published MED-PARTICLES time-series analysis in five European cities Basagaña et al. [17] found positive significant short-term associations with $PM_{10}$ copper iron and zinc and $PM_{2.5}$ iron with cardiovascular hospitalizations and $PM_{10}$ and $PM_{2.5}$ zinc for respiratory disease hospitalizations, but no significant associations were seen for mortality.

The reason that results for metal constituents of particulates are not completely consistent across studies, may be that metal concentrations serve as a proxy for oxidative potential[25]. Within the study area and in



For peer review only - http://bmjopen.bmj.com/site/about/guidelines.xhtml

the analysis, the TRANSPHORM metal particulate measurements used to derive the land use regression models were highly correlated with oxidative potential of the particulates as measured using ascorbate (Pearson r = 0.93 for copper, 0.95 for iron, 0.67 for zinc)[25]. The high correlations between metal constituents of particulates raise the possibility that observed associations for one metal actually relate to another element that was better estimated. The high correlations also preclude conducting multi-pollutant analyses using Poisson regression.

## Conclusion

We found positive and significant associations suggestive of small increased risk of cardiovascular and respiratory mortality but not lung cancer incidence in Greater London and surroundings in relation to metal concentrations of ambient particulate matter, which are likely derived from non-tailpipe road traffic emissions (brake and tyre-wear). Findings are consistent with a previous study finding associations of particulate metals with inflammatory markers, but further work is needed to better define exposures to airborne metal elements and non-tailpipe emissions.

## References


1. Pope CA, Burnett RT, Thurston GD, et al. Cardiovascular Mortality and Long-Term Exposure to Particulate Air Pollution. *Epidemiological Evidence of General Pathophysiological Pathways of Disease* 2004;109(1):71-77. doi: 10.1161/01.Cir.0000108927.80044.7f
2. Puett RC, Hart JE, Suh H, et al. Particulate Matter Exposures, Mortality, and Cardiovascular Disease in the Health Professionals Follow-up Study. *Environmental Health Perspectives* 2011;119(8):1130-35. doi: 10.1289/ehp.1002921
3. Zhang LW, Chen X, Xue XD, et al. Long-term exposure to high particulate matter pollution and cardiovascular mortality: a 12-year cohort study in four cities in northern China. *Environ Int* 2014;62:41-7. doi: 10.1016/j.envint.2013.09.012 [published Online First: 2013/10/29]
4. Brook RD, Rajagopalan S, Pope CA, et al. Particulate Matter Air Pollution and Cardiovascular Disease. *An Update to the Scientific Statement From the American Heart Association* 2010;121(21):2331-78. doi: 10.1161/CIR.0b013e3181dbece1
5. Crouse DL, Peters PA, van Donkelaar A, et al. Risk of nonaccidental and cardiovascular mortality in relation to long-term exposure to low concentrations of fine particulate matter: a Canadian national-level cohort study. *Environ Health Perspect* 2012;120(5):708-14. doi: 10.1289/ehp.1104049 [published Online First: 2012/02/09]
6. Beelen R, Hoek G, van den Brandt PA, et al. Long-Term Effects of Traffic-Related Air Pollution on Mortality in a Dutch Cohort (NLCS-AIR Study). *Environmental Health Perspectives* 2008;116(2):196-202. doi: 10.1289/ehp.10767
7. Li H, Qian X, Wang Qg. Heavy Metals in Atmospheric Particulate Matter: A Comprehensive Understanding Is Needed for Monitoring and Risk Mitigation. *Environmental Science & Technology* 2013;47(23):13210-11. doi: 10.1021/es404751a




For peer review only - http://bmjopen.bmj.com/site/about/guidelines.xhtml

8. Hampel R, Peters A, Beelen R, et al. Long-term effects of elemental composition of particulate matter on inflammatory blood markers in European cohorts. *Environment International* 2015;82:76-84. doi: https://doi.org/10.1016/j.envint.2015.05.008
9. Wang M, Beelen R, Stafoggia M, et al. Long-term exposure to elemental constituents of particulate matter and cardiovascular mortality in 19 European cohorts: Results from the ESCAPE and TRANSPHORM projects. *Environment International* 2014;66:97-106. doi: https://doi.org/10.1016/j.envint.2014.01.026
10. Eeftens M, Tsai M-Y, Ampe C, et al. Spatial variation of PM2.5, PM10, PM2.5 absorbance and PMcoarse concentrations between and within 20 European study areas and the relationship with NO2 - Results of the ESCAPE project. *Atmospheric Environment* 2012;62:303-17. doi: 10.1016/j.atmosenv.2012.08.038
11. Tsai M-Y, Hoek G, Eeftens M, et al. Spatial variation of PM elemental composition between and within 20 European study areas — Results of the ESCAPE project. *Environment International* 2015;84:181-92. doi: https://doi.org/10.1016/j.envint.2015.04.015
12. de Hoogh K, Wang M, Adam M, et al. Development of Land Use Regression Models for Particle Composition in Twenty Study Areas in Europe. *Environmental Science & Technology* 2013;47(11):5778-86. doi: 10.1021/es400156t
13. deprivation Eio. https://www.gov.uk/government/statistics/english-indices-of-deprivation-2010, 2010.
14. Adams J, White M. Removing the health domain from the Index of Multiple Deprivation 2004—effect on measured inequalities in census measure of health. *Journal of Public Health* 2006;28(4):379-83. doi: 10.1093/pubmed/fdl061
15. Besag J, York J, Mollié A. Bayesian image restoration, with two applications in spatial statistics. *Annals of the Institute of Statistical Mathematics* 1991;43(1):1-20. doi: 10.1007/bf00116466
16. Martins TG, Simpson D, Lindgren F, et al. Bayesian computing with INLA:New features. *Computational Statistics & Data Analysis* 2013;67:68-83. doi: 10.1016/j.csda.2013.04.014
17. Basagaña X, Rivera M, Aguilera I, et al. Effect of the number of measurement sites on land use regression models in estimating local air pollution. *Atmospheric Environment* 2012;54:634-42. doi: https://doi.org/10.1016/j.atmosenv.2012.01.064
18. Szpiro AA, Paciorek CJ. Measurement error in two-stage analyses, with application to air pollution epidemiology. *Environmetrics* 2013;24(8):501-17. doi: 10.1002/env.2233
19. Brunekreef B, Holgate ST. Air pollution and health. *The Lancet* 2002;360(9341):1233-42. doi: https://doi.org/10.1016/S0140-6736(02)11274-8
20. Wolf K, Stafoggia M, Cesaroni G, et al. Long-term Exposure to Particulate Matter Constituents and the Incidence of Coronary Events in 11 European Cohorts. *Epidemiology* 2015;26(4):565-74. doi: 10.1097/ede.0000000000000300
21. Ostro B, Hu J, Goldberg D, et al. Associations of Mortality with Long-Term Exposures to Fine and Ultrafine Particles, Species and Sources: Results from the California Teachers Study Cohort. *Environmental Health Perspectives* 2015;123(6):549-56. doi: 10.1289/ehp.1408565
22. Bocchi C, Bazzini C, Fontana F, et al. Characterization of urban aerosol: Seasonal variation of genotoxicity of the water-soluble portion of PM2.5 and PM1. *Mutation Research/Genetic Toxicology and Environmental Mutagenesis* 2019;841:23-30. doi: https://doi.org/10.1016/j.mrgentox.2019.04.005
23. Raaschou-Nielsen O, Andersen ZJ, Beelen R, et al. Air pollution and lung cancer incidence in 17 European cohorts: prospective analyses from the European Study of Cohorts for Air Pollution Effects (ESCAPE). *The Lancet Oncology* 2013;14(9):813-22. doi: https://doi.org/10.1016/S1470-2045(13)70279-1



For peer review only - http://bmjopen.bmj.com/site/about/guidelines.xhtml

24. Atkinson RW, Analitis A, Samoli E, et al. Short-term exposure to traffic-related air pollution and daily mortality in London, UK. *Journal Of Exposure Science And Environmental Epidemiology* 2015;26:125. doi: 10.1038/jes.2015.65

https://www.nature.com/articles/jes201565#supplementary-information

25. Gulliver J, Morley D, Dunster C, et al. Land use regression models for the oxidative potential of fine particles (PM2.5) in five European areas. *Environmental Research* 2018;160:247-55. doi: https://doi.org/10.1016/j.envres.2017.10.002

For peer review only



For peer review only - http://bmjopen.bmj.com/site/about/guidelines.xhtml

**Figures**

**Figure 1.** Study Area compromising London and Oxford areas, with major roads and motorways. In the inset the area localization with regard to England map. Contains National Statistics and Ordnance Survey data © Crown copyright and database right 2013.

**Figure 2.** Maps of the metal exposures population weighted by ward. Contains National Statistics and Ordnance Survey data © Crown copyright and database right 2013.

For peer review only



For peer review only - http://bmjopen.bmj.com/site/about/guidelines.xhtml

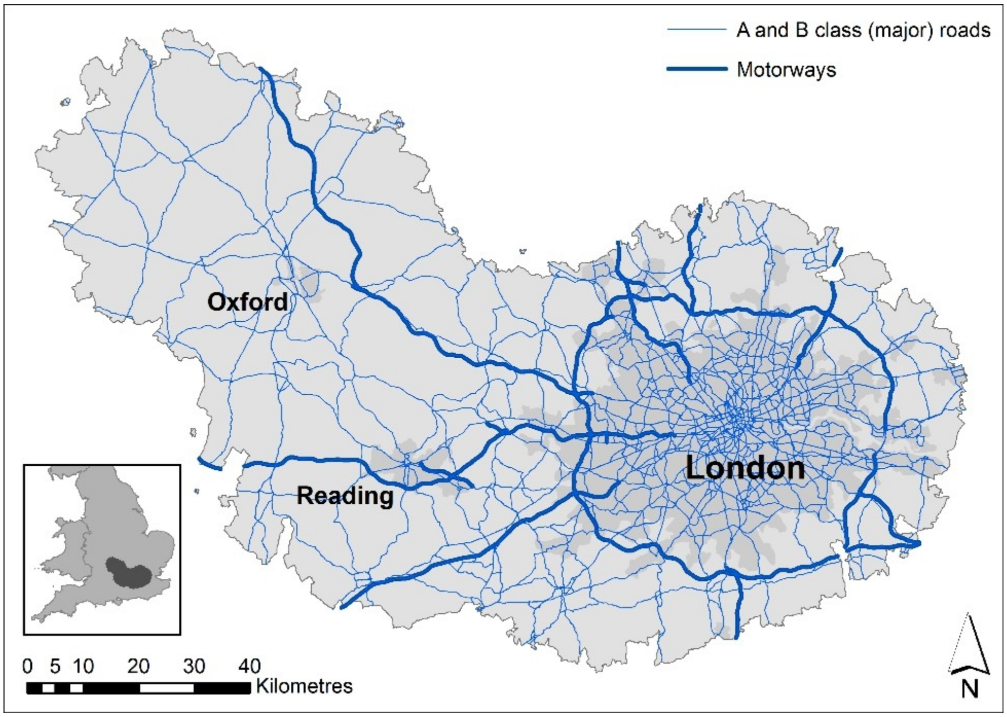


Study Area compromising London and Oxford areas, with major roads and motorways. In the inset the area localization with regard to England map. Contains National Statistics and Ordnance Survey data © Crown copyright and database right 2013.

112x79mm (220 x 220 DPI)

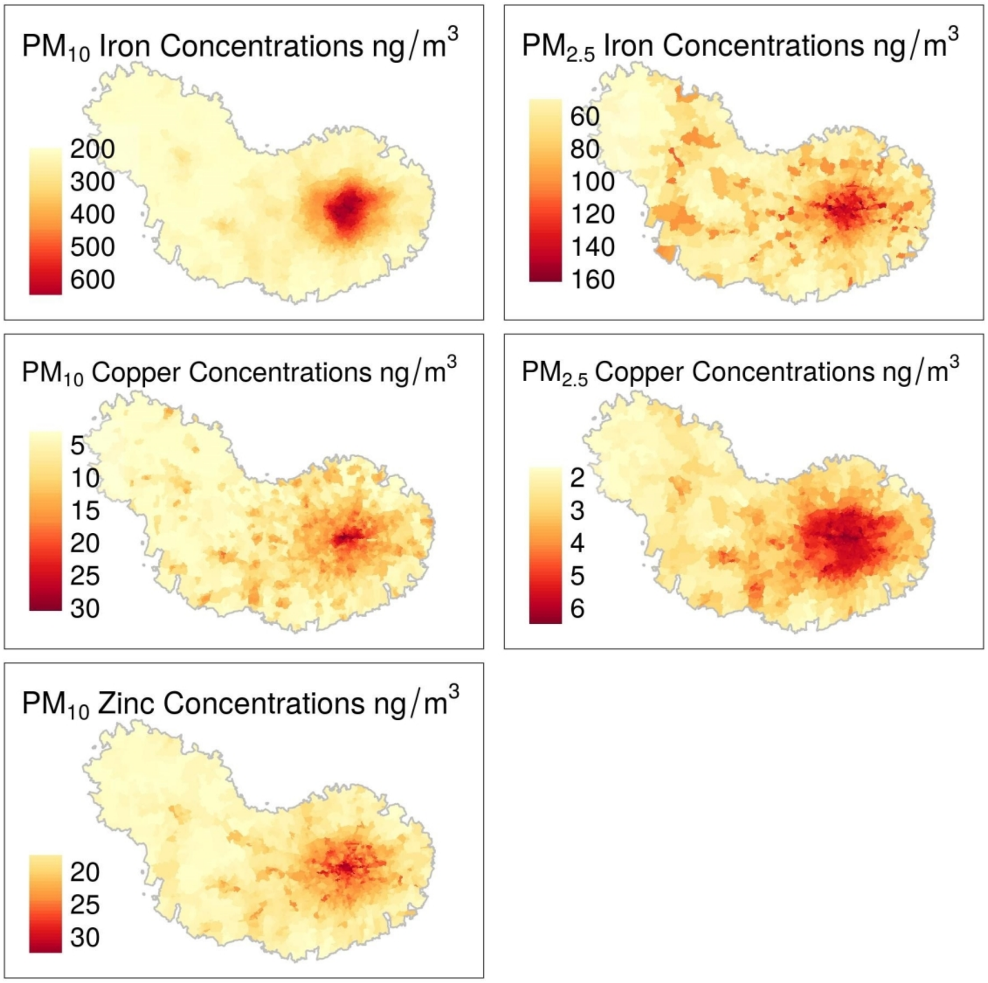


Maps of the metal exposures population weighted by ward. Contains National Statistics and Ordnance Survey data © Crown copyright and database right 2013.

155x153mm (300 x 300 DPI)

Supplementary material to:

**Associations between metal constituents of ambient particulate matter and mortality in England; an ecological study**

**Table S1:** Poisson regression confounder effects from the two models (i) using metals from $PM_{10}$ and (ii) metals from $PM_{2.5}$ for all the health outcomes. Mean, lower and upper bound of the 95% credible interval of the inter-decile relative risk (RR).

| Outcomes | Model | Confounders | RR | CI 95% |
|---|---|---|---|---|
| Cardiovascular mortality | All Metals in $PM_{10}$ VIF=9.14 | IMD | 1.098 | (1.02,1.182) |
| | | % Asian | 0.982 | (0.921,1.046) |
| | | % White | 0.817 | (0.729,0.915) |
| | | Tobacco expenditure | 1.197 | (1.133,1.265) |
| | All Metals in $PM_{2.5}$ VIF=9.04 | IMD | 1.095 | (1.02,1.177) |
| | | % Asian | 0.987 | (0.926,1.052) |
| | | % White | 0.824 | (0.737,0.922) |
| | | Tobacco expenditure | 1.192 | (1.135,1.253) |
| Respiratory mortality | All Metals in $PM_{10}$ VIF=8.93 | IMD | 1.188 | (1.073,1.315) |
| | | % Asian | 0.887 | (0.813,0.967) |
| | | % White | 0.822 | (0.704,0.959) |
| | | Tobacco expenditure | 1.301 | (1.206,1.403) |
| | All Metals in $PM_{2.5}$ VIF=8.81 | IMD | 1.183 | (1.07,1.306) |
| | | % Asian | 0.892 | (0.817,0.973) |
| | | % White | 0.846 | (0.725,0.986) |
| | | Tobacco expenditure | 1.301 | (1.214,1.393) |
| Lung cancer incidence | All Metals in $PM_{10}$ VIF =8.60 | IMD | 1.390 | (1.261,1.532) |
| | | % Asian | 0.851 | (0.790,0.916) |
| | | % White | 0.932 | (0.818,1.062) |
| | | Tobacco expenditure | 1.472 | (1.366,1.586) |
| | All Metals in $PM_{2.5}$ VIF=7.72 | IMD | 1.404 | (1.276,1.544) |
| | | % Asian | 0.846 | (0.786,0.910) |
| | | % White | 0.955 | (0.839,1.086) |
| | | Tobacco expenditure | 1.468 | (1.373,1.569) |

For peer review only

**Table S2:** Pearson Correlation between the adjusted annual mean concentrations of PM-metals and the adjusted annual mean PM concentrations ($PM_{2.5}$ and $PM_{10}$).

| | | $PM_{2.5}$ CU | $PM_{2.5}$ FE | $PM_{10}$ CU | $PM_{10}$ FE | $PM_{10}$ ZN | $PM_{2.5}$ | $PM_{10}$ |
|---|---|---|---|---|---|---|---|---|
| $PM_{2.5}$ | Correlation | .862** | .899** | .896** | .895** | .731** | 1 | .925** |
| | p-value | <0.001 | <0.001 | <0.001 | <0.001 | <0.001 | | <0.001 |
| $PM_{10}$ | Correlation | .825** | .877** | .866** | .889** | .747** | .925** | 1 |
| | p-value | <0.001 | <0.001 | <0.001 | <0.001 | <0.001 | <0.001 | |

For peer review only

**Figure S1.** A graphical presentation of the confounding and causal mechanism, linking exposure and adverse health outcomes.

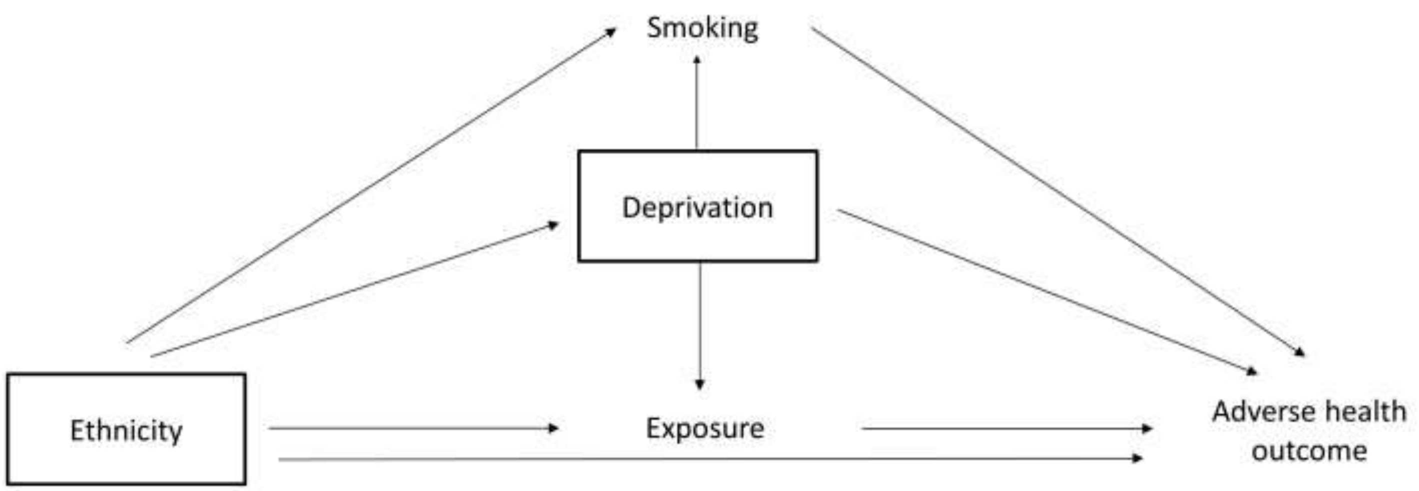


only

**Figure S2.** Maps of the confounders in quintiles: proportion of Asian people, proportion of white people, modified index of multiple deprivations, and tobacco spends. Contains National Statistics and Ordnance Survey data © Crown copyright and database right 2013.

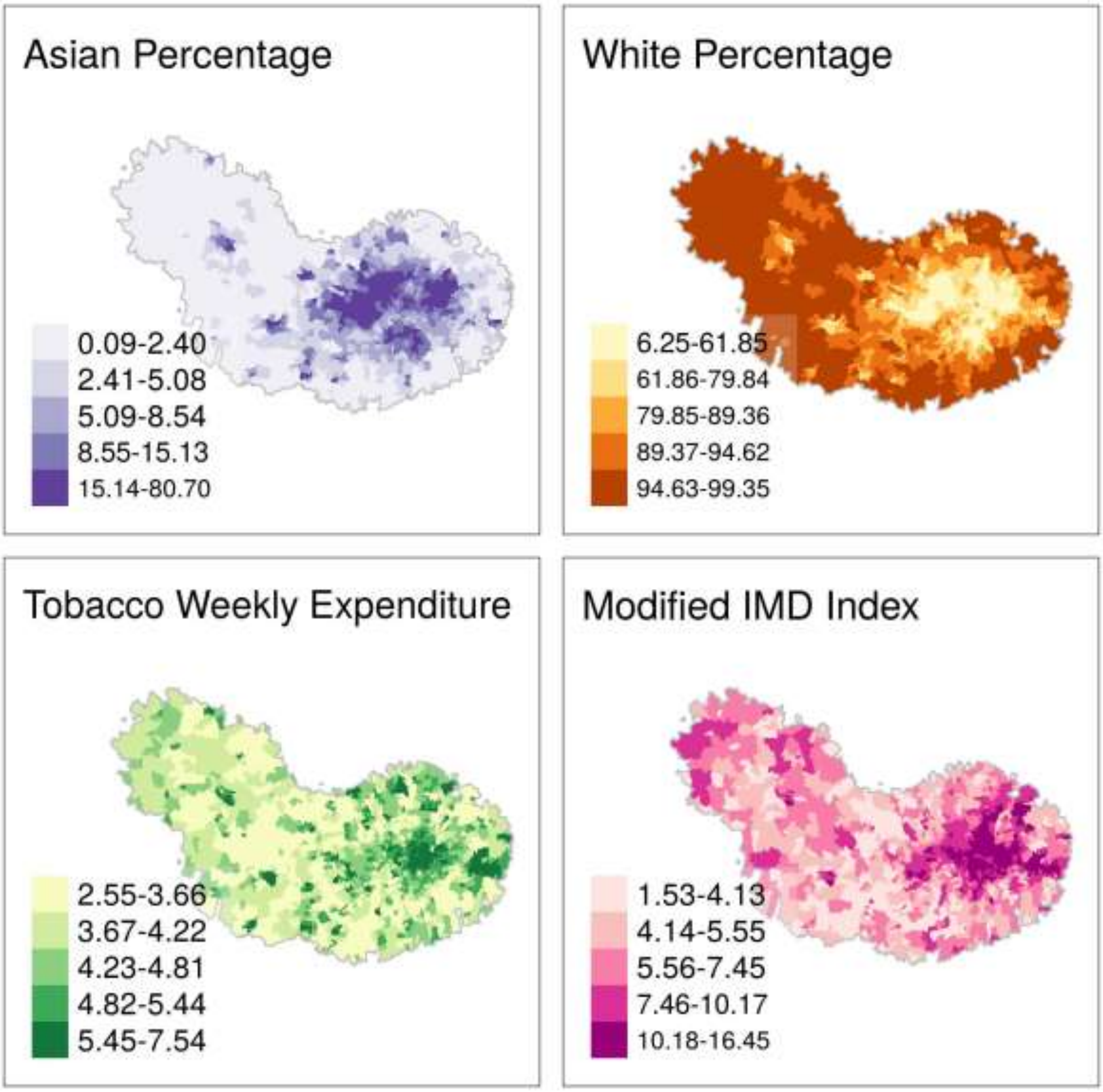

**STROBE 2007 (v4) Statement—Checklist of items that should be included in reports of *cross-sectional studies***

| Section/Topic | Item # | Recommendation | Reported on page # |
|---|---|---|---|
| **Title and abstract** | 1 | (*a*) Indicate the study's design with a commonly used term in the title or the abstract | 1 |
| | | (*b*) Provide in the abstract an informative and balanced summary of what was done and what was found | 3 |
| **Introduction** | | | |
| Background/rationale | 2 | Explain the scientific background and rationale for the investigation being reported | 5 |
| Objectives | 3 | State specific objectives, including any prespecified hypotheses | 5 |
| **Methods** | | | |
| Study design | 4 | Present key elements of study design early in the paper | 5 |
| Setting | 5 | Describe the setting, locations, and relevant dates, including periods of recruitment, exposure, follow-up, and data collection | 5-6 |
| Participants | 6 | (*a*) Give the eligibility criteria, and the sources and methods of selection of participants | 5-6, Figure 1 |
| Variables | 7 | Clearly define all outcomes, exposures, predictors, potential confounders, and effect modifiers. Give diagnostic criteria, if applicable | 5-6, Figure 2 and supplementary Figure S1 |
| Data sources/ measurement | 8* | For each variable of interest, give sources of data and details of methods of assessment (measurement). Describe comparability of assessment methods if there is more than one group | 5-6 |

For peer review only

| | | | |
|---|---|---|---|
| Bias | 9 | Describe any efforts to address potential sources of bias | 5, 7 |
| Study size | 10 | Explain how the study size was arrived at | Figure 1 |
| Quantitative variables | 11 | Explain how quantitative variables were handled in the analyses. If applicable, describe which groupings were chosen and why | 5-6 |
| Statistical methods | 12 | (*a*) Describe all statistical methods, including those used to control for confounding | 6 |
| | | (*b*) Describe any methods used to examine subgroups and interactions | |
| | | (*c*) Explain how missing data were addressed | No missing data! |
| | | (*d*) If applicable, describe analytical methods taking account of sampling strategy | NA – used all deaths, all population in area |
| | | (*e*) Describe any sensitivity analyses | None conducted |
| **Results** | | | |
| Participants | 13* | (a) Report numbers of individuals at each stage of study—eg numbers potentially eligible, examined for eligibility, confirmed eligible, included in the study, completing follow-up, and analysed | Table 1 and table 2 |
| | | (b) Give reasons for non-participation at each stage | NA |
| | | (c) Consider use of a flow diagram | NA |
| Descriptive data | 14* | (a) Give characteristics of study participants (eg demographic, clinical, social) and information on exposures and potential confounders | Supplementary Table S1 and Figure S1, 6 |
| | | (b) Indicate number of participants with missing data for each variable of interest | NA |
| Outcome data | 15* | Report numbers of outcome events or summary measures | 6 |

For peer review only

| | | | |
|---|---|---|---|
| Main results | 16 | (*a*) Give unadjusted estimates and, if applicable, confounder-adjusted estimates and their precision (eg, 95% confidence interval). Make clear which confounders were adjusted for and why they were included | 9-10, Table 1 and 3, Supplementary table S1 |
| | | (*b*) Report category boundaries when continuous variables were categorized | NA (no categorization) |
| | | (*c*) If relevant, consider translating estimates of relative risk into absolute risk for a meaningful time period | NA |
| Other analyses | 17 | Report other analyses done—eg analyses of subgroups and interactions, and sensitivity analyses | NA |
| **Discussion** | | | |
| Key results | 18 | Summarise key results with reference to study objectives | 7 |
| Limitations | 19 | Discuss limitations of the study, taking into account sources of potential bias or imprecision. Discuss both direction and magnitude of any potential bias | 8 |
| Interpretation | 20 | Give a cautious overall interpretation of results considering objectives, limitations, multiplicity of analyses, results from similar studies, and other relevant evidence | 8 |
| Generalisability | 21 | Discuss the generalisability (external validity) of the study results | 8 |
| **Other information** | | | |
| Funding | 22 | Give the source of funding and the role of the funders for the present study and, if applicable, for the original study on which the present article is based | 2 |

*Give information separately for cases and controls in case-control studies and, if applicable, for exposed and unexposed groups in cohort and cross-sectional studies.

For peer review only

**Note:** An Explanation and Elaboration article discusses each checklist item and gives methodological background and published examples of transparent reporting. The STROBE checklist is best used in conjunction with this article (freely available on the Web sites of PLoS Medicine at http://www.plosmedicine.org/, Annals of Internal Medicine at http://www.annals.org/, and Epidemiology at http://www.epidem.com/). Information on the STROBE Initiative is available at www.strobe-statement.org.

For peer review only

# Associations between metal constituents of ambient particulate matter and mortality in England; an ecological study

| | |
|---|---|
| Journal: | *BMJ Open* |
| Manuscript ID | bmjopen-2019-030140.R2 |
| Article Type: | Original research |
| Date Submitted by the Author: | 18-Sep-2019 |
| Complete List of Authors: | Lavigne, Aurore; Université de Lille 3 UFR MIME, UFR MIME, Domaine universitaire du Pont de Bois<br>Freni Sterrantino , Anna ; Imperial College London, Epidemiology and Biostatistics<br>Liverani, Silvia; Queen Mary University of London, School of Mathematical Sciences<br>Blangiardo, Marta; Imperial College London, MRC-PHE Centre for Environment and Health, Department of Epidemiology and Biostatistics, School of Public Health<br>de Hoogh, Kees; Swiss Tropical and Public Health Institute; University of Basel<br>Molitor, John ; Oregon State University CAPS, School of Biological and Population Health Sciences, College of Public Health and Human Sciences<br>Hansell, Anna ; University of Leicester, |
| <b>Primary Subject Heading</b>: | Epidemiology |
| Secondary Subject Heading: | Health policy |
| Keywords: | Particulate matter elements, multipollutant effect, correlation, Metals, Environmental Epidemiology, EPIDEMIOLOGY |
| | |

**Research Article**

# Associations between metal constituents of ambient particulate matter and mortality in England; an ecological study

Aurore Lavigne*, Université Lille 3, UFR MIME, Domaine Universitaire du Pont de Bois, Villeneuve d'ascq Cedex, France

Anna Freni-Sterrantino*, Small Area Health Statistics Unit, Imperial College London, United Kingdom

Silvia Liverani, School of Mathematical Science, Queen Mary University of London, United Kingdom

Marta Blangiardo, Department of Epidemiology and Biostatistics, Imperial College London, United Kingdom

Kees de Hoogh, Swiss Tropical and Public Health Institute, Basel, Switzerland and University of Basel, Basel, Switzerland

John Molitor, School of Biological and Population Health Sciences, College of Public Health and Human Sciences, Oregon State University, USA

Anna L. Hansell, Centre for Environmental Health and Sustainability, George Davies Centre, Dept of Health Sciences, University of Leicester, United Kingdom

Small Area Health Statistics Unit, Imperial College London, United Kingdom
**Corresponding author: Anna L. Hansell,**

Centre for Environmental Health and Sustainability, George Davies Centre, Dept of Health Sciences, University of Leicester, United Kingdom 15 Lancaster Rd, Leicester LE1 7HA
Email: ah618@leicester.ac.uk
* joint first authors

The authors report no conflict of interest

**Availability of computing code and data:** The mortality cancer and population data used in this article were supplied by the Office for National Statistics (ONS), derived from the national mortality, cancer and birth registrations and the Census. SAHSU does not have permission to supply data to third parties, but the health and population data can be obtained from ONS on application. Air pollution estimates by ward for 2008-11 for the study area and code used can be obtained on request from the authors.
No identifiable information will be shared with any other organization. The scripts used can be provided by request from the authors.

**Acknowledgements**

We would like to acknowledge and thank Prof. John Gulliver, Dr Gary Fuller, Dr David Morley and Prof. Nicky Best for their useful comments.
CACI tobacco expenditure data is © Copyright 1996-2014 CACI Limited.

**Ethics and Information Governance statement**



For peer review only - http://bmjopen.bmj.com/site/about/guidelines.xhtml

SAHSU holds approvals from the National Research Ethics Service - reference 12/LO/0566 and 12/LO/0567 - and from the Health Research Authority Confidentially Advisory Group (HRA-CAG) for Section 251 support (HRA - 14/CAG/1039) for use of the health data used in this research.

The research was funded/part funded by the National Institute for Health Research Health Protection Research Unit (NIHR HPRU) in Health Impact of Environmental Hazards at King's College London in partnership with Public Health England (PHE) and Imperial College London. The views expressed are those of the author(s) and not necessarily those of the NHS, the NIHR, the Department of Health & Social Care or Public Health England".

**Funding**

The research project was funded through Medical Research Council (grant G09018401) and the Small Area Health Statistics Unit. The work of the UK Small Area Health Statistics Unit is funded by Public Health England as part of the MRC-PHE Centre for Environment and Health, funded also by the UK Medical Research Council.
The air pollution exposure assessments used in the research leading to these results was funded by the European Community's Seventh Framework Program (FP7/2007-2011) projects ESCAPE (grant agreement 211250) and TRANSPHORM (ENV.2009.1.2.2.1).

**Author's Contribution**

AL and AFS drafted the paper and ran the statistical analyses. KH provided exposure data. SL, JM and MB advised on the statistical methods. AH designed he study. All the authors provided intellectual input, interpreted the results, and helped to revise the manuscript. All authors approved the final version of the manuscript and agreed to be accountable for all the aspects of the work in ensuring that questions related to the accuracy or integrity of any part of the work are appropriately investigated and resolved. AH is the guarantor of this paper.

**Word Count: 2198**

**Figures: 2**

**Tables: 3**

For peer review only



For peer review only - http://bmjopen.bmj.com/site/about/guidelines.xhtml

## Abstract

**Objectives** To investigate long-term associations between metal components of particulate matter and mortality and lung cancer incidence

**Design** Small area (ecological) study

**Setting** Population living in all wards (~9000 individuals per ward) in the London and Oxford area of England, comprising 13.6 million individuals

**Exposure and Outcome measures** We used land use regression (LUR) models originally used in the Transport related Air Pollution and Health impacts – Integrated Methodologies for Assessing Particulate Matter (TRANSPHORM) study to estimate exposure to copper, iron and zinc in ambient air particulate matter. We examined associations of metal exposure with Office for National Statistics mortality data from cardiovascular (CVD) and respiratory causes and with lung cancer incidence in 2008-11.

**Results** There were 108,478 CVD deaths, 48,483 respiratory deaths and 24,849 incident cases of lung cancer in the study period and area. Using Poisson regression models adjusted for area-level deprivation, tobacco sales and ethnicity, we found associations between cardiovascular mortality and $PM_{2.5}$ copper with interdecile range (IDR-2.6-5.7 ng/m$^3$) and IDR Relative risk (RR) 1.005 (95%CI 1.001, 1.009) and between respiratory mortality and $PM_{10}$ zinc (IDR 1135-153 ng/m$^3$) and IDR RR 1.136 (95%CI 1.010, 1.277). We did not find relevant associations for lung cancer incidence. Metal elements were highly correlated.

**Conclusion** Our analysis showed small but not fully consistent adverse associations between mortality and particulate metal exposures likely derived from non-tailpipe road traffic emissions (brake and tyre-wear), which have previously been associated with increases in inflammatory markers in the blood.


**Keywords:** Particulate matter elements, multipollutant effect, correlation, Metals, Environmental Epidemiology



For peer review only - http://bmjopen.bmj.com/site/about/guidelines.xhtml

**Strengths and limitations of this study**

- One of the largest studies to explore exposure to metal components of ambient air in relation to mortality and lung cancer incidence, with 13.6 million population
- A large number of cases: 108,478 CVD deaths, 48,483 respiratory deaths and 24,849 incident cases of lung cancer in the study period and area, providing good statistical power to examine small excess risks
- Established exposure models, developed and evaluated with measurements from a standardised monitoring campaign
- An ecological study using registry data, without access to individual-level confounders other than age and sex
- Metals were very highly correlated so multi-pollutant models could not be used

For peer review only

## Introduction

Chronic exposure to toxic substances in particulate matter (PM) with aerodynamic diameter less than 10µm ($PM_{10}$)[1-3] and 2.5µm ($PM_{2.5}$)[4] is associated with increased mortality levels from cardiovascular disease[1 5]. Some studies also show links between this long term exposure to traffic-related air pollution and lung cancer or respiratory mortality[6]. It has been suggested that metal components of particulate matter may in part be responsible for toxic effects of air pollution on the cardiovascular and respiratory system[7].

In the Transport related Air Pollution and Health impacts – Integrated Methodologies for Assessing Particulate Matter (TRANSPHORM) study, copper zinc and iron content of particulate matter ($PM_{10}$ and $PM_{2.5}$) were found to be associated -positively and significantly- with increases in inflammatory markers in the blood[8], which might be expected to be associated with increased risks of cardiovascular and other diseases. However, a separate TRANSPHORM study[9] analysis of 19 cohorts with 9,545 CVD deaths, did not find any statistically significant associations with metal (or other) particulate components ($PM_{10}$ or $PM_{2.5}$). Here we use the same datasets to examine associations with mortality using a much larger dataset than TRANSPHORM study[9], to estimate particulate metal exposures for a population of 13.6 million living in and near London, England, with 108,478 CVD deaths and additionally 48,483 respiratory deaths and 24,849 incident cases of lung cancer.

## Methods

Our study region covered a 10,782 km$^2$ area around London and Oxford (Figure 1) in 1533 wards, an English Census area classification (primary unit of the English electoral geography) with a mean surface area ~7.0 km$^2$ and average 8,892 inhabitants per ward, in our study period.

### Exposure data

In the region of London and Oxford particulate matter was monitored during the years 2010-2011 as part of the European Study of Cohorts and Air Pollution Effects (ESCAPE) project[10 11]. Filters measuring $PM_{10}$ and $PM_{2.5}$ from the ESCAPE project were analyzed for elemental composition and de Hoogh et al. (2013)[12] developed land use regression (LUR) models for a number of the elemental components including metals as part of the TRANSPHORM project. These models were used to predict $PM_{10}$ and $PM_{2.5}$ elemental composition for our study population for 2010-2011. In brief, twenty sites were monitored for three 2-week periods[10] and $PM_{2.5}$ and $PM_{10}$ were separately collected using Harvard impactors. Their elemental composition was analyzed using energy dispersive X ray fluorescence. The association of PM elemental components with land use covariates relative to traffic, population, industry, or nature was evaluated with LUR models. Then, local estimates at the postcode level were predicted and aggregated at the Super Output Area (SOA) level, with a population-weighted mean, for all SOAs in the study. Exposure was assigned for each case or incidence at post-code level.
In the analyses, we used copper (Cu), iron (Fe) and zinc (Zn) in the $PM_{10}$ fraction and copper and iron in the $PM_{2.5}$ fraction, all linked to non-tailpipe emissions[12]. LUR models for this selection of elements showed a good leave-one-out validation, explaining more than 77% ($R^2$) of the observed variability.

### Confounder data – deprivation, ethnicity and smoking data

To adjust for possible confounders in this study, we included area-level ethnicity from Census 2011 and accounted for percent of White and Asian people per ward as covariates in the models. We also used the 2007 Index of Multiple Deprivation (IMD) as a relative measure of area-level deprivation (publicly available from the Department for Communities and Local Government data.gov.uk). This combines seven domains; 'income', 'employment', 'education', 'barriers to housing and services', 'crime', 'health' and 'living envi-



For peer review only - http://bmjopen.bmj.com/site/about/guidelines.xhtml

ronment'. The latter is divided into two subdomains: 'indoor' measuring the quality of housing and 'outdoor' linked to air quality and road traffic accidents [13]. We excluded from the study the 'health' and 'outdoor living environment' domains[14], since we examined associations between health outcomes and air pollution measures. The remaining domains were linearly combined to generate a 'modified IMD' relative score used in the analysis. High values of the modified IMD indicate higher deprivation. As a proxy for smoking, we used ward level tobacco expenditure (pounds/week/inhabitant) data obtained from CACI (CACI tobacco expenditure data is © Copyright 1996-2014 CACI Limited).

**Health data**

Mortality counts for cardiovascular (CDC10 I00-I99) and respiratory (CDC10 J00-J99) disease and lung cancer incidence counts (C33 and C34 ICD10 codes) were extracted for 2008-2011 from Office National Statistics data held by the Small Area Health Statistics Unit (SAHSU), which provide 100% coverage of deaths. The counts were then adjusted by sex and 5-year age band.

**Patient and Public Involvement.**

Patients were not involved in the development of the research question or the design and conducting of the study.

**Statistical analysis**

The effect of PM exposure to copper, iron and zinc on health outcomes were analyzed with Poisson regression (a generalized linear model) of count data at small area (ward) level, implemented in a Bayesian framework with spatial residuals, see supplementary figure 1 for a graphical representation of the possible causal mechanism.

Let $Y_i$ denote the number of cases recorded in the spatial unit $i$ and $E_i$ the expected count taking into account the age and sex structure of the population at risk (internal standardization). Then, using Poisson regression, $Y_i$ is assumed to follow a Poisson distribution with mean equal to $E_i RR_i$ such that

$$\log(RR_i) = \mu + \sum_{j=1}^{p_1} \alpha_j Confound_{ij} + \beta PM_{ik} + U_i.$$

Here, $\mu$ is the model intercept, $Confound_{ij}$ denotes the value of the confounder $j\,(1, \ldots, p_1)$ for area $i(1, \ldots, n)$, similarly $PM_{ik}$ stands for the PM $k(1, \ldots, p_2)$exposures , $U_i$ is a spatial random effect, modelled with an intrinsic conditional autoregressive model [15], accounting for the spatial dependence of residuals. The coefficients $\alpha_j$ and $\beta$ indicate the linear effect of the confounders and PM-metals on the log relative risk.
For each health outcome, the analysis was performed separately for elemental constituents of $PM_{10}$ and $PM_{2.5}$. A second model was fitted, for each PM metal constituents and as measure of multicollinearity the and variation inflation factor (VIF) is provided.
Both models are inferred using the Bayesian approach in R-package INLA[16]. We used the non-informative priors proposed as default in R-INLA and standardized confounders.

Regression parameters are expressed per Interdecile range (IDR) relative risk, i.e. the increase of the relative risk when the level of covariates increases from the 10$^{th}$ to 90$^{th}$ centile; the posterior mean and 95% credible bounds are given.



For peer review only - http://bmjopen.bmj.com/site/about/guidelines.xhtml

## Results

There were 108,478 cardiovascular and 48,483 respiratory deaths and 24,849 incident lung cancer cases in the study area for 2008-11 (Table 1 and Table S1). We have reported summary descriptive statistics for Standard Mortality/Incidence Rates (SMR/SIRs), metal constituents of PM and confounders, stratifying the wards between the 10th percentile of exposure 90th percentile of PM2.5 Copper. SMRs/SIRs, metal constituents of PM, area-level deprivation, non-white ethnicity and tobacco sales (smoking proxy) were all higher in wards in the $90^{th}$ vs. $10^{th}$ percentile $PM_{2.5}$ copper.
Maps of the spatial distribution of the covariates and elemental concentrations show that highest values were in Greater London Area, with iron and zinc also high in wards with motorways (Figure 2). The percentage population ethnicity for wards had a median of 77% white and 9% Asian ethnicity (predominantly of South Asian origin). Most of the areas with low percentage of White population was concentrated in Greater London, which also had higher percentage of Asian (supplementary material figure S2).

For peer review only

**Table 1**: Descriptive statistics of health outcomes, modelled particulate metal concentrations, deprivation score, and ethnicity covariates for the 1533 wards in the study area in 2008-11, all stratified by $PM_{2.5}$ Copper >10$^{th}$, 10$^{th}$-90$^{th}$ and >90$^{th}$ quantile.

| | | **Cu[1] $PM_{2.5}$** | | | | | | | | | | | |
|---|---|---|---|---|---|---|---|---|---|---|---|---|---|
| | | **10th centile (n=153)** | | | | **10th-90th centile (n=1225)** | | | | **90th centile (n=154)** | | | |
| | | **10th centile** | **mean** | **median** | **90th Centile** | **10th centile** | **mean** | **median** | **90th Centile** | **10th centile** | **mean** | **median** | **90th Centile** |
| **Health outcomes** | | Standard Mortality/Incidence Ratio (ratio across whole study area =1.00) | | | | | | | | | | | |
| Cardiovascular mortality | | 0.57 | 0.83 | 0.86 | 1.13 | 0.72 | 0.99 | 1.01 | 1.33 | 0.63 | 0.96 | 1.00 | 1.35 |
| Respiratory mortality | | 0.46 | 0.81 | 0.81 | 1.20 | 0.61 | 0.98 | 1.02 | 1.46 | 0.50 | 0.94 | 0.94 | 1.36 |
| Lung cancer incidence | | 0.40 | 0.81 | 0.86 | 1.33 | 0.53 | 0.95 | 0.99 | 1.53 | 0.64 | 1.14 | 1.16 | 1.73 |
| **Modelled metal concentrations using LUR** | | Metals in ng/m$^3$ | | | | | | | | | | | |
| | **LOOCV* $R^2$ (for LUR**)** | | | | | | | | | | | | |
| Cu $PM_{10}$ | 0.95 | 3.06 | 4.17 | 4.98 | 8.41 | 6.17 | 11.48 | 11.47 | 16.46 | 14.56 | 20.44 | 20.39 | 25.89 |
| Fe $PM_{10}$ | 0.77 | 200.96 | 206.47 | 208.16 | 218.68 | 222.50 | 267.62 | 318.16 | 490.62 | 474.78 | 606.63 | 586.77 | 646.40 |
| Zn $PM_{10}$ | 0.95 | 15.30 | 15.73 | 15.81 | 16.38 | 16.65 | 19.27 | 19.90 | 24.01 | 24.20 | 26.28 | 26.79 | 30.52 |
| Fe $PM_{2.5}$ | 0.92 | 43.23 | 48.14 | 49.85 | 57.21 | 51.60 | 70.80 | 75.87 | 107.83 | 112.35 | 130.96 | 130.22 | 144.84 |
| **Area-level confounders** | | | | | | | | | | | | | |



For peer review only

For peer review only - http://bmjopen.bmj.com/site/about/guidelines.xhtml

| | | | | | | | | | | | | |
|---|---|---|---|---|---|---|---|---|---|---|---|---|
| IMD (modified Index of multiple deprivation) | 3.26 | 4.85 | 5.15 | 7.41 | 3.35 | 6.29 | 6.83 | 11.30 | 7.91 | 11.22 | 10.99 | 13.24 |
| % of Asian | 0.01 | 0.01 | 0.01 | 0.02 | 0.02 | 0.07 | 0.12 | 0.26 | 0.07 | 0.11 | 0.15 | 0.31 |
| % of White | 0.95 | 0.97 | 0.97 | 0.99 | 0.46 | 0.86 | 0.78 | 0.95 | 0.43 | 0.62 | 0.60 | 0.77 |
| Tobacco expenditure (pounds/ week/inhabitant) | 3.19 | 3.88 | 3.96 | 4.86 | 3.41 | 4.46 | 4.57 | 5.96 | 4.45 | 5.65 | 5.53 | 6.55 |

*Leave one out cross-validation (LOOCV)
**Land Use Regression (LUR)
[1] Cu $PM_{10}$ Metals in $ng/m^3$ LOOCV $R^2$ =0.79

For peer review only

The individual linear effect of each elemental constitute of particulate matter evaluated with the Poisson regression adjusted for confounders is displayed in Table 2 and Table S2 in Supplementary Material. Statistically significant associations with PM metal concentrations were identified for cardiovascular and respiratory mortality but not lung cancer incidence. For cardiovascular mortality, copper in the $PM_{2.5}$ fraction was associated with a small increased risk RR 1.005 (95%CI 1.001, 1.009) per interdecile range (IDR) but iron had an apparent protective association (RR 0.042 95%CI 0.002, 0.995) albeit with extremely high uncertainty. For respiratory mortality, the copper in the $PM_{10}$ fraction had a very small protective association (RR 0.988 95%CI 0.978, 0.998), but $PM_{10}$ zinc was associated with an increased mortality risk (RR 1.136 95%CI 1.010, 1.277).

**Table 2**: Individual effects of metals, estimated with Poisson regression, on cardiovascular mortality, respiratory mortality and lung cancer incidence adjusted for tobacco weekly expenditure, IMD (index of multiple deprivation) and percentage of Asian and White population. Mean and lower and upper bounds of the credible intervals of the inter-decile relative risk (RR).

| | Metal | RR | 95% credible intervals |
|---|---|---|---|
| **Cardiovascular mortality** | Cu $PM_{10}$ | 0.994 | (0.987,1.001) |
| | Fe $PM_{10}$ | 0.319 | (0.037,2.779) |
| | Zn $PM_{10}$ | 1.073 | (0.985,1.169) |
| | Cu $PM_{2.5}$ | 1.005 | (1.001,1.009) |
| | Fe $PM_{2.5}$ | 0.042 | (0.002,0.995) |
| **Respiratory mortality** | Cu $PM_{10}$ | 0.988 | (0.978,0.998) |
| | Fe $PM_{10}$ | 0.649 | (0.033,12.767) |
| | Zn $PM_{10}$ | 1.136 | (1.010,1.277) |
| | Cu $PM_{2.5}$ | 1.003 | (0.998,1.009) |
| | Fe $PM_{2.5}$ | 0.980 | (0.013,72.673) |
| **Lung cancer incidence** | Cu $PM_{10}$ | 0.998 | (0.912,1.091) |
| | Fe $PM_{10}$ | 0.973 | (0.830,1.142) |
| | Zn $PM_{10}$ | 0.995 | (0.910,1.089) |
| | Cu $PM_{2.5}$ | 1.092 | (0.943,1.225) |
| | Fe $PM_{2.5}$ | 0.969 | (0.889,1.057) |



For peer review only - http://bmjopen.bmj.com/site/about/guidelines.xhtml

The elements were highly correlated: 0.88 for $PM_{2.5}$ elements and 0.82-0.92 for $PM_{10}$ elements (Table 3). For $PM_{10}$ the Pearson correlation between copper and zinc was 0.85, and for $PM_{2.5}$ the correlation between copper and iron was 0.88. The metal constituents showed high correlation with $PM_{2.5}$ and $PM_{10}$ mass concentrations for $PM_{2.5}$ and metals in $PM_{2.5}$ was 0.86-0.89 and 0.73-0.89 for $PM_{10}$ metals; for $PM_{10}$ and $PM_{10}$ metals 0.74-0.88 and 0.86-0.89 for metals in $PM_{2.5}$ (see supplementary table S3).
Thus, it is not possible to definitively attribute an association with one metal element given the inter-dependence.

**Table 3**: Pearson inter-correlation(r) between the particle metals (PM) metals (n=1533).

| | **$PM_{10}$ Copper** | **$PM_{10}$ Iron** | **$PM_{10}$ Zinc** | **$PM_{2.5}$ Iron** | **$PM_{2.5}$ Copper** |
|---|---|---|---|---|---|
| **$PM_{10}$ Copper** | 1 | | | | |
| **$PM_{10}$ Iron** | 0.85 | 1 | | | |
| **$PM_{10}$ Zinc** | 0.85 | 0.92 | 1 | | |
| **$PM_{2.5}$ Iron** | 0.82 | 0.91 | 0.93 | 1 | |
| **$PM_{2.5}$ Copper** | 0.75 | 0.89 | 0.90 | 0.88 | 1 |

In the model fit, for each group of metals by PM, we have found that area-level deprivation (IMD) and weekly tobacco spend had a clear adverse association with cardiovascular mortality, respiratory mortality and lung cancer incidence (supplementary material Table S3), with moderate high value of VIF. On the contrary, the proportions of White and Asian people in wards was associated with lower risks for the three diseases, suggesting a weak influence of the ethnic composition of the population on mortality/incidence rate.

**Discussion**

This ecological study at small area level examined associations between modelled particulate metal (copper, iron and zinc) concentrations in relation to cardiovascular and respiratory mortality and lung cancer incidence in and around Greater London covering 13.6 million population with approximately 110,000 cardiorespiratory deaths and 25,000 new lung cancer cases. While the results did not find evidence of positive association between ambient particulate metal concentrations and lung cancer incidence, Poisson regression suggested copper in the $PM_{2.5}$ fraction was statistically significant associated with increased cardiovascular mortality risk and $PM_{10}$ zinc with respiratory mortality risk. Results for metal constituents were not fully consistent within our study for the same element in $PM_{2.5}$ and $PM_{10}$ size fractions. Metal exposures were highly correlated so it is difficult to definitively attribute an association with one metal element.

Advantages of our study include the use of extremely large datasets with population coverage giving good statistical power to detect even very small associations. Another advantage was the use of standardized exposure models developed from standardized monitoring campaigns to estimate spatial variability in long-term exposures. While exposure data were derived from LUR models that showed good predictability, they may still misclassify true exposure as (i) prediction is good but not perfect (ii) using a model of exposure at



For peer review only - http://bmjopen.bmj.com/site/about/guidelines.xhtml

residence as a proxy for personal exposure. A limitation in our exposure assessment is the limited number of monitoring sites, 20, which potentially can lead to overfitting of the developed LUR models[17]. Providing that densities of measurement sites and estimation sites (wards) are similar, Szpiro and Paciorek (2013)[18] show that in case of over-smoothing of the exposure, the association between outcomes and exposure may be underestimated. In our case, over-smoothing likely occurs and this issue may partially explain our difficulty to show evidence of adverse associations between health outcomes and exposures to particulate elements. As most other ambient air pollution studies, we use outdoor concentration of pollutants at residence, without taking into account indoor levels, travel exposure or places of work. The correlation between indoor and outdoor concentration is high for fine particulate ($PM_{2.5}$)[19], suggesting that ignoring the indoor concentration is a small issue. However, in the London region, the difference of exposure at home and workplace may be different, since a part of the population living in suburban areas work in the city center, where exposures are higher. Another limitation is that we used LUR models predicting particulate metals in 2010-2011 to look at associations with mortality during 2008-11. Our exposure estimates should also be representative of the preceding two years and should capture deaths related to short- and intermediate-long term influences. However, we used an ecological study design with limited ability to control for confounders at the individual level.

There are a limited number of other health studies looking at copper, zinc and iron metal components of particulates. Three studies looking at long-term effects using similarly derived estimates from the TRANSPHORM project as used here but much smaller numbers of health events than this study, found significant associations with inflammatory markers in blood but not health events. Hampel et al. [8] found positive statistically significant associations between $PM_{2.5}$ copper and $PM_{10}$ iron with high-sensitivity C-reactive protein and $PM_{2.5}$ zinc with fibrinogen in five European cohorts with available biomarkers (>17,000 measurements). Wolf et al [20] found elevated but non-significant positive associations with copper, zinc and iron constituents of particulates ($PM_{10}$ or $PM_{2.5}$) with incident coronary events in 11 cohorts (5,157 events), while Wang et al. [9] did not find long-term positive associations with cardiovascular mortality (9545 deaths) in 19 European cohorts where exposure results from a single year were applied over 2-20 years follow-up, in some cases retrospectively. A further study, the California teachers study [21] found positive and significant associations between $PM_{2.5}$ copper estimated in 2001-7 and contemporaneous ischemic heart disease deaths (1085 events) and elevated but non-significant associations with $PM_{2.5}$ iron and other metals.
We did not find associations with lung cancer incidence. While toxicological studies suggest that metals in airborne particulates are genotoxic[22], the reason we did not find an association even in our large sample size may be because our exposure measures relate to a similar time frame as the health outcome. Studies finding associations of particulates with lung cancer have typically considered 10 or more years follow-up[23].

Short-term associations of metal components of particulates with mortality were examined in a systematic review of time series studies of fine-particle components and health published up to 2013 [24]. Zinc, indicative of road dust and possibly a result of tyre wear, was associated with daily mortality in eight of eleven studies included in the review. The subsequently published MED-PARTICLES time-series analysis in five European cities Basagaña et al. [17] found positive significant short-term associations with $PM_{10}$ copper iron and zinc and $PM_{2.5}$ iron with cardiovascular hospitalizations and $PM_{10}$ and $PM_{2.5}$ zinc for respiratory disease hospitalizations, but no significant associations were seen for mortality.



For peer review only - http://bmjopen.bmj.com/site/about/guidelines.xhtml

The reason that results for metal constituents of particulates are not completely consistent across studies, may be that metal concentrations serve as a proxy for oxidative potential[25]. Within the study area and in the analysis, the TRANSPHORM metal particulate measurements used to derive the land use regression models were highly correlated with oxidative potential of the particulates as measured using ascorbate (Pearson r = 0.93 for copper, 0.95 for iron, 0.67 for zinc)[25]. The high correlations between metal constituents of particulates raise the possibility that observed associations for one metal actually relate to another element that was better estimated. The high correlations also preclude conducting multi-pollutant analyses using Poisson regression.

## Conclusion

We found positive and significant associations suggestive of small increased risk of cardiovascular and respiratory mortality but not lung cancer incidence in Greater London and surroundings in relation to metal concentrations of ambient particulate matter, which are likely derived from non-tailpipe road traffic emissions (brake and tyre-wear). Findings are consistent with a previous study finding associations of particulate metals with inflammatory markers, but further work is needed to better define exposures to airborne metal elements and non-tailpipe emissions.

## References

1. Pope CA, Burnett RT, Thurston GD, et al. Cardiovascular Mortality and Long-Term Exposure to Particulate Air Pollution. *Epidemiological Evidence of General Pathophysiological Pathways of Disease* 2004;109(1):71-77. doi: 10.1161/01.Cir.0000108927.80044.7f
2. Puett RC, Hart JE, Suh H, et al. Particulate Matter Exposures, Mortality, and Cardiovascular Disease in the Health Professionals Follow-up Study. *Environmental Health Perspectives* 2011;119(8):1130-35. doi: 10.1289/ehp.1002921
3. Zhang LW, Chen X, Xue XD, et al. Long-term exposure to high particulate matter pollution and cardiovascular mortality: a 12-year cohort study in four cities in northern China. *Environ Int* 2014;62:41-7. doi: 10.1016/j.envint.2013.09.012 [published Online First: 2013/10/29]
4. Brook RD, Rajagopalan S, Pope CA, et al. Particulate Matter Air Pollution and Cardiovascular Disease. *An Update to the Scientific Statement From the American Heart Association* 2010;121(21):2331-78. doi: 10.1161/CIR.0b013e3181dbece1
5. Crouse DL, Peters PA, van Donkelaar A, et al. Risk of nonaccidental and cardiovascular mortality in relation to long-term exposure to low concentrations of fine particulate matter: a Canadian national-level cohort study. *Environ Health Perspect* 2012;120(5):708-14. doi: 10.1289/ehp.1104049 [published Online First: 2012/02/09]
6. Beelen R, Hoek G, van den Brandt PA, et al. Long-Term Effects of Traffic-Related Air Pollution on Mortality in a Dutch Cohort (NLCS-AIR Study). *Environmental Health Perspectives* 2008;116(2):196-202. doi: 10.1289/ehp.10767
7. Li H, Qian X, Wang Qg. Heavy Metals in Atmospheric Particulate Matter: A Comprehensive Understanding Is Needed for Monitoring and Risk Mitigation. *Environmental Science & Technology* 2013;47(23):13210-11. doi: 10.1021/es404751a



For peer review only - http://bmjopen.bmj.com/site/about/guidelines.xhtml

8. Hampel R, Peters A, Beelen R, et al. Long-term effects of elemental composition of particulate matter on inflammatory blood markers in European cohorts. *Environment International* 2015;82:76-84. doi: https://doi.org/10.1016/j.envint.2015.05.008
9. Wang M, Beelen R, Stafoggia M, et al. Long-term exposure to elemental constituents of particulate matter and cardiovascular mortality in 19 European cohorts: Results from the ESCAPE and TRANSPHORM projects. *Environment International* 2014;66:97-106. doi: https://doi.org/10.1016/j.envint.2014.01.026
10. Eeftens M, Tsai M-Y, Ampe C, et al. Spatial variation of PM2.5, PM10, PM2.5 absorbance and PMcoarse concentrations between and within 20 European study areas and the relationship with NO2 - Results of the ESCAPE project. *Atmospheric Environment* 2012;62:303-17. doi: 10.1016/j.atmosenv.2012.08.038
11. Tsai M-Y, Hoek G, Eeftens M, et al. Spatial variation of PM elemental composition between and within 20 European study areas — Results of the ESCAPE project. *Environment International* 2015;84:181-92. doi: https://doi.org/10.1016/j.envint.2015.04.015
12. de Hoogh K, Wang M, Adam M, et al. Development of Land Use Regression Models for Particle Composition in Twenty Study Areas in Europe. *Environmental Science & Technology* 2013;47(11):5778-86. doi: 10.1021/es400156t
13. deprivation Eio. https://www.gov.uk/government/statistics/english-indices-of-deprivation-2010, 2010.
14. Adams J, White M. Removing the health domain from the Index of Multiple Deprivation 2004—effect on measured inequalities in census measure of health. *Journal of Public Health* 2006;28(4):379-83. doi: 10.1093/pubmed/fdl061
15. Besag J, York J, Mollié A. Bayesian image restoration, with two applications in spatial statistics. *Annals of the Institute of Statistical Mathematics* 1991;43(1):1-20. doi: 10.1007/bf00116466
16. Martins TG, Simpson D, Lindgren F, et al. Bayesian computing with INLA:New features. *Computational Statistics & Data Analysis* 2013;67:68-83. doi: 10.1016/j.csda.2013.04.014
17. Basagaña X, Rivera M, Aguilera I, et al. Effect of the number of measurement sites on land use regression models in estimating local air pollution. *Atmospheric Environment* 2012;54:634-42. doi: https://doi.org/10.1016/j.atmosenv.2012.01.064
18. Szpiro AA, Paciorek CJ. Measurement error in two-stage analyses, with application to air pollution epidemiology. *Environmetrics* 2013;24(8):501-17. doi: 10.1002/env.2233
19. Brunekreef B, Holgate ST. Air pollution and health. *The Lancet* 2002;360(9341):1233-42. doi: https://doi.org/10.1016/S0140-6736(02)11274-8
20. Wolf K, Stafoggia M, Cesaroni G, et al. Long-term Exposure to Particulate Matter Constituents and the Incidence of Coronary Events in 11 European Cohorts. *Epidemiology* 2015;26(4):565-74. doi: 10.1097/ede.0000000000000300
21. Ostro B, Hu J, Goldberg D, et al. Associations of Mortality with Long-Term Exposures to Fine and Ultrafine Particles, Species and Sources: Results from the California Teachers Study Cohort. *Environmental Health Perspectives* 2015;123(6):549-56. doi: 10.1289/ehp.1408565
22. Bocchi C, Bazzini C, Fontana F, et al. Characterization of urban aerosol: Seasonal variation of genotoxicity of the water-soluble portion of PM2.5 and PM1. *Mutation Research/Genetic Toxicology and Environmental Mutagenesis* 2019;841:23-30. doi: https://doi.org/10.1016/j.mrgentox.2019.04.005
23. Raaschou-Nielsen O, Andersen ZJ, Beelen R, et al. Air pollution and lung cancer incidence in 17 European cohorts: prospective analyses from the European Study of Cohorts for Air Pollution Effects (ESCAPE). *The Lancet Oncology* 2013;14(9):813-22. doi: https://doi.org/10.1016/S1470-2045(13)70279-1



For peer review only - http://bmjopen.bmj.com/site/about/guidelines.xhtml

24. Atkinson RW, Analitis A, Samoli E, et al. Short-term exposure to traffic-related air pollution and daily mortality in London, UK. *Journal Of Exposure Science And Environmental Epidemiology* 2015;26:125. doi: 10.1038/jes.2015.65

https://www.nature.com/articles/jes201565#supplementary-information

25. Gulliver J, Morley D, Dunster C, et al. Land use regression models for the oxidative potential of fine particles (PM2.5) in five European areas. *Environmental Research* 2018;160:247-55. doi: https://doi.org/10.1016/j.envres.2017.10.002

For peer review only



For peer review only - http://bmjopen.bmj.com/site/about/guidelines.xhtml

## Figures

**Figure 1.** Study Area compromising London and Oxford areas, with major roads and motorways. In the inset the area localization with regard to England map. Contains National Statistics and Ordnance Survey data © Crown copyright and database right 2013.

**Figure 2.** Maps of the metal exposures population weighted by ward. Contains National Statistics and Ordnance Survey data © Crown copyright and database right 2013.

For peer review only



For peer review only - http://bmjopen.bmj.com/site/about/guidelines.xhtml

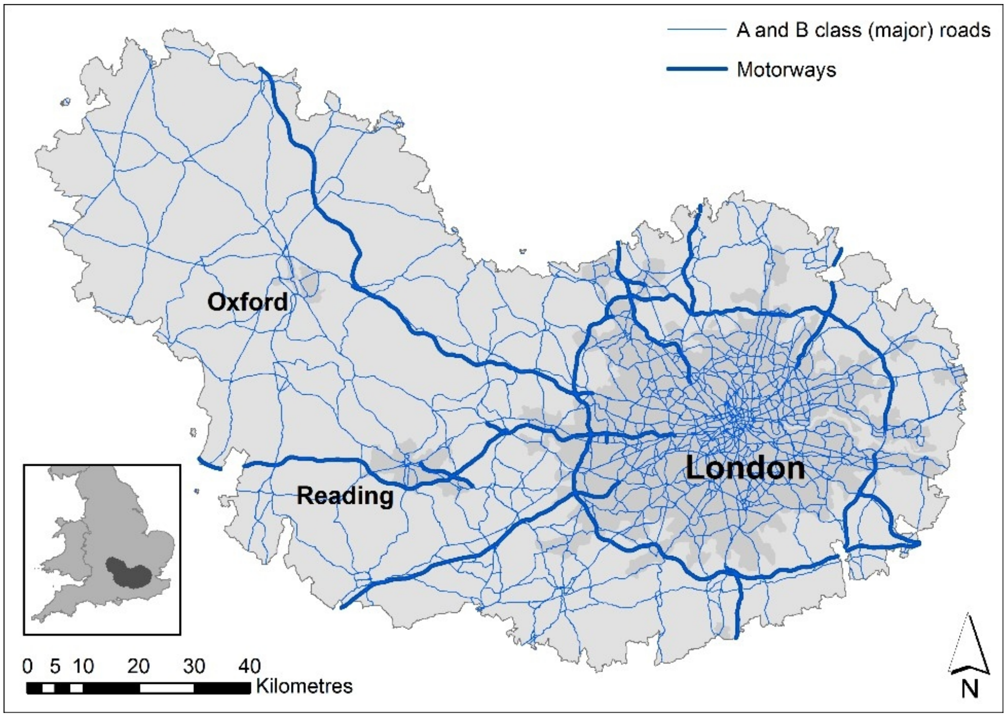


Study Area compromising London and Oxford areas, with major roads and motorways. In the inset the area localization with regard to England map. Contains National Statistics and Ordnance Survey data © Crown copyright and database right 2013.

112x79mm (220 x 220 DPI)

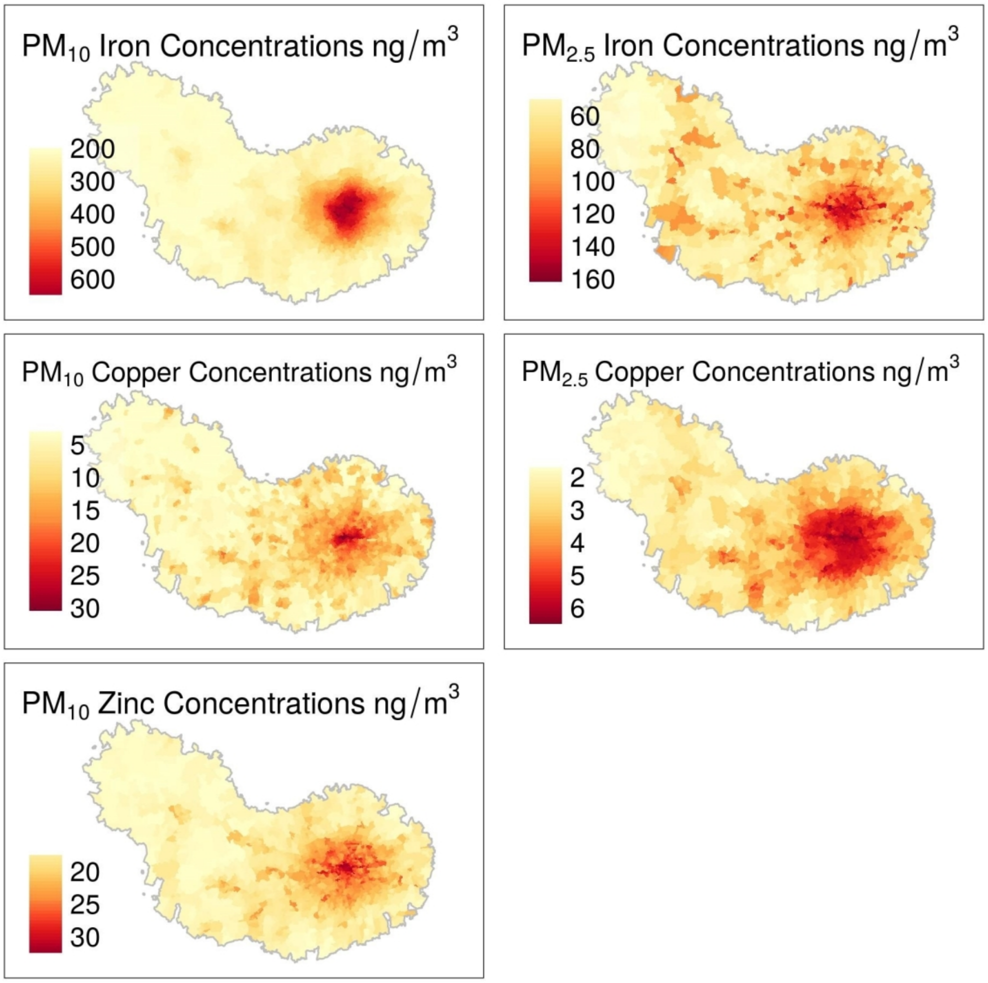


Maps of the metal exposures population weighted by ward. Contains National Statistics and Ordnance Survey data © Crown copyright and database right 2013.

155x153mm (300 x 300 DPI)

Supplementary material to:

**Associations between metal constituents of ambient particulate matter and mortality in England; an ecological study**

**Table S1**: Descriptive statistics of health outcomes, modelled particulate metal concentrations, deprivation score, and ethnicity covariates for the 1533 wards in the study area in 2008-11.

| | 10th centile | mean | median | 90th centile | LOOCV $R^2$ (for LUR) |
|---|---|---|---|---|---|
| **Health outcomes** | Rates of health outcome (number of cases per hundred thousand people) | | | | |
| Cardiovascular mortality | 117.50 | 215.97 | 203.20 | 327.87 | |
| Respiratory mortality | 42.85 | 96.34 | 87.85 | 160.41 | |
| Lung cancer incidence | 25.06 | 48.44 | 45.75 | 75.86 | |
| **Modelled metal concentrations using LUR** | Metals in $ng/m^3$ | | | | |
| Cu $PM_{10}$ | 7.0 | 13.3 | 13.1 | 19.8 | 0.95 |
| Fe $PM_{10}$ | 223.2 | 378.9 | 357.0 | 596.7 | 0.95 |
| Zn $PM_{10}$ | 113.5 | 135.2 | 139.5 | 153.0 | 0.77 |
| Cu PM2.5 | 2.6 | 4.3 | 4.6 | 5.7 | 0.79 |
| Fe PM2.5 | 51.6 | 86.8 | 82.8 | 129.0 | 0.92 |
| **Area-level confounders** | | | | | |
| Deprivation (modified IMD) | 3.45 | 7.08 | 6.47 | 11.78 | |
| % of Asian | 2 | 13 | 9 | 33 | |
| % of White | 38 | 72 | 77 | 95 | |
| Tobacco expenditure (pounds/week/inhabitant) | 3.40 | 4.61 | 4.48 | 6.03 | |

**Table S2:** Poisson regression confounder effects from the two models (i) using metals from $PM_{10}$ and (ii) metals from $PM_{2.5}$ for all the health outcomes. Mean, lower and upper bound of the 95% credible interval (CI) of the inter-decile relative risk (RR).

| Outcomes | Model | Confounders | RR | CI 95% |
|---|---|---|---|---|
| Cardiovascular mortality | All Metals in $PM_{10}$ $VIF^2$=9.14 | IMD[1] | 1.098 | (1.02,1.182) |
| | | % Asian | 0.982 | (0.921,1.046) |
| | | % White | 0.817 | (0.729,0.915) |
| | | Tobacco expenditure | 1.197 | (1.133,1.265) |
| | All Metals in $PM_{2.5}$ $VIF^2$=9.04 | IMD[1] | 1.095 | (1.02,1.177) |
| | | % Asian | 0.987 | (0.926,1.052) |
| | | % White | 0.824 | (0.737,0.922) |
| | | Tobacco expenditure | 1.192 | (1.135,1.253) |
| Respiratory mortality | All Metals in $PM_{10}$ $VIF^2$=8.93 | IMD[1] | 1.188 | (1.073,1.315) |
| | | % Asian | 0.887 | (0.813,0.967) |
| | | % White | 0.822 | (0.704,0.959) |

For peer review only

| | | | | |
|---|---|---|---|---|
| | | Tobacco expenditure | 1.301 | (1.206,1.403) |
| | All Metals in $PM_{2.5}$ VIF[2]=8.81 | IMD[1] | 1.183 | (1.07,1.306) |
| | | % Asian | 0.892 | (0.817,0.973) |
| | | % White | 0.846 | (0.725,0.986) |
| | | Tobacco expenditure | 1.301 | (1.214,1.393) |
| Lung cancer incidence | All Metals in $PM_{10}$ VIF =8.60 | IMD[1] | 1.390 | (1.261,1.532) |
| | | % Asian | 0.851 | (0.790,0.916) |
| | | % White | 0.932 | (0.818,1.062) |
| | | Tobacco expenditure | 1.472 | (1.366,1.586) |
| | All Metals in $PM_{2.5}$ VIF[2]=7.72 | IMD[1] | 1.404 | (1.276,1.544) |
| | | % Asian | 0.846 | (0.786,0.910) |
| | | % White | 0.955 | (0.839,1.086) |
| | | Tobacco expenditure | 1.468 | (1.373,1.569) |

[1]IMD Indices of multiple deprivation
[2]VIF Variance Inflation Factor

**Table S3:** Pearson Correlation(r) between the adjusted annual mean concentrations of PM-metals and the adjusted annual mean PM concentrations ($PM_{2.5}$ and $PM_{10}$).

| N=1533 | $PM_{2.5}$ CU | $PM_{2.5}$ FE | $PM_{10}$ CU | $PM_{10}$ FE | $PM_{10}$ ZN | $PM_{2.5}$ | $PM_{10}$ |
|---|---|---|---|---|---|---|---|
| $PM_{2.5}$ Correlation | .862** | .899** | .896** | .895** | .731** | 1 | .925** |
| $PM_{10}$ Correlation | .825** | .877** | .866** | .889** | .747** | .925** | 1 |

** All the correlation signifcant at p< 0.001

For peer review only

**Figure S1.** A graphical presentation of the confounding and causal mechanism, linking exposure and adverse health outcomes.

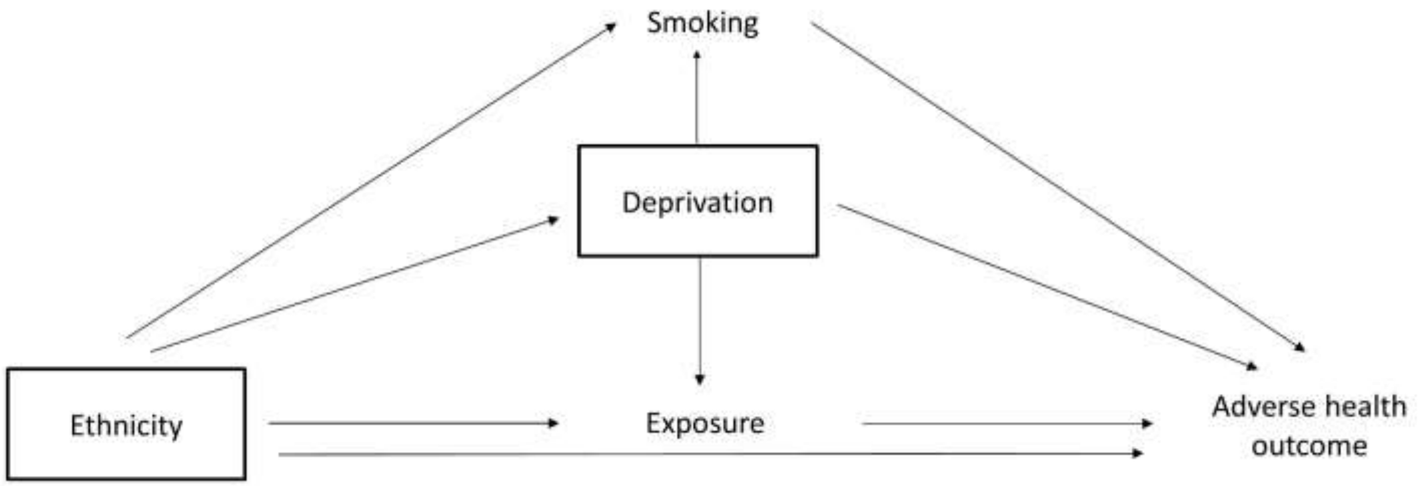


only

**Figure S2.** Maps of the confounders in quintiles: proportion of Asian people, proportion of white people, modified index of multiple deprivations, and tobacco spends. Contains National Statistics and Ordnance Survey data © Crown copyright and database right 2013.

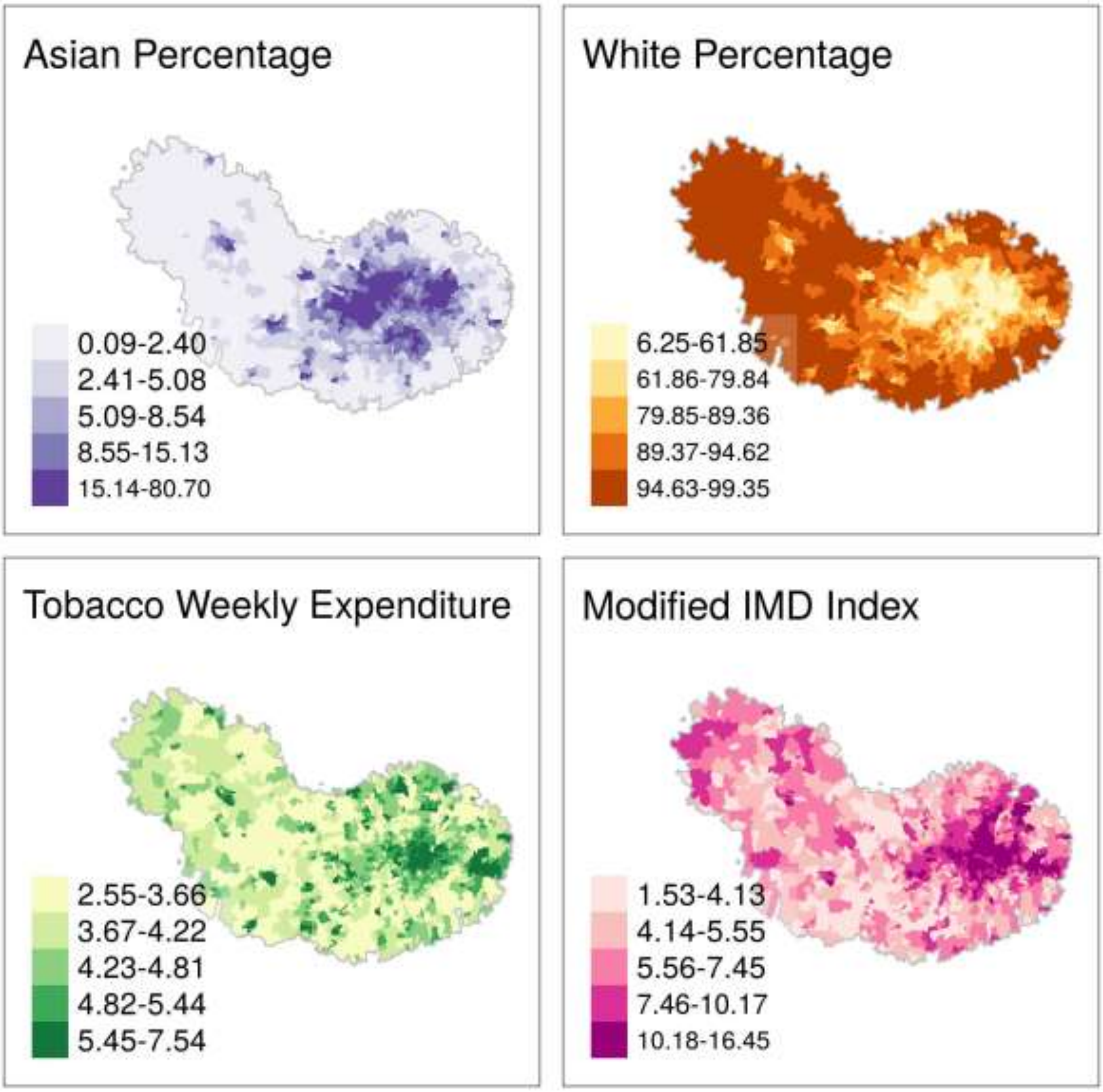

**STROBE 2007 (v4) Statement—Checklist of items that should be included in reports of *cross-sectional studies***

| Section/Topic | Item # | Recommendation | Reported on page # |
|---|---|---|---|
| **Title and abstract** | 1 | (*a*) Indicate the study's design with a commonly used term in the title or the abstract | 1 |
| | | (*b*) Provide in the abstract an informative and balanced summary of what was done and what was found | 3 |
| **Introduction** | | | |
| Background/rationale | 2 | Explain the scientific background and rationale for the investigation being reported | 5 |
| Objectives | 3 | State specific objectives, including any prespecified hypotheses | 5 |
| **Methods** | | | |
| Study design | 4 | Present key elements of study design early in the paper | 5 |
| Setting | 5 | Describe the setting, locations, and relevant dates, including periods of recruitment, exposure, follow-up, and data collection | 5-6 |
| Participants | 6 | (*a*) Give the eligibility criteria, and the sources and methods of selection of participants | 5-6, Figure 1 |
| Variables | 7 | Clearly define all outcomes, exposures, predictors, potential confounders, and effect modifiers. Give diagnostic criteria, if applicable | 5-6, Figure 2 and supplementary Figure S1 |
| Data sources/ measurement | 8* | For each variable of interest, give sources of data and details of methods of assessment (measurement). Describe comparability of assessment methods if there is more than one group | 5-6 |

For peer review only

| | | | |
|---|---|---|---|
| Bias | 9 | Describe any efforts to address potential sources of bias | 5, 7 |
| Study size | 10 | Explain how the study size was arrived at | Figure 1 |
| Quantitative variables | 11 | Explain how quantitative variables were handled in the analyses. If applicable, describe which groupings were chosen and why | 5-6 |
| Statistical methods | 12 | (*a*) Describe all statistical methods, including those used to control for confounding | 6 |
| | | (*b*) Describe any methods used to examine subgroups and interactions | |
| | | (*c*) Explain how missing data were addressed | No missing data! |
| | | (*d*) If applicable, describe analytical methods taking account of sampling strategy | NA – used all deaths, all population in area |
| | | (*e*) Describe any sensitivity analyses | None conducted |
| **Results** | | | |
| Participants | 13* | (a) Report numbers of individuals at each stage of study—eg numbers potentially eligible, examined for eligibility, confirmed eligible, included in the study, completing follow-up, and analysed | Table 1 and table 2 |
| | | (b) Give reasons for non-participation at each stage | NA |
| | | (c) Consider use of a flow diagram | NA |
| Descriptive data | 14* | (a) Give characteristics of study participants (eg demographic, clinical, social) and information on exposures and potential confounders | Supplementary Table S1 and Figure S1, 6 |
| | | (b) Indicate number of participants with missing data for each variable of interest | NA |
| Outcome data | 15* | Report numbers of outcome events or summary measures | 6 |

For peer review only

| | | | |
|---|---|---|---|
| Main results | 16 | (*a*) Give unadjusted estimates and, if applicable, confounder-adjusted estimates and their precision (eg, 95% confidence interval). Make clear which confounders were adjusted for and why they were included | 9-10, Table 1 and 3, Supplementary table S1 |
| | | (*b*) Report category boundaries when continuous variables were categorized | NA (no categorization) |
| | | (*c*) If relevant, consider translating estimates of relative risk into absolute risk for a meaningful time period | NA |
| Other analyses | 17 | Report other analyses done—eg analyses of subgroups and interactions, and sensitivity analyses | NA |
| **Discussion** | | | |
| Key results | 18 | Summarise key results with reference to study objectives | 7 |
| Limitations | 19 | Discuss limitations of the study, taking into account sources of potential bias or imprecision. Discuss both direction and magnitude of any potential bias | 8 |
| Interpretation | 20 | Give a cautious overall interpretation of results considering objectives, limitations, multiplicity of analyses, results from similar studies, and other relevant evidence | 8 |
| Generalisability | 21 | Discuss the generalisability (external validity) of the study results | 8 |
| **Other information** | | | |
| Funding | 22 | Give the source of funding and the role of the funders for the present study and, if applicable, for the original study on which the present article is based | 2 |

*Give information separately for cases and controls in case-control studies and, if applicable, for exposed and unexposed groups in cohort and cross-sectional studies.

For peer review only

**Note:** An Explanation and Elaboration article discusses each checklist item and gives methodological background and published examples of transparent reporting. The STROBE checklist is best used in conjunction with this article (freely available on the Web sites of PLoS Medicine at http://www.plosmedicine.org/, Annals of Internal Medicine at http://www.annals.org/, and Epidemiology at http://www.epidem.com/). Information on the STROBE Initiative is available at www.strobe-statement.org.

For peer review only

BMJ Open

# Associations between metal constituents of ambient particulate matter and mortality in England; an ecological study

| | |
|---|---|
| Journal: | *BMJ Open* |
| Manuscript ID | bmjopen-2019-030140.R3 |
| Article Type: | Original research |
| Date Submitted by the Author: | 22-Oct-2019 |
| Complete List of Authors: | Lavigne, Aurore; Université de Lille 3 UFR MIME, UFR MIME, Domaine universitaire du Pont de Bois<br>Freni Sterrantino , Anna ; Imperial College London, Epidemiology and Biostatistics<br>Liverani, Silvia; Queen Mary University of London, School of Mathematical Sciences<br>Blangiardo, Marta; Imperial College London, MRC-PHE Centre for Environment and Health, Department of Epidemiology and Biostatistics, School of Public Health<br>de Hoogh, Kees; Swiss Tropical and Public Health Institute; University of Basel<br>Molitor, John ; Oregon State University CAPS, School of Biological and Population Health Sciences, College of Public Health and Human Sciences<br>Hansell, Anna ; University of Leicester, |
| <b>Primary Subject Heading</b>: | Epidemiology |
| Secondary Subject Heading: | Health policy, Epidemiology |
| Keywords: | Particulate matter elements, multipollutant effect, correlation, Metals, Environmental Epidemiology, EPIDEMIOLOGY |
| | |

SCHOLARONE™
Manuscripts

Research Article

# Associations between metal constituents of ambient particulate matter and mortality in England; an ecological study

Aurore Lavigne*, Université Lille 3, UFR MIME, Domaine Universitaire du Pont de Bois, Villeneuve d'ascq Cedex, France

Anna Freni-Sterrantino*, Small Area Health Statistics Unit, Imperial College London, United Kingdom

Silvia Liverani, School of Mathematical Science, Queen Mary University of London, United Kingdom

Marta Blangiardo, Department of Epidemiology and Biostatistics, Imperial College London, United Kingdom

Kees de Hoogh, Swiss Tropical and Public Health Institute, Basel, Switzerland and University of Basel, Basel, Switzerland

John Molitor, School of Biological and Population Health Sciences, College of Public Health and Human Sciences, Oregon State University, USA

Anna L. Hansell, Centre for Environmental Health and Sustainability, George Davies Centre, Dept of Health Sciences, University of Leicester, United Kingdom

Small Area Health Statistics Unit, Imperial College London, United Kingdom

**Corresponding author: Anna L. Hansell,**

Centre for Environmental Health and Sustainability, George Davies Centre, Dept of Health Sciences,
University of Leicester, United Kingdom 15 Lancaster Rd, Leicester LE1 7HA
Email: ah618@leicester.ac.uk
* joint first authors

The authors report no conflict of interest



For peer review only - http://bmjopen.bmj.com/site/about/guidelines.xhtml

**Availability of computing code and data:** The mortality cancer and population data used in this article were supplied by the Office for National Statistics (ONS), derived from the national mortality, cancer and birth registrations and the Census. SAHSU does not have permission to supply data to third parties, but the health and population data can be obtained from ONS on application. Air pollution estimates by ward for 2008-11 for the study area and code used can be obtained on request from the authors.

No identifiable information will be shared with any other organization. The scripts used can be provided by request from the authors.

**Acknowledgements**

We would like to acknowledge and thank Prof. John Gulliver, Dr Gary Fuller, Dr David Morley and Prof. Nicky Best for their useful comments.

CACI tobacco expenditure data is © Copyright 1996-2014 CACI Limited.

**Ethics and Information Governance statement**

SAHSU holds approvals from the National Research Ethics Service - reference 12/LO/0566 and 12/LO/0567 - and from the Health Research Authority Confidentially Advisory Group (HRA-CAG) for Section 251 support (HRA - 14/CAG/1039) for use of the health data used in this research.

The research was funded/part funded by the National Institute for Health Research Health Protection Research Unit (NIHR HPRU) in Health Impact of Environmental Hazards at King's College London in partnership with Public Health England (PHE) and Imperial College London. The views expressed are those of the author(s) and not necessarily those of the NHS, the NIHR, the Department of Health & Social Care or Public Health England".

**Funding**

The research project was funded through Medical Research Council (grant G09018401) and the Small Area Health Statistics Unit. The work of the UK Small Area Health Statistics Unit is funded by Public Health England as part of the MRC-PHE Centre for Environment and Health, funded also by the UK Medical Research Council.

The air pollution exposure assessments used in the research leading to these results was funded by the European Community's Seventh Framework Program (FP7/2007-2011) projects ESCAPE (grant agreement 211250) and TRANSPHORM (ENV.2009.1.2.2.1).



For peer review only - http://bmjopen.bmj.com/site/about/guidelines.xhtml

## Author's Contribution

AL and AFS drafted the paper and ran the statistical analyses. KH provided exposure data. SL, JM and MB advised on the statistical methods. AH designed the study. All the authors provided intellectual input, interpreted the results, and helped to revise the manuscript. All authors approved the final version of the manuscript and agreed to be accountable for all the aspects of the work in ensuring that questions related to the accuracy or integrity of any part of the work are appropriately investigated and resolved. AH is the guarantor of this paper.

**Word Count: 2198**

**Figures: 2**

**Tables: 3**

For peer review only

## Abstract



For peer review only - http://bmjopen.bmj.com/site/about/guidelines.xhtml

**Objectives** To investigate long-term associations between metal components of particulate matter and mortality and lung cancer incidence

**Design** Small area (ecological) study

**Setting** Population living in all wards (~9000 individuals per ward) in the London and Oxford area of England, comprising 13.6 million individuals

**Exposure and Outcome measures** We used land use regression (LUR) models originally used in the Transport related Air Pollution and Health impacts – Integrated Methodologies for Assessing Particulate Matter (TRANSPHORM) study to estimate exposure to copper, iron and zinc in ambient air particulate matter. We examined associations of metal exposure with Office for National Statistics mortality data from cardiovascular (CVD) and respiratory causes and with lung cancer incidence in 2008-11.

**Results** There were 108,478 CVD deaths, 48,483 respiratory deaths and 24,849 incident cases of lung cancer in the study period and area. Using Poisson regression models adjusted for area-level deprivation, tobacco sales and ethnicity, we found associations between cardiovascular mortality and $PM_{2.5}$ copper with interdecile range (IDR-2.6-5.7 ng/$m^3$) and IDR Relative risk (RR) 1.005 (95%CI 1.001, 1.009) and between respiratory mortality and $PM_{10}$ zinc (IDR 1135-153 ng/$m^3$) and IDR RR 1.136 (95%CI 1.010, 1.277). We did not find relevant associations for lung cancer incidence. Metal elements were highly correlated.

**Conclusion** Our analysis showed small but not fully consistent adverse associations between mortality and particulate metal exposures likely derived from non-tailpipe road traffic emissions (brake and tyre-wear), which have previously been associated with increases in inflammatory markers in the blood.

**Keywords:** Particulate matter elements, multipollutant effect, correlation, Metals, Environmental Epidemiology



For peer review only - http://bmjopen.bmj.com/site/about/guidelines.xhtml

## Strengths and limitations of this study

- One of the largest studies to explore exposure to metal components of ambient air in relation to mortality and lung cancer incidence, with 13.6 million population
- A large number of cases: 108,478 CVD deaths, 48,483 respiratory deaths and 24,849 incident cases of lung cancer in the study period and area, providing good statistical power to examine small excess risks
- Established exposure models, developed and evaluated with measurements from a standardised monitoring campaign
- An ecological study using registry data, without access to individual-level confounders other than age and sex
- Metals were very highly correlated so multi-pollutant models could not be used

For peer review only

## Introduction

Chronic exposure to toxic substances in particulate matter (PM) with aerodynamic diameter less than 10μm ($PM_{10}$)[1-3] and 2.5μm ($PM_{2.5}$)[4] is associated with increased mortality levels from cardiovascular disease[1 5]. Some studies also show links between this long term exposure to traffic-related air pollution and lung cancer or respiratory mortality[6]. It has been suggested that metal components of particulate matter may in part be responsible for toxic effects of air pollution on the cardiovascular and respiratory system[7].

In the Transport related Air Pollution and Health impacts – Integrated Methodologies for Assessing Particulate Matter (TRANSPHORM) study, copper zinc and iron content of particulate matter ($PM_{10}$ and $PM_{2.5}$) were found to be associated -positively and significantly- with increases in inflammatory markers in the blood[8], which might be expected to be associated with increased risks of cardiovascular and other diseases. However, a separate TRANSPHORM study[9] analysis of 19 cohorts with 9,545 CVD deaths, did not find any statistically significant associations with metal (or other) particulate components ($PM_{10}$ or $PM_{2.5}$). Here we use the same datasets to examine associations with mortality using a much larger dataset than TRANSPHORM study[9], to estimate particulate metal exposures for a population of 13.6 million living in and near London, England, with 108,478 CVD deaths and additionally 48,483 respiratory deaths and 24,849 incident cases of lung cancer.

## Methods

Our study region covered a 10,782 km$^2$ area around London and Oxford (Figure 1) in 1533 wards, an English Census area classification (primary unit of the English electoral geography) with a mean surface area ~7.0 km$^2$ and average 8,892 inhabitants per ward, in our study period.

## Exposure data

In the region of London and Oxford particulate matter was monitored during the years 2010-2011 as part of the European Study of Cohorts and Air Pollution Effects (ESCAPE) project[10 11]. Filters measuring $PM_{10}$ and $PM_{2.5}$ from the ESCAPE project were analyzed for elemental composition and de Hoogh et al. (2013)[12] developed land use regression (LUR) models for a number of the elemental components including metals as part of the TRANSPHORM project. These models were used to predict $PM_{10}$ and $PM_{2.5}$ elemental composition for our study population for 2010-2011. In



For peer review only - http://bmjopen.bmj.com/site/about/guidelines.xhtml

brief, twenty sites were monitored for three 2-week periods[10] and $PM_{2.5}$ and $PM_{10}$ were separately collected using Harvard impactors. Their elemental composition was analyzed using energy dispersive X ray fluorescence. The association of PM elemental components with land use covariates relative to traffic, population, industry, or nature was evaluated with LUR models. Then, local estimates at the postcode level were predicted and aggregated at the Super Output Area (SOA) level, with a population-weighted mean, for all SOAs in the study. Exposure was assigned for each case or incidence at post-code level.

In the analyses, we used copper (Cu), iron (Fe) and zinc (Zn) in the $PM_{10}$ fraction and copper and iron in the $PM_{2.5}$ fraction, all linked to non-tailpipe emissions[12]. LUR models for this selection of elements showed a good leave-one-out validation, explaining more than 77% ($R^2$) of the observed variability.

For peer review only

**Confounder data – deprivation, ethnicity and smoking data**

To adjust for possible confounders in this study, we included area-level ethnicity from Census 2011 and accounted for percent of White and Asian people per ward as covariates in the models. We also used the 2007 Index of Multiple Deprivation (IMD) as a relative measure of area-level deprivation (publicly available from the Department for Communities and Local Government data.gov.uk). This combines seven domains; 'income', 'employment', 'education', 'barriers to housing and services', 'crime', 'health' and 'living environment'. The latter is divided into two subdomains: 'indoor' measuring the quality of housing and 'outdoor' linked to air quality and road traffic accidents [13]. We excluded from the study the 'health' and 'outdoor living environment' domains[14], since we examined associations between health outcomes and air pollution measures. The remaining domains were linearly combined to generate a 'modified IMD' relative score used in the analysis. High values of the modified IMD indicate higher deprivation. As a proxy for smoking, we used ward level tobacco expenditure (pounds/week/inhabitant) data obtained from CACI (CACI tobacco expenditure data is © Copyright 1996-2014 CACI Limited).

**Health data**

Mortality counts for cardiovascular (CDC10 I00-I99) and respiratory (CDC10 J00-J99) disease and lung cancer incidence counts (C33 and C34 ICD10 codes) were extracted for 2008-2011 from Office National Statistics data held by the Small Area Health Statistics Unit (SAHSU), which provide 100% coverage of deaths. The counts were then adjusted by sex and 5-year age band.

**Patient and Public Involvement.**



For peer review only - http://bmjopen.bmj.com/site/about/guidelines.xhtml

Patients were not involved in the development of the research question or the design and conducting of the study.

## Statistical analysis

The effect of PM exposure to copper, iron and zinc on health outcomes were analyzed with Poisson regression (a generalized linear model) of count data at small area (ward) level, implemented in a Bayesian framework with spatial residuals, see supplementary figure 1 for a graphical representation of the possible causal mechanism.

Let $Y_i$ denote the number of cases recorded in the spatial unit $i$ and $E_i$ the expected count taking into account the age and sex structure of the population at risk (internal standardization). Then, using Poisson regression, $Y_i$ is assumed to follow a Poisson distribution with mean equal to $E_i RR_i$ such that

$$\log(RR_i) = \mu + \sum_{j=1}^{p_1} \alpha_j Confound_{ij} + \beta PM_{ik} + U_i.$$

Here, $\mu$ is the model intercept, $Confound_{ij}$ denotes the value of the confounder $j$ $(1, \ldots, p_1)$ for area $i(1, \ldots, n)$ , similarly $PM_{ik}$ stands for the PM $k(1, \ldots, p_2)$exposures , $U_i$ is a spatial random effect, modelled with an intrinsic conditional autoregressive model [15], accounting for the spatial dependence of residuals. The coefficients $\alpha_j$ and $\beta$ indicate the linear effect of the confounders and PM-metals on the log relative risk.

For each health outcome, the analysis was performed separately for elemental constituents of $PM_{10}$ and $PM_{2.5}$. A second model was fitted, for each PM metal constituents and as measure of multicollinearity the and variation inflation factor (VIF) is provided.

Both models are inferred using the Bayesian approach in R-package INLA[16]. We used the non-informative priors proposed as default in R-INLA and standardized confounders.

Regression parameters are expressed per Interdecile range (IDR) relative risk, i.e. the increase of the relative risk when the level of covariates increases from the $10^{th}$ to $90^{th}$ centile; the posterior mean and 95% credible bounds are given.



For peer review only - http://bmjopen.bmj.com/site/about/guidelines.xhtml

For peer review only

## Results

There were 108,478 cardiovascular and 48,483 respiratory deaths and 24,849 incident lung cancer cases in the study area for 2008-11 (Table 1 and Table S1). We have reported summary descriptive statistics for Standard Mortality/Incidence Rates (SMR/SIRs), metal constituents of PM and confounders, stratifying the wards between the 10th percentile of exposure 90th percentile of PM2.5 Copper. SMRs/SIRs, metal constituents of PM, area-level deprivation, non-white ethnicity and tobacco sales (smoking proxy) were all higher in wards in the $90^{th}$ vs. $10^{th}$ percentile $PM_{2.5}$ copper.

Maps of the spatial distribution of the covariates and elemental concentrations show that highest values were in Greater London Area, with iron and zinc also high in wards with motorways (Figure 2). The percentage population ethnicity for wards had a median of 77% white and 9% Asian ethnicity (predominantly of South Asian origin). Most of the areas with low percentage of White population was concentrated in Greater London, which also had higher percentage of Asian (supplementary material figure S2).

For peer review only

**Table 1**: Descriptive statistics of health outcomes, modelled particulate metal concentrations, deprivation score, and ethnicity covariates for the 1533 wards in the study area in 2008-11, subdivided by $PM_{2.5}$ Copper <10th, 10th-90th and >90th quantile.

| | Cu[1] $PM_{2.5}$ | | | | | | | | | | | |
|---|---|---|---|---|---|---|---|---|---|---|---|---|
| | 10th centile (n=153) | | | | 10th-90th centile (n=1225) | | | | 90th centile (n=154) | | | |
| | 10th centile | mean | median | 90th Centile | 10th centile | mean | median | 90th Centile | 10th centile | mean | median | 90th Centile |
| **Health outcomes** | Standard Mortality/Incidence Ratio (ratio across whole study area =1.00) | | | | | | | | | | | |
| Cardiovascular mortality | 0.57 | 0.83 | 0.86 | 1.13 | 0.72 | 0.99 | 1.01 | 1.33 | 0.63 | 0.96 | 1.00 | 1.35 |
| Respiratory mortality | 0.46 | 0.81 | 0.81 | 1.20 | 0.61 | 0.98 | 1.02 | 1.46 | 0.50 | 0.94 | 0.94 | 1.36 |
| Lung cancer incidence | 0.40 | 0.81 | 0.86 | 1.33 | 0.53 | 0.95 | 0.99 | 1.53 | 0.64 | 1.14 | 1.16 | 1.73 |
| **Area-level confounders** | | | | | | | | | | | | |
| IMD (modified Index of multiple deprivation) | 3.26 | 4.85 | 5.15 | 7.41 | 3.35 | 6.29 | 6.83 | 11.30 | 7.91 | 11.22 | 10.99 | 13.24 |
| % of Asian | 0.01 | 0.01 | 0.01 | 0.02 | 0.02 | 0.07 | 0.12 | 0.26 | 0.07 | 0.11 | 0.15 | 0.31 |



For peer review only - http://bmjopen.bmj.com/site/about/guidelines.xhtml

For peer review only

| | | | | | | | | | | | | |
|---|---|---|---|---|---|---|---|---|---|---|---|---|
| % of White | 0.95 | 0.97 | 0.97 | 0.99 | 0.46 | 0.86 | 0.78 | 0.95 | 0.43 | 0.62 | 0.60 | 0.77 |
| Tobacco expenditure (pounds/ week/inhabitant) | 3.19 | 3.88 | 3.96 | 4.86 | 3.41 | 4.46 | 4.57 | 5.96 | 4.45 | 5.65 | 5.53 | 6.55 |

*Leave one out cross-validation (LOOCV)

**Land Use Regression (LUR)

[1] Cu $PM_{2.5}$ Metals in ng/$m^3$ LOOCV $R^2$ =0.79

For peer review only

The individual linear effect of each elemental constitute of particulate matter evaluated with the Poisson regression adjusted for confounders is displayed in Table 2 and Table S2 in Supplementary Material. Statistically significant associations with PM metal concentrations were identified for cardiovascular and respiratory mortality but not lung cancer incidence. For cardiovascular mortality, copper in the $PM_{2.5}$ fraction was associated with a small increased risk - RR 1.005 (95%CI 1.001, 1.009) per interdecile range (IDR) but iron had an apparent protective association (RR 0.042 95%CI 0.002, 0.995) albeit with extremely high uncertainty. For respiratory mortality, the copper in the $PM_{10}$ fraction had a very small protective association (RR 0.988 95%CI 0.978, 0.998), but $PM_{10}$ zinc was associated with an increased mortality risk (RR 1.136 95%CI 1.010, 1.277).

**Table 2**: Individual effects of metals, estimated with Poisson regression, on cardiovascular mortality, respiratory mortality and lung cancer incidence adjusted for tobacco weekly expenditure, IMD (index of multiple deprivation) and percentage of Asian and White population. Mean and lower and upper bounds of the credible intervals of the inter-decile relative risk (RR).

| | Metal | RR | 95% credible intervals |
|---|---|---|---|
| **Cardiovascular mortality** | Cu $PM_{10}$ | 0.994 | (0.987,1.001) |
| | Fe $PM_{10}$ | 0.319 | (0.037,2.779) |
| | Zn $PM_{10}$ | 1.073 | (0.985,1.169) |
| | Cu $PM_{2.5}$ | 1.005 | (1.001,1.009) |
| | Fe $PM_{2.5}$ | 0.042 | (0.002,0.995) |
| **Respiratory mortality** | Cu $PM_{10}$ | 0.988 | (0.978,0.998) |
| | Fe $PM_{10}$ | 0.649 | (0.033,12.767) |
| | Zn $PM_{10}$ | 1.136 | (1.010,1.277) |
| | Cu $PM_{2.5}$ | 1.003 | (0.998,1.009) |
| | Fe $PM_{2.5}$ | 0.980 | (0.013,72.673) |
| **Lung cancer incidence** | Cu $PM_{10}$ | 0.998 | (0.912,1.091) |
| | Fe $PM_{10}$ | 0.973 | (0.830,1.142) |
| | Zn $PM_{10}$ | 0.995 | (0.910,1.089) |
| | Cu $PM_{2.5}$ | 1.092 | (0.943,1.225) |
| | Fe $PM_{2.5}$ | 0.969 | (0.889,1.057) |



For peer review only - http://bmjopen.bmj.com/site/about/guidelines.xhtml

For peer review only



For peer review only - http://bmjopen.bmj.com/site/about/guidelines.xhtml

The elements were highly correlated: 0.88 for $PM_{2.5}$ elements and 0.82-0.92 for $PM_{10}$ elements (Table 3). For $PM_{10}$ the Pearson correlation between copper and zinc was 0.85, and for $PM_{2.5}$ the correlation between copper and iron was 0.88. The metal constituents showed high correlation with $PM_{2.5}$ and $PM_{10}$ mass concentrations for $PM_{2.5}$ and metals in $PM_{2.5}$ was 0.86-0.89 and 0.73-0.89 for $PM_{10}$ metals; for $PM_{10}$ and $PM_{10}$ metals 0.74-0.88 and 0.86-0.89 for metals in $PM_{2.5}$ (see supplementary table S3).

Thus, it is not possible to definitively attribute an association with one metal element given the inter-dependence.

**Table 3**: Pearson inter-correlation(r) between the particulate matter (PM) metals (n=1533).

| | **$PM_{10}$ Copper** | **$PM_{10}$ Iron** | **$PM_{10}$ Zinc** | **$PM_{2.5}$ Iron** | **$PM_{2.5}$ Copper** |
|---|---|---|---|---|---|
| **$PM_{10}$ Copper** | 1 | | | | |
| **$PM_{10}$ Iron** | 0.85 | 1 | | | |
| **$PM_{10}$ Zinc** | 0.85 | 0.92 | 1 | | |
| **$PM_{2.5}$ Iron** | 0.82 | 0.91 | 0.93 | 1 | |
| **$PM_{2.5}$ Copper** | 0.75 | 0.89 | 0.90 | 0.88 | 1 |

In the model fit, for each group of metals by PM, we have found that area-level deprivation (IMD) and weekly tobacco spend had a clear adverse association with cardiovascular mortality, respiratory mortality and lung cancer incidence (supplementary material Table S3), with moderate high value of VIF. On the contrary, the proportions of White and Asian people in wards was associated with lower risks for the three diseases, suggesting a weak influence of the ethnic composition of the population on mortality/incidence rate.

## Discussion



For peer review only

This ecological study at small area level examined associations between modelled particulate metal (copper, iron and zinc) concentrations in relation to cardiovascular and respiratory mortality and lung cancer incidence in and around Greater London covering 13.6 million population with approximately 110,000 cardiorespiratory deaths and 25,000 new lung cancer cases. While the results did not find evidence of positive association between ambient particulate metal concentrations and lung cancer incidence, Poisson regression suggested copper in the $PM_{2.5}$ fraction was statistically significant associated with increased cardiovascular mortality risk and $PM_{10}$ zinc with respiratory mortality risk. Results for metal constituents were not fully consistent within our study for the same element in $PM_{2.5}$ and $PM_{10}$ size fractions. Metal exposures were highly correlated so it is difficult to definitively attribute an association with one metal element.

Advantages of our study include the use of extremely large datasets with population coverage giving good statistical power to detect even very small associations. Another advantage was the use of standardized exposure models developed from standardized monitoring campaigns to estimate spatial variability in long-term exposures. While exposure data were derived from LUR models that showed good predictability, they may still misclassify true exposure as (i) prediction is good but not perfect (ii) using a model of exposure at residence as a proxy for personal exposure. A limitation in our exposure assessment is the limited number of monitoring sites, 20, which potentially can lead to overfitting of the developed LUR models[17]. Providing that densities of measurement sites and estimation sites (wards) are similar, Szpiro and Paciorek (2013)[18] show that in case of over-smoothing of the exposure, the association between outcomes and exposure may be underestimated. In our case, over-smoothing likely occurs and this issue may partially explain our difficulty to show evidence of adverse associations between health outcomes and exposures to particulate elements. As most other ambient air pollution studies, we use outdoor concentration of pollutants at residence, without taking into account indoor levels, travel exposure or places of work. The correlation between indoor and outdoor concentration is high for fine particulate ($PM_{2.5}$)[19], suggesting that ignoring the indoor concentration is a small issue. However, in the London region, the difference of exposure at home and workplace may be different, since a part of the population living in suburban areas work in the city center, where exposures are higher. Another limitation is that we used LUR models predicting particulate metals in 2010-2011 to look at associations with mortality during 2008-11. Our exposure estimates should also be representative of the preceding two years and should capture deaths related to short- and intermediate-long term



For peer review only - http://bmjopen.bmj.com/site/about/guidelines.xhtml

influences. However, we used an ecological study design with limited ability to control for confounders at the individual level.

There are a limited number of other health studies looking at copper, zinc and iron metal components of particulates. Three studies looking at long-term effects using similarly derived estimates from the TRANSPHORM project as used here but much smaller numbers of health events than this study, found significant associations with inflammatory markers in blood but not health events. Hampel et al. [8] found positive statistically significant associations between $PM_{2.5}$ copper and $PM_{10}$ iron with high-sensitivity C-reactive protein and $PM_{2.5}$ zinc with fibrinogen in five European cohorts with available biomarkers (>17,000 measurements). Wolf et al [20] found elevated but non-significant positive associations with copper, zinc and iron constituents of particulates ($PM_{10}$ or $PM_{2.5}$) with incident coronary events in 11 cohorts (5,157 events), while Wang et al. [9] did not find long-term positive associations with cardiovascular mortality (9545 deaths) in 19 European cohorts where exposure results from a single year were applied over 2-20 years follow-up, in some cases retrospectively. A further study, the California teachers study [21] found positive and significant associations between $PM_{2.5}$ copper estimated in 2001-7 and contemporaneous ischemic heart disease deaths (1085 events) and elevated but non-significant associations with $PM_{2.5}$ iron and other metals.

We did not find associations with lung cancer incidence. While toxicological studies suggest that metals in airborne particulates are genotoxic[22], the reason we did not find an association even in our large sample size may be because our exposure measures relate to a similar time frame as the health outcome. Studies finding associations of particulates with lung cancer have typically considered 10 or more years follow-up[23].

Short-term associations of metal components of particulates with mortality were examined in a systematic review of time series studies of fine-particle components and health published up to 2013 [24]. Zinc, indicative of road dust and possibly a result of tyre wear, was associated with daily mortality in eight of eleven studies included in the review. The subsequently published MED-PARTICLES time-series analysis in five European cities Basagaña et al. [17] found positive significant short-term associations with $PM_{10}$ copper iron and zinc and $PM_{2.5}$ iron with



For peer review only - http://bmjopen.bmj.com/site/about/guidelines.xhtml

cardiovascular hospitalizations and $PM_{10}$ and $PM_{2.5}$ zinc for respiratory disease hospitalizations, but no significant associations were seen for mortality.

The reason that results for metal constituents of particulates are not completely consistent across studies, may be that metal concentrations serve as a proxy for oxidative potential[25]. Within the study area and in the analysis, the TRANSPHORM metal particulate measurements used to derive the land use regression models were highly correlated with oxidative potential of the particulates as measured using ascorbate (Pearson r = 0.93 for copper, 0.95 for iron, 0.67 for zinc)[25]. The high correlations between metal constituents of particulates raise the possibility that observed associations for one metal actually relate to another element that was better estimated. The high correlations also preclude conducting multi-pollutant analyses using Poisson regression.

## Conclusion

We found positive and significant associations suggestive of small increased risk of cardiovascular and respiratory mortality but not lung cancer incidence in Greater London and surroundings in relation to metal concentrations of ambient particulate matter, which are likely derived from non-tailpipe road traffic emissions (brake and tyre-wear). Findings are consistent with a previous study finding associations of particulate metals with inflammatory markers, but further work is needed to better define exposures to airborne metal elements and non-tailpipe emissions.

## References

1. Pope CA, Burnett RT, Thurston GD, et al. Cardiovascular Mortality and Long-Term Exposure to Particulate Air Pollution. *Epidemiological Evidence of General Pathophysiological Pathways of Disease* 2004;109(1):71-77. doi: 10.1161/01.Cir.0000108927.80044.7f
2. Puett RC, Hart JE, Suh H, et al. Particulate Matter Exposures, Mortality, and Cardiovascular Disease in the Health Professionals Follow-up Study. *Environmental Health Perspectives* 2011;119(8):1130-35. doi: 10.1289/ehp.1002921



For peer review only - http://bmjopen.bmj.com/site/about/guidelines.xhtml

3. Zhang LW, Chen X, Xue XD, et al. Long-term exposure to high particulate matter pollution and cardiovascular mortality: a 12-year cohort study in four cities in northern China. *Environ Int* 2014;62:41-7. doi: 10.1016/j.envint.2013.09.012 [published Online First: 2013/10/29]
4. Brook RD, Rajagopalan S, Pope CA, et al. Particulate Matter Air Pollution and Cardiovascular Disease. *An Update to the Scientific Statement From the American Heart Association* 2010;121(21):2331-78. doi: 10.1161/CIR.0b013e3181dbece1
5. Crouse DL, Peters PA, van Donkelaar A, et al. Risk of nonaccidental and cardiovascular mortality in relation to long-term exposure to low concentrations of fine particulate matter: a Canadian national-level cohort study. *Environ Health Perspect* 2012;120(5):708-14. doi: 10.1289/ehp.1104049 [published Online First: 2012/02/09]
6. Beelen R, Hoek G, van den Brandt PA, et al. Long-Term Effects of Traffic-Related Air Pollution on Mortality in a Dutch Cohort (NLCS-AIR Study). *Environmental Health Perspectives* 2008;116(2):196-202. doi: 10.1289/ehp.10767
7. Li H, Qian X, Wang Qg. Heavy Metals in Atmospheric Particulate Matter: A Comprehensive Understanding Is Needed for Monitoring and Risk Mitigation. *Environmental Science & Technology* 2013;47(23):13210-11. doi: 10.1021/es404751a
8. Hampel R, Peters A, Beelen R, et al. Long-term effects of elemental composition of particulate matter on inflammatory blood markers in European cohorts. *Environment International* 2015;82:76-84. doi: https://doi.org/10.1016/j.envint.2015.05.008
9. Wang M, Beelen R, Stafoggia M, et al. Long-term exposure to elemental constituents of particulate matter and cardiovascular mortality in 19 European cohorts: Results from the ESCAPE and TRANSPHORM projects. *Environment International* 2014;66:97-106. doi: https://doi.org/10.1016/j.envint.2014.01.026
10. Eeftens M, Tsai M-Y, Ampe C, et al. Spatial variation of PM2.5, PM10, PM2.5 absorbance and PMcoarse concentrations between and within 20 European study areas and the relationship with NO2 - Results of the ESCAPE project. *Atmospheric Environment* 2012;62:303-17. doi: 10.1016/j.atmosenv.2012.08.038
11. Tsai M-Y, Hoek G, Eeftens M, et al. Spatial variation of PM elemental composition between and within 20 European study areas — Results of the ESCAPE project. *Environment International* 2015;84:181-92. doi: https://doi.org/10.1016/j.envint.2015.04.015
12. de Hoogh K, Wang M, Adam M, et al. Development of Land Use Regression Models for Particle Composition in Twenty Study Areas in Europe. *Environmental Science & Technology* 2013;47(11):5778-86. doi: 10.1021/es400156t
13. deprivation Eio. https://www.gov.uk/government/statistics/english-indices-of-deprivation-2010, 2010.
14. Adams J, White M. Removing the health domain from the Index of Multiple Deprivation 2004—effect on measured inequalities in census measure of health. *Journal of Public Health* 2006;28(4):379-83. doi: 10.1093/pubmed/fdl061
15. Besag J, York J, Mollié A. Bayesian image restoration, with two applications in spatial statistics. *Annals of the Institute of Statistical Mathematics* 1991;43(1):1-20. doi: 10.1007/bf00116466
16. Martins TG, Simpson D, Lindgren F, et al. Bayesian computing with INLA:New features. *Computational Statistics & Data Analysis* 2013;67:68-83. doi: 10.1016/j.csda.2013.04.014
17. Basagaña X, Rivera M, Aguilera I, et al. Effect of the number of measurement sites on land use regression models in estimating local air pollution. *Atmospheric Environment* 2012;54:634-42. doi: https://doi.org/10.1016/j.atmosenv.2012.01.064



For peer review only - http://bmjopen.bmj.com/site/about/guidelines.xhtml

18. Szpiro AA, Paciorek CJ. Measurement error in two-stage analyses, with application to air pollution epidemiology. *Environmetrics* 2013;24(8):501-17. doi: 10.1002/env.2233
19. Brunekreef B, Holgate ST. Air pollution and health. *The Lancet* 2002;360(9341):1233-42. doi: https://doi.org/10.1016/S0140-6736(02)11274-8
20. Wolf K, Stafoggia M, Cesaroni G, et al. Long-term Exposure to Particulate Matter Constituents and the Incidence of Coronary Events in 11 European Cohorts. *Epidemiology* 2015;26(4):565-74. doi: 10.1097/ede.0000000000000300
21. Ostro B, Hu J, Goldberg D, et al. Associations of Mortality with Long-Term Exposures to Fine and Ultrafine Particles, Species and Sources: Results from the California Teachers Study Cohort. *Environmental Health Perspectives* 2015;123(6):549-56. doi: 10.1289/ehp.1408565
22. Bocchi C, Bazzini C, Fontana F, et al. Characterization of urban aerosol: Seasonal variation of genotoxicity of the water-soluble portion of PM2.5 and PM1. *Mutation Research/Genetic Toxicology and Environmental Mutagenesis* 2019;841:23-30. doi: https://doi.org/10.1016/j.mrgentox.2019.04.005
23. Raaschou-Nielsen O, Andersen ZJ, Beelen R, et al. Air pollution and lung cancer incidence in 17 European cohorts: prospective analyses from the European Study of Cohorts for Air Pollution Effects (ESCAPE). *The Lancet Oncology* 2013;14(9):813-22. doi: https://doi.org/10.1016/S1470-2045(13)70279-1
24. Atkinson RW, Analitis A, Samoli E, et al. Short-term exposure to traffic-related air pollution and daily mortality in London, UK. *Journal Of Exposure Science And Environmental Epidemiology* 2015;26:125. doi: 10.1038/jes.2015.65

https://www.nature.com/articles/jes201565#supplementary-information
25. Gulliver J, Morley D, Dunster C, et al. Land use regression models for the oxidative potential of fine particles (PM2.5) in five European areas. *Environmental Research* 2018;160:247-55. doi: https://doi.org/10.1016/j.envres.2017.10.002



For peer review only - http://bmjopen.bmj.com/site/about/guidelines.xhtml

## Figures

**Figure 1.** Study Area compromising London and Oxford areas, with major roads and motorways. In the inset the area localization with regard to England map. Contains National Statistics data © Crown copyright and database right 2018; Contains OS data © Crown copyright and database right 2018. All rights reserved.

**Figure 2.** Maps of the metal exposures population weighted by ward. Contains National Statistics data © Crown copyright and database right 2018; Contains OS data © Crown copyright and database right 2018. All rights reserved.



For peer review only - http://bmjopen.bmj.com/site/about/guidelines.xhtml

For peer review only



For peer review only - http://bmjopen.bmj.com/site/about/guidelines.xhtml

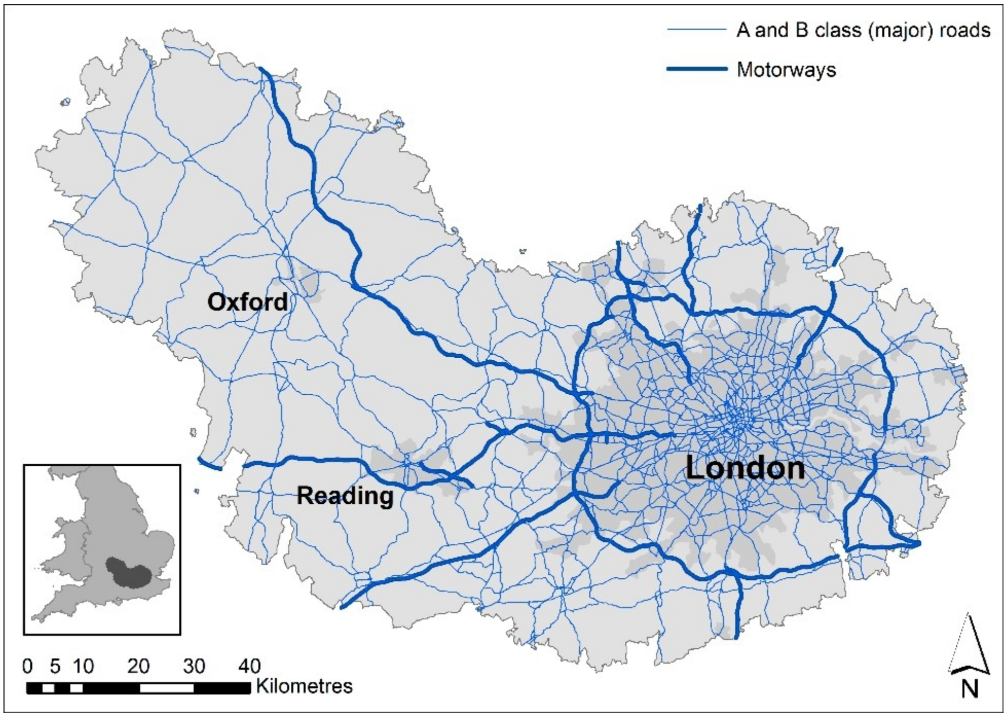


Study Area compromising London and Oxford areas, with major roads and motorways. In the inset the area localization with regard to England map. Contains National Statistics data © Crown copyright and database right 2018; Contains OS data © Crown copyright and database right 2018. All rights reserved.

112x79mm (220 x 220 DPI)

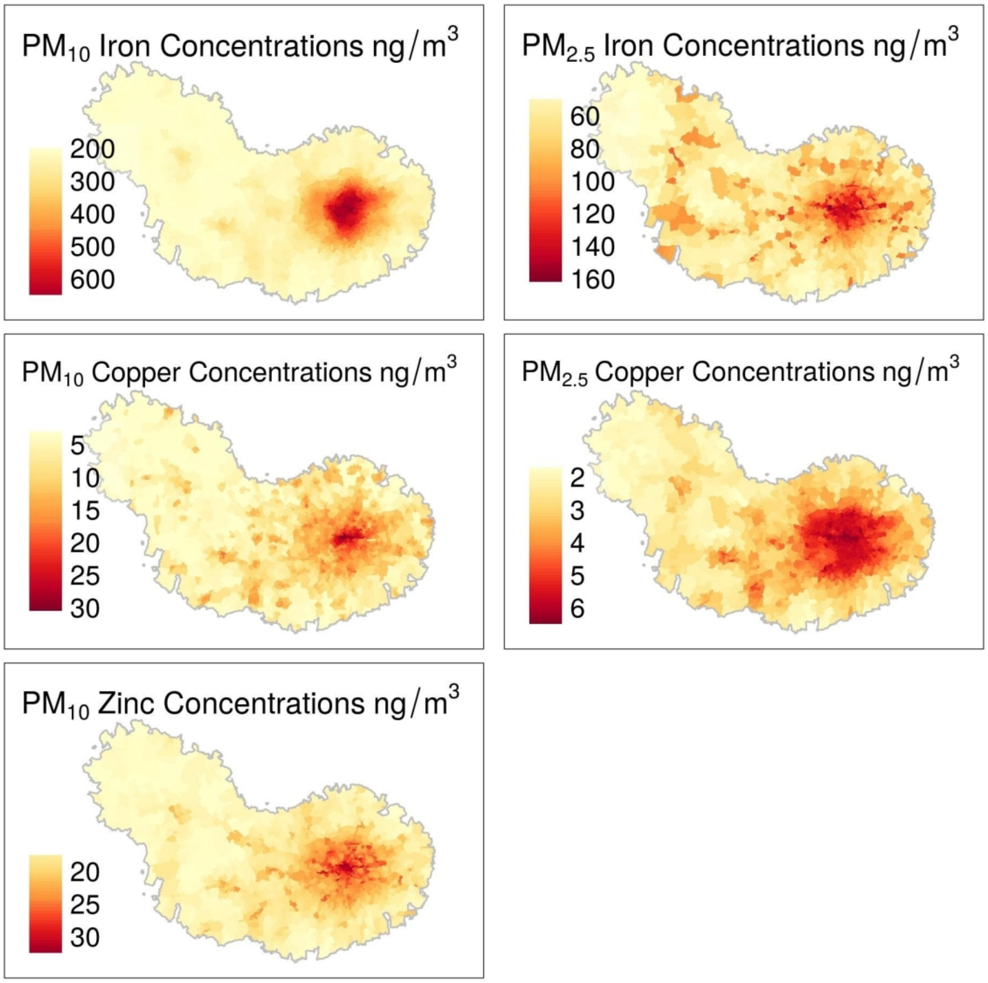


Maps of the metal exposures population weighted by ward. Contains National Statistics data © Crown copyright and database right 2018; Contains OS data © Crown copyright and database right 2018. All rights reserved.

155x153mm (300 x 300 DPI)

Supplementary material to:

**Associations between metal constituents of ambient particulate matter and mortality in England; an ecological study**

**Table S1**: Descriptive statistics of health outcomes, modelled particulate metal concentrations, deprivation score, and ethnicity covariates for the 1533 wards in the study area in 2008-11.

| | 10th centile | mean | median | 90th centile | LOOCV* $R^2$ (for LUR**) |
|---|---|---|---|---|---|
| **Health outcomes** | Rates of health outcome (number of cases per hundred thousand people) | | | | |
| Cardiovascular mortality | 117.50 | 215.97 | 203.20 | 327.87 | |
| Respiratory mortality | 42.85 | 96.34 | 87.85 | 160.41 | |
| Lung cancer incidence | 25.06 | 48.44 | 45.75 | 75.86 | |
| **Modelled metal concentrations using LUR** | Metals in $ng/m^3$ | | | | |
| Cu $PM_{10}$ | 7.0 | 13.3 | 13.1 | 19.8 | 0.95 |
| Fe $PM_{10}$ | 223.2 | 378.9 | 357.0 | 596.7 | 0.95 |
| Zn $PM_{10}$ | 113.5 | 135.2 | 139.5 | 153.0 | 0.77 |
| Cu $PM_{2.5}$ | 2.6 | 4.3 | 4.6 | 5.7 | 0.79 |
| Fe $PM_{2.5}$ | 51.6 | 86.8 | 82.8 | 129.0 | 0.92 |
| **Area-level confounders** | | | | | |
| Deprivation (modified IMD) | 3.45 | 7.08 | 6.47 | 11.78 | |
| % of Asian | 2 | 13 | 9 | 33 | |
| % of White | 38 | 72 | 77 | 95 | |
| Tobacco expenditure (pounds/week/inhabitant) | 3.40 | 4.61 | 4.48 | 6.03 | |

*LOOCV Leave one out cross validation

** LUR Land Use Regression

**Table S2:** Poisson regression confounder effects from the two models (i) using metals from $PM_{10}$ and (ii) metals from $PM_{2.5}$ for all the health outcomes. Mean, lower and upper bound of the 95% credible interval (CI) of the inter-decile relative risk (RR).

| Outcomes | Model | Confounders | RR | CI 95% |
|---|---|---|---|---|
| Cardiovascular mortality | All Metals in $PM_{10}$ VIF[2]=9.14 | IMD[1] | 1.098 | (1.02,1.182) |
| | | % Asian | 0.982 | (0.921,1.046) |
| | | % White | 0.817 | (0.729,0.915) |
| | | Tobacco expenditure | 1.197 | (1.133,1.265) |
| | All Metals in $PM_{2.5}$ VIF[2]=9.04 | IMD[1] | 1.095 | (1.02,1.177) |
| | | % Asian | 0.987 | (0.926,1.052) |
| | | % White | 0.824 | (0.737,0.922) |
| | | Tobacco expenditure | 1.192 | (1.135,1.253) |
| | | IMD[1] | 1.188 | (1.073,1.315) |

For peer review only

| | | | | |
|---|---|---|---|---|
| Respiratory mortality | All Metals in PM10 VIF[2]=8.93 | % Asian | 0.887 | (0.813,0.967) |
| | | % White | 0.822 | (0.704,0.959) |
| | | Tobacco expenditure | 1.301 | (1.206,1.403) |
| | All Metals in PM2.5 VIF[2]=8.81 | IMD[1] | 1.183 | (1.07,1.306) |
| | | % Asian | 0.892 | (0.817,0.973) |
| | | % White | 0.846 | (0.725,0.986) |
| | | Tobacco expenditure | 1.301 | (1.214,1.393) |
| Lung cancer incidence | All Metals in PM10 VIF =8.60 | IMD[1] | 1.390 | (1.261,1.532) |
| | | % Asian | 0.851 | (0.790,0.916) |
| | | % White | 0.932 | (0.818,1.062) |
| | | Tobacco expenditure | 1.472 | (1.366,1.586) |
| | All Metals in PM2.5 VIF[2]=7.72 | IMD[1] | 1.404 | (1.276,1.544) |
| | | % Asian | 0.846 | (0.786,0.910) |
| | | % White | 0.955 | (0.839,1.086) |
| | | Tobacco expenditure | 1.468 | (1.373,1.569) |

[1]IMD Indices of multiple deprivation
[2]VIF Variance Inflation Factor

**Table S3:** Pearson Correlation(r) between the adjusted annual mean concentrations of PM-metals and the adjusted annual mean PM concentrations ($PM_{2.5}$ and $PM_{10}$).

| N=1533 | | $PM_{2.5}$ CU | $PM_{2.5}$ FE | $PM_{10}$ CU | $PM_{10}$ FE | $PM_{10}$ ZN | $PM_{2.5}$ | $PM_{10}$ |
|---|---|---|---|---|---|---|---|---|
| **$PM_{2.5}$** | Correlation | 0.86 | 0.89 | 0.89 | 0.89 | 0.73 | 1.00 | 0.92 |
| **$PM_{10}$** | Correlation | 0.82 | 0.87 | 0.86 | 0.88 | 0.74 | 0.92 | 1.00 |

All the correlation significant at p< 0.001

For peer review only

**Figure S1.** A graphical presentation of the confounding and causal mechanism, linking exposure and adverse health outcomes.

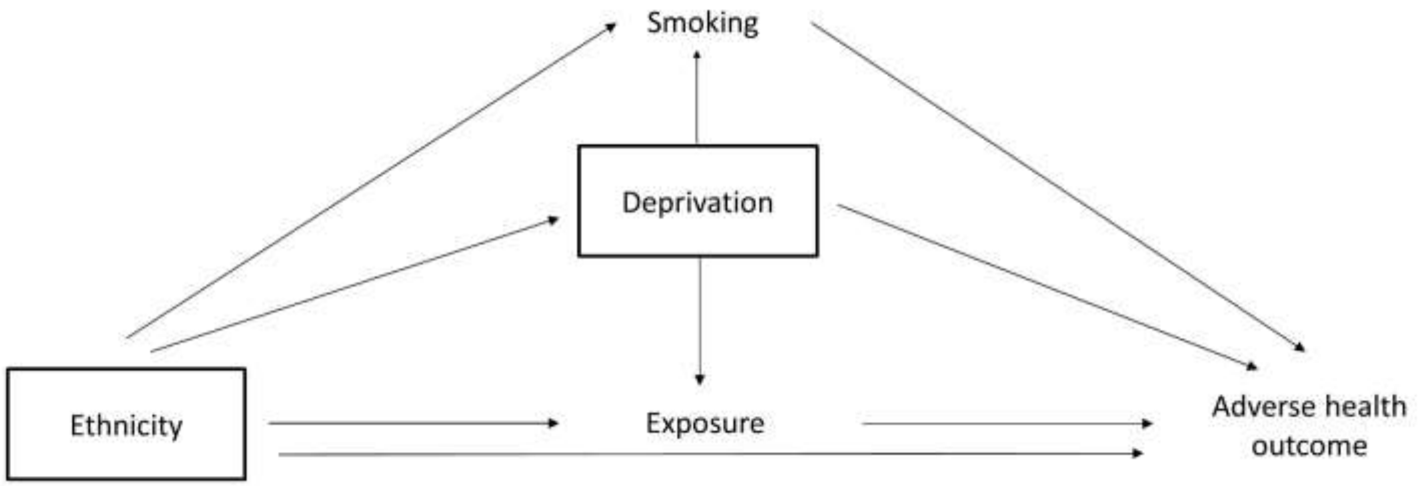


only

**Figure S2.** Maps of the confounders in quintiles: proportion of Asian people, proportion of white people, modified index of multiple deprivations, and tobacco spends. Contains National Statistics data © Crown copyright and database right 2018; Contains OS data © Crown copyright and database right 2018. All rights reserved.

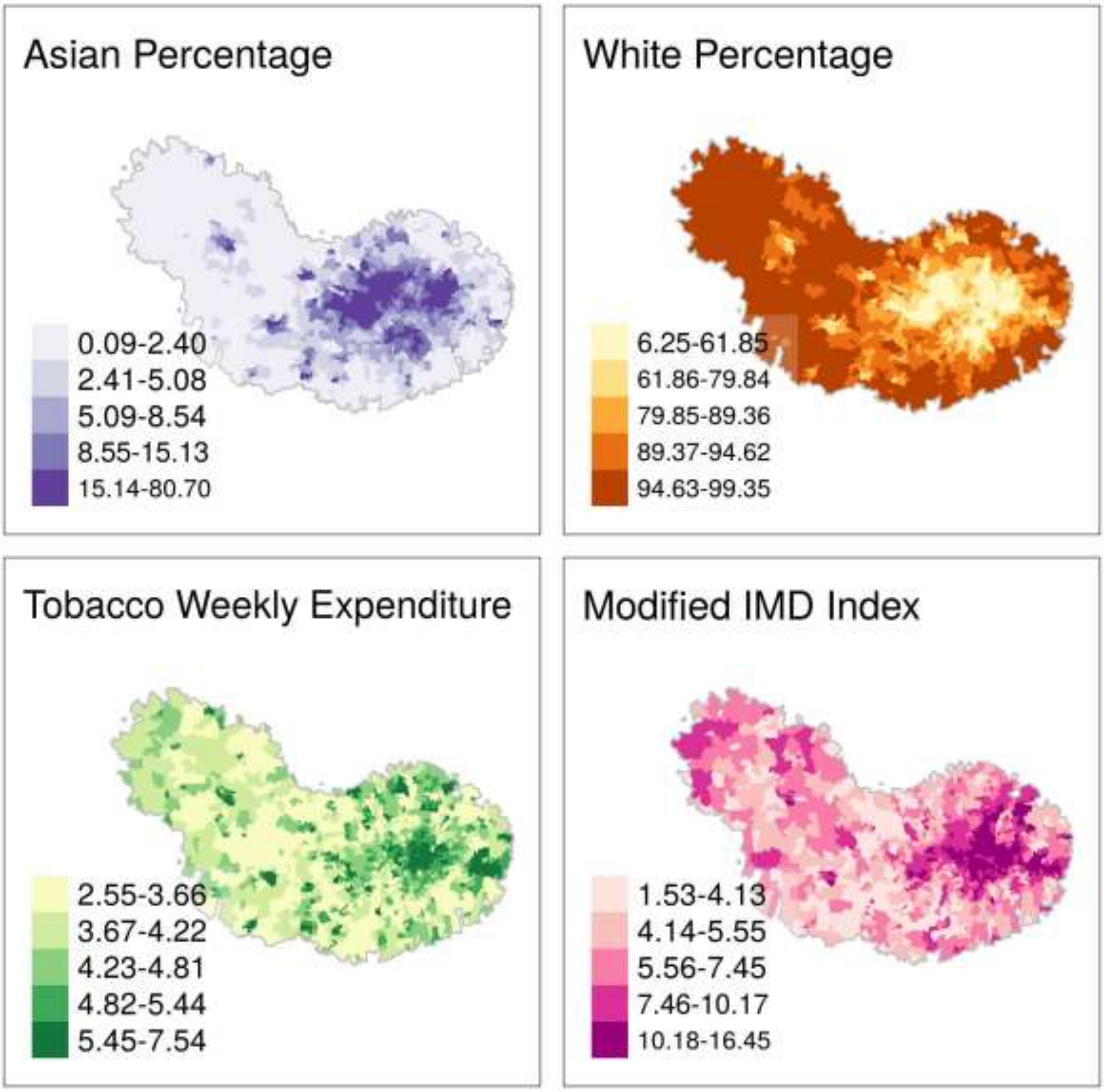

**STROBE 2007 (v4) Statement—Checklist of items that should be included in reports of *cross-sectional studies***

| **Section/Topic** | **Item #** | **Recommendation** | **Reported on page #** |
|---|---|---|---|
| **Title and abstract** | 1 | (*a*) Indicate the study's design with a commonly used term in the title or the abstract | 1 |
| | | (*b*) Provide in the abstract an informative and balanced summary of what was done and what was found | 3 |
| **Introduction** | | | |
| Background/rationale | 2 | Explain the scientific background and rationale for the investigation being reported | 5 |
| Objectives | 3 | State specific objectives, including any prespecified hypotheses | 5 |
| **Methods** | | | |
| Study design | 4 | Present key elements of study design early in the paper | 5 |
| Setting | 5 | Describe the setting, locations, and relevant dates, including periods of recruitment, exposure, follow-up, and data collection | 5-6 |
| Participants | 6 | (*a*) Give the eligibility criteria, and the sources and methods of selection of participants | 5-6, Figure 1 |
| Variables | 7 | Clearly define all outcomes, exposures, predictors, potential confounders, and effect modifiers. Give diagnostic criteria, if applicable | 5-6, Figure 2 and supplementary Figure S1 |
| Data sources/ measurement | 8* | For each variable of interest, give sources of data and details of methods of assessment (measurement). Describe comparability of assessment methods if there is more than one group | 5-6 |

For peer review only

| | | | |
|---|---|---|---|
| Bias | 9 | Describe any efforts to address potential sources of bias | 5, 7 |
| Study size | 10 | Explain how the study size was arrived at | Figure 1 |
| Quantitative variables | 11 | Explain how quantitative variables were handled in the analyses. If applicable, describe which groupings were chosen and why | 5-6 |
| Statistical methods | 12 | (*a*) Describe all statistical methods, including those used to control for confounding | 6 |
| | | (*b*) Describe any methods used to examine subgroups and interactions | |
| | | (*c*) Explain how missing data were addressed | No missing data! |
| | | (*d*) If applicable, describe analytical methods taking account of sampling strategy | NA – used all deaths, all population in area |
| | | (*e*) Describe any sensitivity analyses | None conducted |
| **Results** | | | |
| Participants | 13* | (a) Report numbers of individuals at each stage of study—eg numbers potentially eligible, examined for eligibility, confirmed eligible, included in the study, completing follow-up, and analysed | Table 1 and table 2 |
| | | (b) Give reasons for non-participation at each stage | NA |
| | | (c) Consider use of a flow diagram | NA |
| Descriptive data | 14* | (a) Give characteristics of study participants (eg demographic, clinical, social) and information on exposures and potential confounders | Supplementary Table S1 and Figure S1, 6 |
| | | (b) Indicate number of participants with missing data for each variable of interest | NA |
| Outcome data | 15* | Report numbers of outcome events or summary measures | 6 |

For peer review only

| | | | |
|---|---|---|---|
| Main results | 16 | (*a*) Give unadjusted estimates and, if applicable, confounder-adjusted estimates and their precision (eg, 95% confidence interval). Make clear which confounders were adjusted for and why they were included | 9-10, Table 1 and 3, Supplementary table S1 |
| | | (*b*) Report category boundaries when continuous variables were categorized | NA (no categorization) |
| | | (*c*) If relevant, consider translating estimates of relative risk into absolute risk for a meaningful time period | NA |
| Other analyses | 17 | Report other analyses done—eg analyses of subgroups and interactions, and sensitivity analyses | NA |
| **Discussion** | | | |
| Key results | 18 | Summarise key results with reference to study objectives | 7 |
| Limitations | 19 | Discuss limitations of the study, taking into account sources of potential bias or imprecision. Discuss both direction and magnitude of any potential bias | 8 |
| Interpretation | 20 | Give a cautious overall interpretation of results considering objectives, limitations, multiplicity of analyses, results from similar studies, and other relevant evidence | 8 |
| Generalisability | 21 | Discuss the generalisability (external validity) of the study results | 8 |
| **Other information** | | | |
| Funding | 22 | Give the source of funding and the role of the funders for the present study and, if applicable, for the original study on which the present article is based | 2 |

*Give information separately for cases and controls in case-control studies and, if applicable, for exposed and unexposed groups in cohort and cross-sectional studies.

For peer review only

**Note:** An Explanation and Elaboration article discusses each checklist item and gives methodological background and published examples of transparent reporting. The STROBE checklist is best used in conjunction with this article (freely available on the Web sites of PLoS Medicine at http://www.plosmedicine.org/, Annals of Internal Medicine at http://www.annals.org/, and Epidemiology at http://www.epidem.com/). Information on the STROBE Initiative is available at www.strobe-statement.org.

For peer review only
